# Synthesis and characterisation of novel dipyridine and pyridinyl benzoquinoline complexes of zinc and nickel

**DOI:** 10.1039/d6dt00659k

**Published:** 2026-05-26

**Authors:** Lars Killian, Anne Olarte Loyo, Roy R. P. A. M. de Ridder, Martin Lutz, Arnaud Thevenon

**Affiliations:** a Organic Chemistry and Catalysis, Institute for Sustainable and Circular Chemistry, Faculty of Science, Utrecht University Universiteitsweg 99 3584 CG Utrecht The Netherlands a.a.thevenon-kozub@uu.nl; b Structural Biochemistry, Bijvoet Centre for Biomolecular Research, Faculty of Science, Utrecht University Universiteitsweg 99 3584 CG Utrecht The Netherlands

## Abstract

Dipyridine and pyridinyl benzoquinoline ligands and their coordination chemistry with nickel and zinc are described. The pyridinyl benzoquinoline ligand was synthesised through the photochemical Mallory reaction of the dipyridine ligand. The zinc complexes adopt distorted tetrahedral geometries, featuring one coordinated dipyridine or pyridinyl benzoquinoline ligand, with two chlorides completing the first coordination sphere. In contrast, the nickel complexes adopt octahedral geometries with two bidentate ligands and two coordinating chlorides per metal centre. Using cyclic voltammetry, the redox behaviour of all complexes was investigated, revealing both ligand- and metal-based reduction, as well as surprising catalytic reactivity with DCM. Together, this work explores the synthesis and coordination chemistry of two new ligands of the uncommon dipyridine family.

## Abstract 2

Dipyridine and pyridinyl benzoquinoline ligands and their coordination chemistry with nickel and zinc are described.

## Graphical abstract

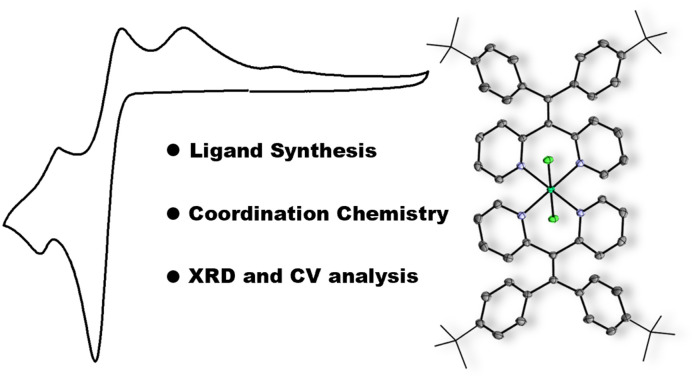

## Introduction

**Fig. 1:**
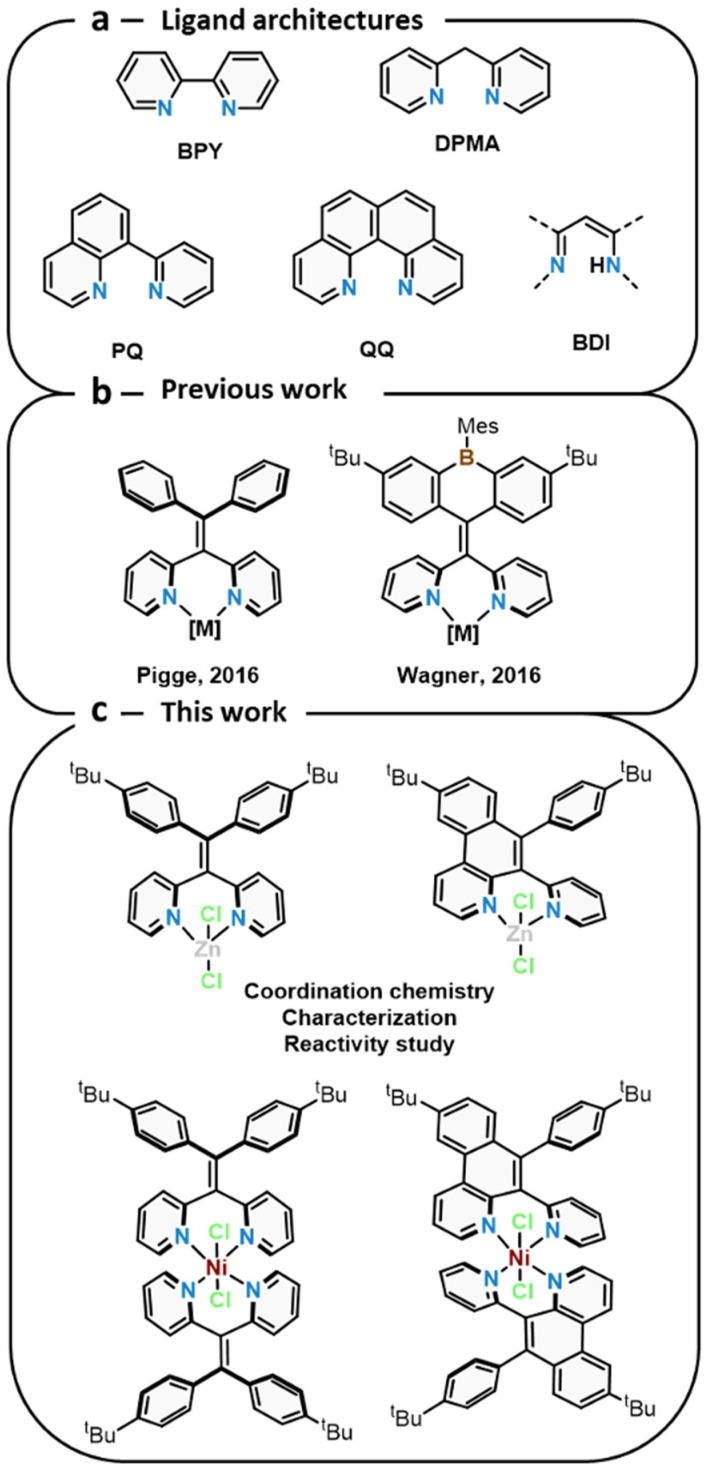
An overview of different binding pockets and ligands related to the work presented herein.

Whereas bipyridine (BPY) ligands are ubiquitous in coordination chemistry and homogeneous catalysis,^1^ the related dipyridine ligands, where the pyridine units are separated by a single carbon atom are far less commonly explored (Fig. 1a). The dipyridylmethane (DPMA) ligand specifically has been described as a “forgotten ligand in coordination chemistry”.^2^ Related to dipyridine ligands are quinolinoquinoline (QQ)^3^ and pyridinyl quinoline (PQ)^4^ ligands (Fig. 1a). These ligands generally feature the same binding pocket as the dipyridine ligand, but the bridging carbon atom is now part of the quinoline system. The binding pocket also shows some similarities with β-diketiminate (BDI) ligands (Fig. 1a), which also feature a N–C–C–C–N binding motif but typically act as monoanionic ligands in contrast with the neutral dipyridine. Several structural features stand out when comparing dipyridine-type ligands to these related motifs. Compared to bipyridines, the extra sp^2^ carbon atom influences the angle at which the pyridine units approach the metal centre by forcing the nitrogen donor atoms to face more towards each other. This more narrow angle in the binding motif leads to a geometry in which the pyridine rings do not occupy the same plane, but are angled towards the metal centre (Fig. 1c). The nature of the bridging carbon atom can be used to tune the electronics and geometry of the binding pocket. Common variations include introducing pendant (coordinating) functional groups or an sp^2^ hybridised bridging carbon; in some cases, a deprotonated (anionic) binding pocket has also been reported.^5–7^ Notably, pendant coordinating groups on the bridging carbon have been exploited to promote oxidative addition at a high-valent nickel centre.^8,9^

Despite still being relatively obscure, the versatile binding pocket has been used as a ligand in an increasing number of complexes in recent years. For example, several anti-cancer complexes have been formulated featuring a dipyridine-type ligand.^10–13^ Dipyridine compounds featuring a tetraarylethylene core (Fig. 1b) have been used by the group of Pigge as fluorescent dyes,^14–17^ since these molecules exhibit aggregation induced emission properties.^18^ By exploiting the metal binding properties of the dipyridine ligand, the dyes can be used as sensors for several metal ions^15,16^ and coordinating anions.^17^

The tetraarylethylene motive lends itself well as a platform for the synthesis of polyaromatic hydrocarbons (PAHs). The inherent stilbene motif (doubly) present in these molecules allows for facile photochemical cyclodehydrogenation through the Mallory reaction by which the π-system can be extended to arrive at larger PAHs as shown by the group of Wagner in 2015.^19^ In 2016, the same group reported a tetraarylethylene-type dipyridine, aiming to convert it to a QQ-type nanographene compound through a double Mallory reaction.^20^ However, the lone-pair repulsion of the pyridinic nitrogen atoms forces the pyridine unit to adopt a conformation unsuitable for the desired cyclodehydrogenation reaction. This effect was surprisingly not mitigated by the addition of a protic acid such as MeSO_3_H, which was hypothesized to minimize the nitrogen lone-pair repulsion by protonation of the basic binding pocket. The group further explored metal-templating to mitigate lone-pair repulsion, for which they prepared the palladium dichloride complex of the dipyridine ligand. However, this also did not have the desired effect, and photochemical cyclodehydrogenation was unsuccessful.

Given our interest in the development of novel (transition) metal ligands bearing extended π-systems,^21^ we were inspired by the work of Wagner and the small number of dipyridine-type ligands in the literature to explore the synthesis and coordination chemistry of new dipyridine ligands as well as their possible π-extension to PQ- and QQ-type ligands.

## Results & discussion

### Synthesis and structural characterization

**Scheme 1:**
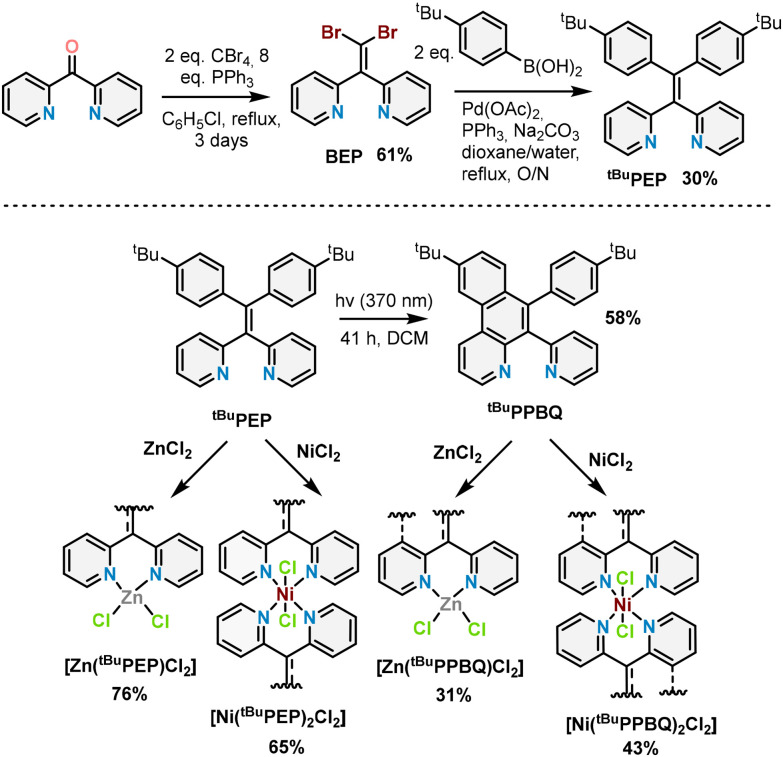
Synthesis of ^*t*Bu^PEP and ^*t*Bu^PPBQ, as well as their nickel complexes [Ni(^*t*Bu^PEP)_2_Cl_2_] and [Ni(^*t*Bu^PPBQ)_2_Cl_2_], and zinc complexes [Zn(^*t*Bu^PEP)Cl_2_] and [Zn(^*t*Bu^PPBQ)Cl_2_].

The synthesis of the target ligands was accomplished according to (modified) literature protocols.^15,16^ Firstly, dipyridyl ketone was treated with CBr_4_ and PPh_3_ to form 2,2′-(2,2-dibromoethene-1,1-diyl)dipyridine (BEP) in 61% yield, followed by a double Suzuki coupling to form 2,2′-(2,2-bis(4-(*tert*-butyl)phenyl)ethene-1,1-diyl)dipyridine (^*t*Bu^PEP) in 30% yield (Scheme 1). Next, 9-(*tert*-butyl)-6-(4-(*tert*-butyl)phenyl)-5-(pyridin-2-yl)benzo[*f*]quinoline (^*t*Bu^PPBQ) was obtained in 58% yield by means of a Mallory reaction, using a 370 nm lamp (Scheme 1). Formation of the product during the reaction is evident from the fluorescence emission of the product (Fig. S30). Similarly to what was reported by Wagner *et al.*, the formation of the double cyclodehydrogenation product was not observed under photochemical conditions.^20^ In our attempts to obtain the doubly cyclodehydrogenated product we also investigated templating using a Lewis acid (BF_3_·OEt_2_) or metal centre (NiCl_2_ and ZnCl_2_, *vide infra*) but this again did not allow for the formation of the desired species under irradiation.

Complexation of ligand ^*t*Bu^PEP to ZnCl_2_ was accomplished by refluxing ZnCl_2_ and the ligand together in EtOH (Scheme 1). After recrystallisation from EtOH, complex [Zn(^*t*Bu^PEP)Cl_2_] was obtained in a 76% isolated yield. Crystals suitable for single-crystal X-ray structure determination were grown from DCM/PE, showing two independent molecules of [Zn(^*t*Bu^PEP)Cl_2_] in the asymmetric unit of which only one is discussed here, since bond metrics are only marginally different (Fig. S42).

**Fig. 2:**
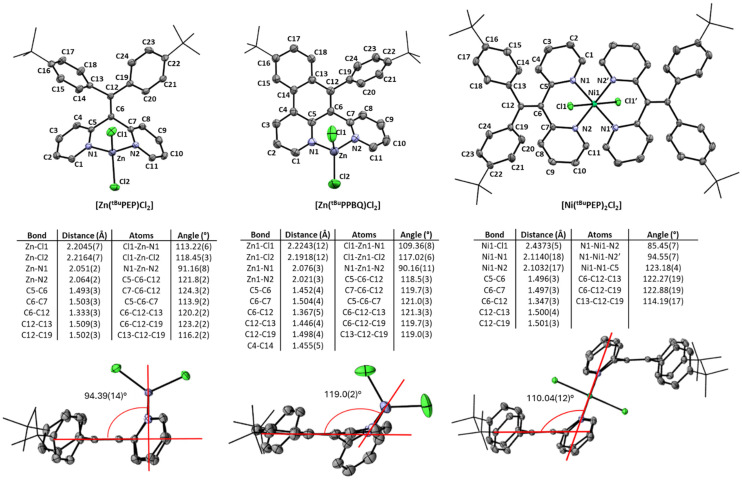
Labelled displacement ellipsoid plot (50% probability) of the asymmetric unit of [Zn(^*t*Bu^PEP)Cl_2_] (left), [Zn(^*t*Bu^PPBQ)Cl_2_] (middle) and [Ni(^*t*Bu^PEP)_2_Cl_2_] (right). The hydrogen atoms and co-crystallized solvent molecules are omitted, and *tert*-butyl groups are shown as wireframe for clarity. For [Zn(^*t*Bu^PEP)Cl_2_], the asymmetric unit shows two independent molecules, of which only one is shown. For the data of the other molecule, see Table S1. In [Ni(^*t*Bu^PEP)_2_Cl_2_] the Ni is on an inversion centre. Selected (bond) distances and angles are given in tables below each structure. For the side-views, the angle shown in red is calculated for of [Zn(^*t*Bu^PEP)Cl_2_] as the angle between the average plane through C5, C6, C7 and C12 and the plane defined by N1, Zn1 and N2; for [Zn(^*t*Bu^PPBQ)Cl_2_] as the angle between the average plane through C6, C12, C13 and C19 and the plane defined by N1, Zn and N2; for [Ni(^*t*Bu^PEP)_2_Cl_2_] as the angle between the average plane through C5, C6, C7 and C12 and the plane defined by N1, Ni1 and N2.

The crystal structure shows that the zinc centre is coordinated to one ^*t*Bu^PEP ligand and two chlorides in a distorted tetrahedral geometry (Fig. 2, left). The ligand shows little delocalization beyond the aromatic rings themselves, with clear distinction in bond length between the central double bond (C6–C12, 1.333(3) Å) and the single bonds connecting C6 and C12 to the pyridine and phenyl rings respectively (1.493(3)–1.509(3) Å). This is consistent with the rotation of the phenyl rings out of the plane of the central carbon atoms. As noted in the introduction, the phenyl rings are angled towards the metal centre, with an angle between the central double bond (defined by the average plane through C5, C6, C7 and C12) and the plane defined by N1, Zn1 and N2 of 94.39(14)° (Fig. 2).

Complexation of ligand ^*t*Bu^PPBQ to ZnCl_2_ was also performed in refluxing EtOH, but with longer reaction times to cleanly achieve full conversion (Scheme 1). The product was purified by recrystallization from EtOH to afford [Zn(^*t*Bu^PPBQ)Cl_2_] in a 31% isolated yield. The compound a shows a red-shift in both the absorption and emission compared to the free ligand (Fig. S29 and 30). Crystals suitable for single-crystal X-ray structure determination were obtained from a solution in EtOH and reveal a similar distorted tetrahedral zinc centre as described for [Zn(^*t*Bu^PEP)Cl_2_] (Fig. 2, middle). Delocalization over the benzo[*f*]quinoline unit follows from the bond distances. The distance between the central two carbon bonds (C6–C12, 1.367(5) Å) is longer compared to the analogous distance in [Zn(^*t*Bu^PEP)Cl_2_] (C6–C12, 1.333(3) Å), and the distances C12–C13 (1.446(4) Å) and C5–C6 (1.452(4) Å) are shorter compared to the analogous distances in [Zn(^*t*Bu^PEP)Cl_2_] (C12–C13, 1.509(3) Å; C5–C6 1.493(3) Å). The planarity of the benzo[*f*]quinoline unit compared to the dipyridine ligand in [Zn(^*t*Bu^PEP)Cl_2_] also widened the angle between the central carbon–carbon bond (defined by the average plane through C6, C12, C13 and C19) and the plane defined by N1, Zn and N2 to 119.0(2)° (Fig. S2).

Both zinc complexes show the expected signals in the ^1^H and ^13^C NMR spectra in agreement with the X-ray crystal structures.

In [Zn(^*t*Bu^PPBQ)Cl_2_], signals belonging to the *tert*-butyl phenyl group are broadened, most likely due to restricted rotation (Fig. S21).

Nickel complexes were synthesised by refluxing NiCl_2_ and the ligand together in EtOH, analogous to the zinc complexes (Fig. 2). In this way, complex [Ni(^*t*Bu^PEP)_2_Cl_2_] was obtained in a 65% isolated yield after recrystallization from EtOH. Crystals suitable for single-crystal X-ray structure determination were grown from dichloromethane/petroleum ether (DCM/PE) (Fig. 2). Whereas the zinc complexes described previously feature one ^*t*Bu^PEP ligand, the nickel complex coordinates two ^*t*Bu^PEP ligands and two axial chlorides, resulting in a distorted octahedral geometry with inversion symmetry around Ni. Bond lengths in the ligand are similar to those in [Zn(^*t*Bu^PEP)Cl_2_], but the angle between the central double bond (defined by the average plane through C5, C6, C7 and C12) and the plane defined by N1, Ni1 and N2 is 110.04(12)° (Fig. 2), larger than the analogous angle in [Zn(^*t*Bu^PEP)Cl_2_], most likely due to the more crowded octahedral coordination.

[Ni(^*t*Bu^PPBQ)_2_Cl_2_] was synthesized according to the same protocol and obtained in a 43% isolated yield after recrystallization from an ethanol/petroleum ether mixture with a drop of acetone. Unfortunately, no single crystals of sufficient quality for single-crystal X-ray structure determination were obtained. However, ESI-MS showed the mass corresponding to [Ni(^*t*Bu^PPBQ)_2_Cl]^+^, suggesting that the complex consists of two ^*t*Bu^PPBQ ligands and two chloride ions (Fig. S56). Both nickel complexes are paramagnetic as expected for octahedral nickel(ii), with broadened ^1^H NMR signals between 0 and 60 ppm. While the high signal broadening and paramagenetic shifts prevents assignment of the ^1^H NMR signals, [Ni(^*t*Bu^PPBQ)_2_Cl_2_] clearly shows more signals in the ^1^H NMR spectrum than [Ni(^*t*Bu^PEP)_2_Cl_2_], as expected for the asymmetric ligand.

Interestingly, Wagner *et al.* describe that coordination of their structurally similar dipyridine-based ligand to PdCl_2_ prevents the analogous photochemical cyclodehydrogenation reaction. In our case, however, the outcome of the photochemical cyclodehydrogenation proceeds unchanged after coordination to ZnCl_2_. Unfortunately, no desired templating effect is observed either, and no products from the double Mallory reaction were observed.

### Electrochemical characterization

To investigate the redox properties of all four complexes, cyclic voltammetry (CV) studies were performed. The redox potentials for all four complexes in both tetrahydrofuran (THF) and DCM solvent are summarised in Table 1.

**Table 1:** Potentials of the reductive events of [Zn(^*t*Bu^PEP)Cl_2_], [Zn(^*t*Bu^PPBQ)Cl_2_], [Ni(^*t*Bu^PEP)_2_Cl_2_] and [Ni(^*t*Bu^PPBQ)_2_Cl_2_] measured by CV in both DCM and THF

	Reduction potential^a^ (V *vs.* Fc/Fc^+^) in THF/DCM		
[Zn(^*t*Bu^PEP)Cl_2_]	n.a./n.a.	−2.15/−2.16	−2.65/n.a.
[Zn(^*t*Bu^PPBQ)Cl_2_]	n.a./n.a.	−1.83/−2.07	−2.69/n.a.
[Ni(^*t*Bu^PEP)_2_Cl_2_]	−1.60/−1.7^c^	−2.21/−2.1^b^	−2.66/n.a.
[Ni(^*t*Bu^PPBQ)_2_Cl_2_]	−1.59/−1.5^c^	−2.02/−1.8^b^	−2.85/n.a.

a Unless stated otherwise, potentials are peak potentials (*E*_p_).

b Onset potential.

c Approximate potential where the current plateau is first reached.

**Fig. 3:**
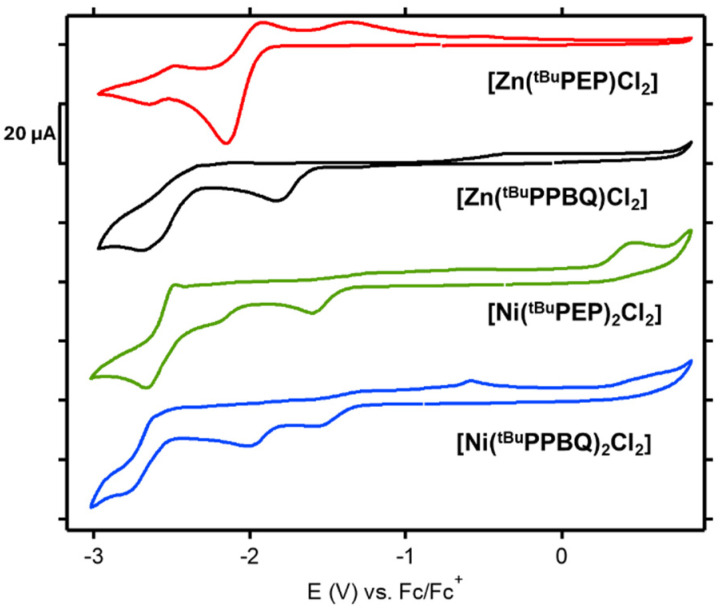
CV traces of [Zn(^*t*Bu^PEP)Cl_2_], [Zn(^*t*Bu^PPBQ)Cl_2_], [Ni(^*t*Bu^PEP)_2_Cl_2_] and [Ni(^*t*Bu^PPBQ)_2_Cl_2_]. Measurements were performed in THF (1 mM analyte), 0.2 M N^*n*^Bu_4_PF_6_ using a glassy carbon WE, a Pt wire CE and a Ag/AgNO_3_ (saturated solution in the electrolyte) RE.

The CV traces of all four complexes measured in THF are shown in Fig. 3. [Zn(^*t*Bu^PEP)Cl_2_] shows two reductive events, with peak potentials of −2.15 V *vs.* Fc/Fc^+^ and −2.65 V *vs.* Fc/Fc^+^. [Zn(^*t*Bu^PPBQ)Cl_2_] similarly shows two reductive events with peak potentials at −1.83 V *vs.* Fc/Fc^+^ and −2.69 V *vs.* Fc/Fc^+^. [Ni(^*t*Bu^PEP)_2_Cl_2_] and [Ni(^*t*Bu^PPBQ)_2_Cl_2_] both show three irreversible reduction events, with peak potentials of −1.60, −2.21 and −2.66 V *vs.* Fc/Fc^+^ for [Ni(^*t*Bu^PEP)_2_Cl_2_] and −1.59, −2.02 and −2.85 V *vs.* Fc/Fc^+^ for [Ni(^*t*Bu^PPBQ)_2_Cl_2_]. Although from these analyses it is not entirely clear what reduction events are either metal- or ligand centred, at least one event seems to be ligand centred with all complexes, which is supported by the CV of ^*t*Bu^PEP in THF which features an irreversible reduction event with a peak potential of −2.72 V *vs.* Fc/Fc^+^ (Fig. S31a). In addition, for the ^*t*Bu^PPBQ complexes, the reductions are generally shifted towards less negative potentials, with the exception of the last reduction, compared to ^*t*Bu^PEP as expected for the larger aromatic π-system.

**Fig. 4:**
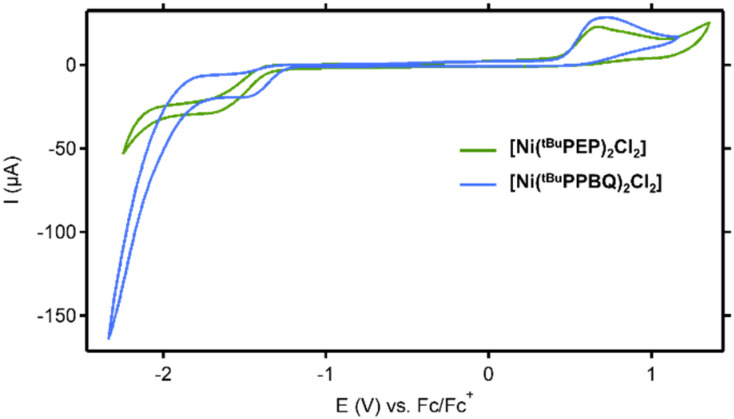
CV traces of [Ni(^*t*Bu^PEP)_2_Cl_2_] and [Ni(^*t*Bu^PPBQ)_2_Cl_2_]. Measurement was performed in DCM (1 mM analyte), 0.1 M N^n^Bu_4_PF_6_ using a glassy carbon WE, a Pt wire CE and a Ag/AgNO_3_ (saturated solution in the electrolyte) RE.

Switching to DCM electrolyte, the results are markedly different. For the zinc complexes, one minor redox event is observed within the potential window, around −2 V *vs.* Fc/Fc^+^ (Fig. S32). The low currents compared to the events in the analogous nickel complexes or the same complexes measured in THF (*vide infra*) suggest that these couples are due to a minor impurity or decomposition product. In contrast, both nickel complexes show clear redox features, with an irreversible oxidation at 0.7 V *vs.* Fc/Fc^+^, as well as two irreversible reductive events (Fig. 4). The first of these events reaches what is best described as a current plateau at −1.7 V *vs.* Fc/Fc^+^ for [Ni(^*t*Bu^PEP)_2_Cl_2_] and −1.5 V *vs.* Fc/Fc^+^ for [Ni(^*t*Bu^PPBQ)_2_Cl_2_]. The second reductive event has an onset potential of −2.1 V *vs.* Fc/Fc^+^ for [Ni(^*t*Bu^PEP)_2_Cl_2_] and −1.8 V *vs.* Fc/Fc^+^ for [Ni(^*t*Bu^PPBQ)_2_Cl_2_]. Interestingly, for [Ni(^*t*Bu^PPBQ)_2_Cl_2_], currents increase significantly for the last reduction to ∼150 µA at −2.3 V *vs.* Fc/Fc^+^, more than five times higher than the current for the first reduction event for example. It is also noted that the measured potentials for both reductions are lower for [Ni(^*t*Bu^PPBQ)_2_Cl_2_] than for [Ni(^*t*Bu^PEP)_2_Cl_2_] which might be in-part due to the larger conjugated π-system of the former.

Intrigued by the plateau-shape and high currents in these voltammograms, we investigated the reactivity of our complexes with DCM and other organic halides in THF under reducing potentials. Encouragingly, catalysis was observed in the case of DCM for both nickel complexes. However, the small substrate scope, low activity and catalyst decomposition prevented us from isolating reaction products and drawing robust conclusions on the mechanism at this time. These preliminary findings are further discussed in the SI (section S5).

## Conclusions

We have reported on the synthesis of two new ligands and their corresponding complexes of zinc and nickel. The dipyridine ligand could undergo a photochemical Mallory reaction to be converted to the pyridinyl benzoquinoline ligand, but a second cyclodehydrogenation reaction to form a fully-fused quinolinoquinoline ligand was unsuccessful even after templating with various (Lewis) acids or metals. The nickel and zinc complexes were fully characterized, showing that the zinc complexes adopt a tetrahedral geometry, while the nickel complexes are octahedral. Further electrochemical characterisations revealed multiple, irreversible ligand- and metal-based reductions in THF-based electrolyte, while in DCM-based electrolyte, catalytic reactivity at low potentials with the solvent was observed with both nickel complexes. Together, this work adds to the limited number of dipyridine-type complexes reported to date, demonstrating their synthesis and properties.

## Conflicts of interest

There are no conflicts to declare.

## Supplementary Material

DT-055-D6DT00659K-s001

DT-055-D6DT00659K-s002

## Data Availability

Supplementary information (SI): experimental procedures, characterisation, spectroscopic data and electrochemical data. See DOI: https://doi.org/10.1039/d6dt00659k. Spectroscopic and electrochemical data files that support the findings of this study are openly available in the Yoda data repository at https://doi.org/10.24416/UU01-HWZJXV. CCDC 2495948–2495950 contain the supplementary crystallographic data for this paper.^22*a*–*c*^
