# Supplementary material for: Synthesis and characterisation of novel dipyridine and pyridinyl benzoquinoline complexes of zinc and nickel
Source: Dalton Trans. 2026 May 26;55(24):9265–70. doi: 10.1039/d6dt00659k (PMC13245842; doi:10.1039/d6dt00659k)
Supplement: DT-055-D6DT00659K-s001 [file DT-055-D6DT00659K-s001.pdf]

Supplementary Information (SI) for Dalton Transactions.
This journal is © The Royal Society of Chemistry 2026

Supplementary Information

Synthesis and Characterisation of Novel Dipyridine and Pyridinyl Benzoquinoline Complexes of Zinc and Nickel

Lars Killian[a], Anne Olarte Loyo[a,b], Roy R.P.A.M. de Ridder[a], Martin Lutz[c], Arnaud Thevenon*[a]

[a] Organic Chemistry and Catalysis, Institute for Sustainable and Circular Chemistry, Faculty of Science, Utrecht University, Universiteitsweg 99, 3584 CG, Utrecht (The Netherlands)

[b] Current address: Department of Organic and Inorganic Chemistry, Faculty of Science and Technology, University of the Basque Country (UPV/EHU), P.O. Box 644, 48080 Bilbao (Spain)

[c] Structural Biochemistry, Bijvoet Centre for Biomolecular Research, Faculty of Science, Utrecht University, Universiteitsweg 99, 3584 CG, Utrecht (The Netherlands)

S1. General remarks 2
S2. Synthesis and characterization 3
2,2'-(2,2-dibromoethene-1,1-diyl)dipyridine (BEP) 3
2,2'-(2,2-bis(4-(*tert*-butyl)phenyl)ethene-1,1-diyl)dipyridine (^{tBu}PEP) 4
9-(*tert*-butyl)-6-(4-(*tert*-butyl)phenyl)-5-(pyridin-2-yl)benzo[*f*]quinoline (^{tBu}PPBQ) 5
[Zn(^{tBu}PEP)Cl_2] 6
[Zn(^{tBu}PPBQ)Cl_2] 7
[Ni(^{tBu}PEP)$_2Cl_2$] 8
[Ni(^{tBu}PPBQ)$_2Cl_2$] 9
S3. NMR spectra 10
2,2'-(2,2-dibromoethene-1,1-diyl)dipyridine (BEP) 10
2,2'-(2,2-bis(4-(tert-butyl)phenyl)ethene-1,1-diyl)dipyridine (^{tBu}PEP) 12
9-(*tert*-butyl)-6-(4-(*tert*-butyl)phenyl)-5-(pyridin-2-yl)benzo[*f*]quinoline (^{tBu}PPBQ) 15
[Zn(^{tBu}PPBQ)Cl_2] 20
[Ni(^{tBu}PEP)$_2Cl_2$] 22
[Ni(^{tBu}PPBQ)$_2Cl_2$] 23
Catalyst decomposition experiment 23
S4. UV-VIS 25
S5. Electrochemistry 26
S6. X-ray crystal structure determinations 34
[Zn(^{tBu}PEP)Cl_2] 34
[Zn(^{tBu}PPBQ)Cl_2] 36
[Ni(^{tBu}PEP)$_2Cl_2$] 38
S7. IR spectra 40
S8. HRMS spectra 43
References 44

S1. General remarks

Where necessary, manipulations were performed under inert N_2 atmosphere using standard Schlenk technique or in a N_2-filled M. Braun Glovebox. Glassware was dried at 130 °C over-night or flame-dried under dynamic vacuum. Unless otherwise stated, commercial reagents and solvents were used as received. Substrates for the dehalogenation experiments were degassed by sparging with N_2 and stored over 4 Å molecular sieves. Dichloromethane was collected from an M. Braun MB-SPS 800 solvent purification system and stored on 3 Å molecular sieves. THF was distilled over sodium/benzophenone or over Na on inorganic supporter (SOLVONA®), vacuum-transferred, degassed and stored on 4 Å molecular sieves.

NMR measurements were performed at 25 °C on a Varian VNMRS400, a Varian MRF400 or a Jeol JNM-ECZL G 400 MHz spectrometer. Chemical shifts in ^{1}H and ^{13}C are reported relative to TMS with the residual solvent signal[1] as internal standard where the influence of analytes on the solvent can be assumed negligible. All NMR experiments involving air-sensitive compounds were conducted in J. Young NMR tubes under an N_2 atmosphere. Peak multiplicity is quoted as s (singlet), bs (broad singlet), d (doublet), t (triplet) and so on.

FT-IR data was recorded on a PerkinElmer SpectrumTwo Infrared Spectrophotometer with an ATR probe.

ESI-MS data was recorded on an Advion Expression L cms equipped with a TLC plate reader (Plate Express) using either MeCN as carrier solvents. HRMS was measured on an Agilent Technologies 6560 ion mobility QTOF using direct infusion.

UV-VIS data was recorded on a PerkinElmer Lambda950. Fluorescence emission data was recorded on a JASCO FP-8300.

Electrochemical measurements were performed in a N_2-filled MBraun labmaster dp glovebox, using an IVIUM potentiostat/galvanostat. A three-electrode set-up was used with $Ag/AgNO_3$ (saturated in the electrolyte) reference electrode, Pt wire counter electrode and glassy carbon (3 mm Ø) working electrode directly in solution. All electrochemical data is referenced to the ferrocene/ferrocenium couple, measured on the same day as the experiment or referenced to previous measurements of the same compound. For the CPE measurements, a custom H-cell set-up was used with ~3 mL catholyte and anolyte compartments separated by a P5 glass frit.

Elemental analysis was performed by MEDAC ltd. in the United Kingdom.

Spectroscopic and electrochemical data files that support the findings of this study are openly available in the Yoda data repository at: https://doi.org/10.24416/UU01-HWZJXV.

S2. Synthesis and characterization

2,2'-(2,2-dibromoethene-1,1-diyl)dipyridine (BEP)

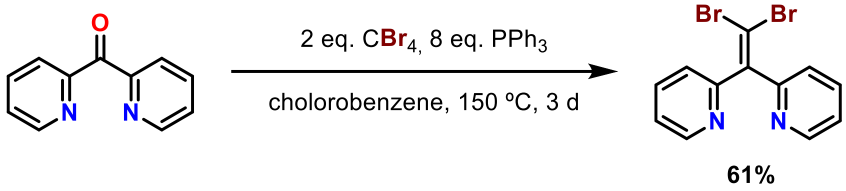

The synthesis was performed according to the literature.[2] Di(2-pyridyl) ketone (3.01 g, 16.3 mmol, 1 eq.) was dissolved in chlorobenzene (400 mL) in a Schlenk under N_2 atmosphere. Carbon tetrabromide (10.9 g, 32.7 mmol, 2 eq.) and PPh_3 (34.3 g, 130.6 mmol, 8 eq.) were added and the reaction mixture was heated to reflux. After 3 days, the reaction mixture was allowed to cool to room temperature and insoluble material was removed by filtration. The filtrate was treated with HCl (100 mL, 4 M aq.). The aqueous layer was separated and basified with NaOH (1 M aq.) until pH 12. The aqueous phase was extracted with DCM (3 × 400 mL), and the combined organic fractions were washed with water (2 × 200 mL) and brine (2 × 200 mL), were dried over anhydrous $MgSO_4$ and concentrated under reduced pressure. The crude product was purified column chromatography on silica gel (1/1 PE/EtOAc) followed by column chromatography on basic alumina (1/1 PE/EtOAc) to yield 2,2'-(2,2-dibromoethene-1,1-diyl)dipyridine (**BEP**) (3.4 g, 61 %) as off-white solid. The NMR data are consistent with the literature.[2]

^{1}H NMR (400 MHz, $CDCl_3$, 298 K): δ = 8.64 – 8.57 (m, 2H), 7.73 (apparent td, $^3J_{H,H}$ = 7.7, $^4J_{H,H}$ 1.8 Hz, 2H), 7.59 (d, $^3J_{H,H}$ = 7.9 Hz, 2H), 7.22 (ddd, $^3J_{H,H}$ = 7.6, $^3J_{H,H}$ = 4.8, $^4J_{H,H}$ = 1.2 Hz, 2H) ppm.

^{13}C NMR (101 MHz, $CDCl_3$, 298 K): δ = 158.1, 149.7, 146.6, 136.7, 124.9, 123.0, 95.8 ppm.

2,2'-(2,2-bis(4-(*tert*-butyl)phenyl)ethene-1,1-diyl)dipyridine (^{tBu}PEP)

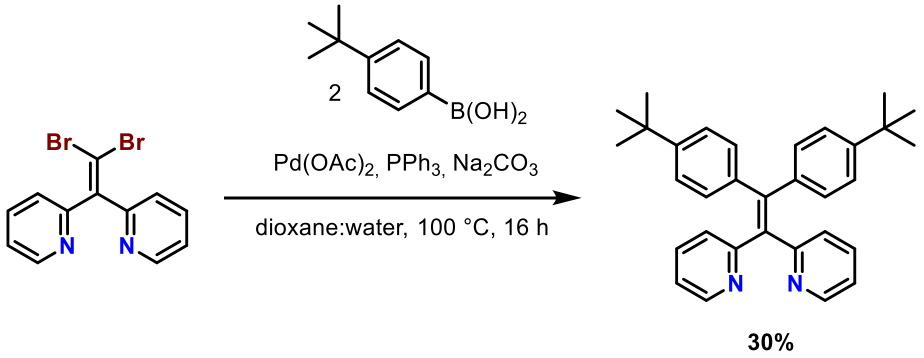

The procedure was adapted from the literature.[2] **BEP** (2.782 g, 8.18 mmol) was dissolved in degassed dioxane:water (4:1, 410 mL) in a Schlenk tube under N_2 atmosphere. After that, Na_2CO_3 (4.336 g, 40.91 mmol, 5 eq.), $Pd(OAc)_2$ (0.220 g, 0.98 mmol, 0.12 eq.), PPh_3 (1.073 g, 4.09 mmol, 0.5 eq.) and 4-*tert*-butyl phenylboronic acid (7.284 g, 40.91 mmol, 5 eq.) were added. The reaction was heated to reflux under N_2 overnight. After cooling down to room temperature, the reaction mixture was diluted with water (200 mL) and extracted with EtOAc (3 × 400 mL). The combined organic fractions were washed with water (2 × 400 mL) and brine (2 × 400 mL) and dried over anhydrous $MgSO_4$ and concentrated under reduced pressure. The crude product was purified by column chromatography on basic alumina using a gradient eluent (1/0 → 4/1 PE/EtOAc) followed by column chromatography on silica using a gradient eluent (1/1 → 1/2 → 0/1 PE/EtOAc) to yield 2,2'-(2,2-bis(4-(*tert*-butyl)phenyl)ethene-1,1-diyl)dipyridine (**^{tBu}PEP**) (1.059 g, 30 %) as a white solid.

^{1}H NMR (400 MHz, $CDCl_3$, 298 K): δ = 8.50 (ddd, $^3J_{H,H}$ = 4.9, $^4J_{H,H}$ = 1.8, $^5J_{H,H}$ = 0.9 Hz, 2H), 7.35 (d, $^3J_{H,H}$ = 1.9 Hz, 2H), 7.14 – 7.10 (m, 4H), 7.05 (apparent dt, $^3J_{H,H}$ = 7.9, $^4J_{H,H}$ 1.1 Hz, 2H), 7.01 – 6.97 (m, 2H), 6.97 – 6.94 (m, 4H), 1.25 (s, 18H, CH_3) ppm.

^{13}C NMR (101 MHz, $CDCl_3$, 298 K): δ (ppm) = 161.9, 150.4, 149.3, 145.3, 139.9, 139.6, 135.7, 130.9, 127.1, 124.7, 121.2, 34.6, 31.4

ATR-IR: ν = 3049 (w), 2962 (s), 2903 (m), 2865 (m), 1591 (w), 1584 (m), 1563 (m), 1509 (m), 1465 (m), 1428 (m), 1393 (w), 1392 (w), 1362 (m), 1328 (w), 1291 (w), 1274 (w), 1268 (m), 1241 (w), 1202 (w), 1148 (w), 1105 (w), 1092 (w), 1027 (w), 999 (w), 997 (w), 867 (w), 841 (m), 780 (m), 744 (m), 699 (w), 664 (m), 637 (w), 590 (w), 584 (m), 547 (m) cm^{-1}.

Anal. Calcd. for $C_{32}H_{34}N_2$: C, 86.06; H, 7.67; N, 6.27 %. Found: C, 85.97; H, 8.00; N, 6.26 %.

HR-MS: m/z = 447.28081 {$[M+H]^+$, calc. 447.28002}.

9-(*tert*-butyl)-6-(4-(*tert*-butyl)phenyl)-5-(pyridin-2-yl)benzo[*f*]quinoline (^{tBu}PPBQ)

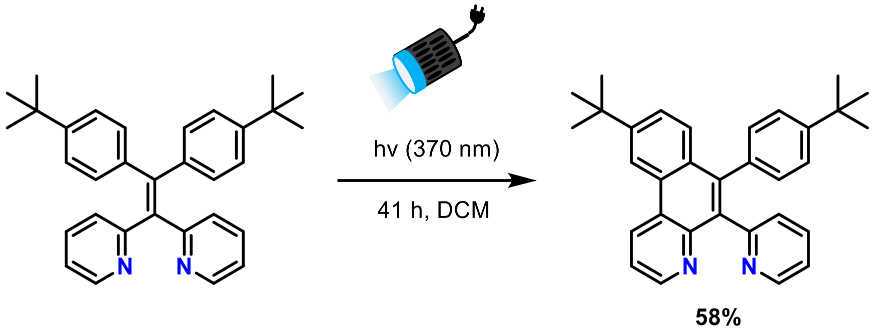

^{tBu}PEP (252 mg, 0.564 mmol) was dissolved in DCM (22.5 mL) in a 100 mL round bottom flask. The solution was irradiated with a 370 nm lamp (see note) at room temperature while stirring for 41 hours. The crude product was purified by column chromatography on silica (1/1 PE/EtOAc) to yield 9-(*tert*-butyl)-6-(4-(*tert*-butyl)phenyl)-5-(pyridin-2-yl)benzo[*f*]quinoline **(^{tBu}PPBQ)** as an orangish powder (146 mg, 58 %).

^{1}H NMR (400 MHz, CDCl$_3$, 298 K): δ = 9.08 (dd, $^3J_{H,H}$ = 8.6, $^4J_{H,H}$ = 1.7 Hz, 1H), 8.91 (dd, $^3J_{H,H}$ = 4.3, $^4J_{H,H}$ = 1.6 Hz, 1H), 8.71 (d, $^3J_{H,H}$ = 1.8 Hz, 1H), 8.61 (ddd, $^3J_{H,H}$ = 4.9, $^4J_{H,H}$ 1.8, $^5J_{H,H}$ = 1.0 Hz, 1H), 7.70 – 7.66 (m, 1H), 7.64 (dd, $^3J_{H,H}$ = 8.7, $^4J_{H,H}$ = 1.8 Hz, 1H), 7.55 (dd, $^3J_{H,H}$ = 8.4, $^4J_{H,H}$ = 4.3 Hz, 1H), 7.48 (apparent td, $^3J_{H,H}$ = 7.7, $^4J_{H,H}$ = 1.8 Hz, 1H), 7.24 (s, 2H), 7.15 – 7.04 (m, 4H), 1.50 (s, 9H), 1.29 (s, 9H) ppm.

^{13}C NMR (101 MHz, CDCl$_3$, 298 K): δ = 158.9, 150.1, 149.9, 149.7, 148.7, 147.6, 141.6, 136.9, 135.5, 135.3, 130.9, 130.5, 129.7, 129.7, 128.1, 126.5, 125.7, 125.4, 124.5, 121.2, 120.9, 118.2, 35.4, 34.6, 31.6, 31.5 ppm.

ATR-IR: ν = 2961 (s), 2926 (s), 2864 (m), 1585 (m), 1565 (m), 1518 (w), 1474 (w), 1469 (m), 1406 (m), 1404 (m), 1395 (w), 1362 (m), 1326 (w), 1263 (m), 1241 (w), 1205 (w), 1177 (w), 1148 (w), 1091 (m), 1034 (m), 1020 (m), 994 (w), 909 (w), 883 (w), 841 (m), 824 (m), 790 (w), 784 (m), 772 (m), 748 (m), 720 (w), 697 (w), 671 (w), 645 (w), 613 (w), 580 (w), 565 (w), 564 (w), 540 (w), 502 (w) cm^{-1}.

HR-MS: m/z = 445.26651 {[M+H]$^+$, calc. 445.26437}.

Note: The photochemical set-up consisted of a Kessil UVA LED PR160L-370 nm lamp (43 W) used at 100 % intensity and at a 5 cm from the sample

[Zn(^{tBu}PEP)Cl$_2$]

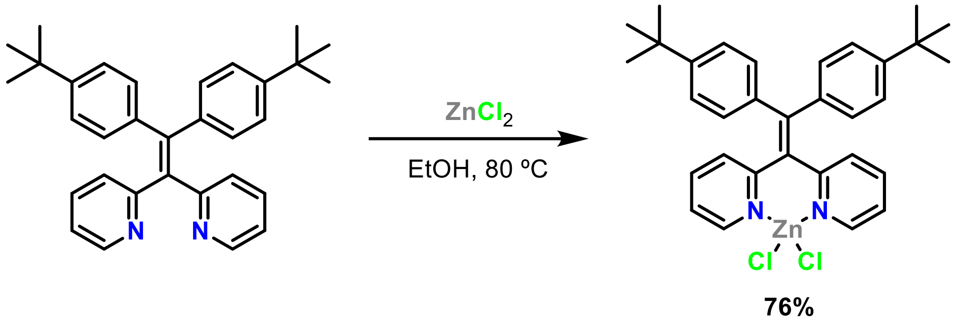

A suspension of ^{tBu}**PEP** (75.5 mg, 0.169 mmol) in ethanol (2 mL) was heated to reflux. Once a clear solution was obtained, a solution of $ZnCl_2$ (23.1 mg, 0.169 mmol) in ethanol (1 mL) was added dropwise. Immediately, a white precipitate formed which was isolated and recrystallized from ethanol to obtain **[Zn(^{tBu}PEP)Cl$_2$]** as a white powder (74.8 mg, 76 %). White needle crystals suitable for single-crystal XRD were grown from a solution in DCM by vapour diffusion with PE as antisolvent at room temperature.

^{1}H NMR (400 MHz, CDCl$_3$, 298 K): δ = 8.82 (ddd, $^3J_{H,H}$ = 5.4, $^4J_{H,H}$ 1.8, $^5J_{H,H}$ 0.9 Hz, 2H), 7.62 – 7.57 (m, 2H), 7.41 – 7.37 (m, 4H), 7.33 (ddd, $^3J_{H,H}$ = 7.7, $^3J_{H,H}$ 5.3, $^4J_{H,H}$ 1.3 Hz, 2H), 7.28 (apparent t (overlap with solvent signal), $^4J_{H,H}$ = 1.1 Hz, 2H), 7.23 – 7.19 (m, 4H), 1.21 (s, 18H) ppm.

^{13}C NMR (101 MHz, CDCl$_3$, 298 K): δ = 155.9, 153.2, 151.0, 148.6, 139.3, 136.4, 132.0, 128.4, 127.8, 125.4, 123.7, 34.6, 31.3 ppm.

IR–ATR (solid): ν = 3068 (w), 2956 (s), 2900 (s), 1633 (m), 1601 (s), 1567 (m), 1508 (m), 1474 (s), 1439 (s), 1406 (w), 1394 (m), 1361 (m), 1326 (w), 1282 (w), 1268 (m), 1243 (m), 1204 (w), 1158 (w), 1099 (m), 1098 (m), 1068 (m), 1058 (s), 1027 (s), 907 (w), 869 (m), 828 (m), 789 (s), 771 (m), 765 (s), 669 (m), 653, 632 (m), 590 (m), 578 (w), 560 (w), 487 (w) cm^{-1}.

Anal. Calcd. for $C_{32}H_{34}Cl_2N_2Zn$: C, 65.94; H, 5.88; N, 4.80 %. Found: C, 65.71; H, 5.77; N, 4.73 %.

[Zn(^{tBu}PPBQ)Cl_2]

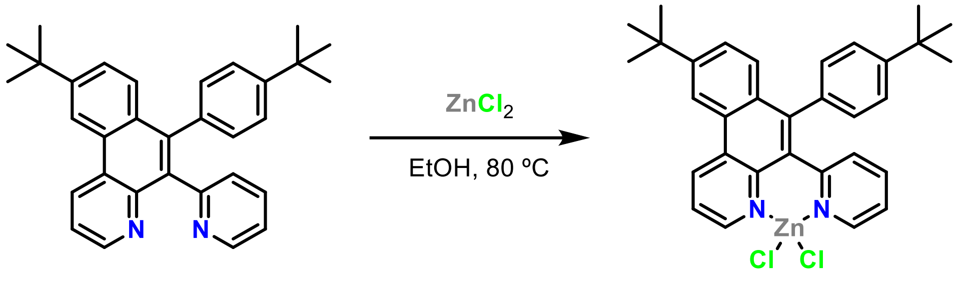

A suspension of **^{tBu}PPBQ** (75.1 mg, 0.17 mmol) in ethanol (3 mL) was heated to reflux. Once a clear yellowish solution was obtained, a colourless solution of $ZnCl_2$ (23.5, 0.17 mmol) in ethanol (1.5 ml) was added dropwise. The mixture was stirred for 2 hours at reflux. Once a precipitate had formed, the resulting suspension was filtered and recrystallized from ethanol several times to obtain **[Zn(^{tBu}PPBQ)Cl_2]** as white solid (30 mg, 31 %). White, needle-shaped crystals suitable for single-crystal XRD were grown from EtOH at room temperature.

^{1}H NMR (400 MHz, $CDCl_3$, 298 K): δ = 9.35 (dd, $^3J_{H,H}$ = 8.6, $^4J_{H,H}$ 1.6 Hz, 1H), 9.10 (dd, $^3J_{H,H}$ = 4.7, $^4J_{H,H}$ = 1.5 Hz, 1H), 8.93 – 8.89 (m, 1H), 8.71 (d, $^4J_{H,H}$ = 1.9 Hz, 1H), 8.00 (d, $^3J_{H,H}$ = 8.8 Hz, 1H), 7.87 (dd, $^3J_{H,H}$ = 8.4, $^3J_{H,H}$ = 4.7 Hz, 1H), 7.79 (dd, $^3J_{H,H}$ = 8.8, $^4J_{H,H}$ = 1.9 Hz, 1H), 7.6 – 7.1 (bs, 2H) 7.42 (apparent td, $^3J_{H,H}$ = 7.9, $^4J_{H,H}$ = 1.9 Hz, 1H), 7.35 – 7.28 (m, 1H), 6.96 (d, $^3J_{H,H}$ = 8.1 Hz, 1H), 1.52 (s, 9H), 1.32 (s, 9H) ppm.

^{13}C NMR (101 MHz, $CDCl_3$, 298 K): δ = 156.3, 153.2, 152.3, 149.6, 148.5, 148.3, 145.0, 138.3, 135.1, 135.0, 132.0, 130.1, 129.6, 129.4, 127.4, 126.6, 126.5, 123.1, 122.4, 118.5, 35.7, 34.9, 31.5, 31.4 ppm.

IR–ATR: ν = 3675 (m), 2971 (s), 2901 (s), 1604 (w),1579 (w), 1475 (w), 1398 (m), 1242 (w), 1066 (m), 794 (w), 583 (w) cm^{-1}.

Note: Because of the broadening of some signals in the ^{1}H NMR, and their seeming absence in the ^{13}C NMR, full assignment of all signals was not possible.

23

[Ni(^{tBu}PEP)$_2$Cl$_2$]

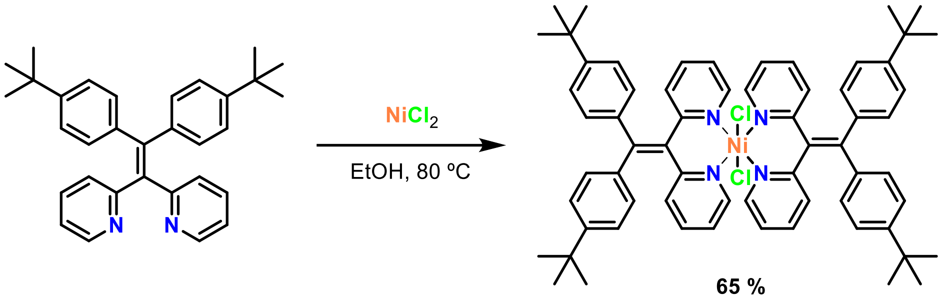

A suspension of ^{tBu}**PEP** (128.8 mg, 0.29 mmol, 2 eq.) in ethanol (4 mL) was heated to reflux. Once a clear solution was obtained, a yellowish suspension of $NiCl_2$ (18.6 mg, 0.14 mmol, 1 equiv.) in ethanol (2 mL) was added dropwise. After stirring at reflux for 4 hours, the solution was left to cool down to room temperature to give a blue-green solution. The solvent was evaporated under reduced pressure and the product was recrystallized from ethanol to obtain **[Ni(^{tBu}PEP)$_2$Cl$_2$]** as blue/green crystals (95.2 mg, 65 %). Green needle crystals suitable for single-crystal XRD were grown from DCM by vapour diffusion with PE as antisolvent at room temperature.

^{1}H NMR (400 MHz, $CDCl_3$, 298 K): δ = 56.48 (bs, 8H), 45.95 (bs, 4H), 38.40 (bs, 4H), 24.94 (bs, 4H), 15.41 (bs, 4H) ppm.

IR–ATR (solid): ν = 3057 (w), 2961 (m), 2906 (w), 2867 (w), 1622 (w), 1596 (m), 1568 (m), 1505 (m), 1478 (m), 1469 (m), 1425 (m), 1395 (w), 1361 (m), 1269 (m), 1204 (w), 1154 (m), 1110 (m), 1061 (m), 1020 (m), 870 (m), 844 (m), 824 (m), 792 (s), 764 (s), 713 (w), 669 (m), 648 (m), 630 (s), 589 (s), 563 (m), 497 (w) cm^{-1}.

[Ni(^{tBu}PPBQ)$_2$Cl$_2$]

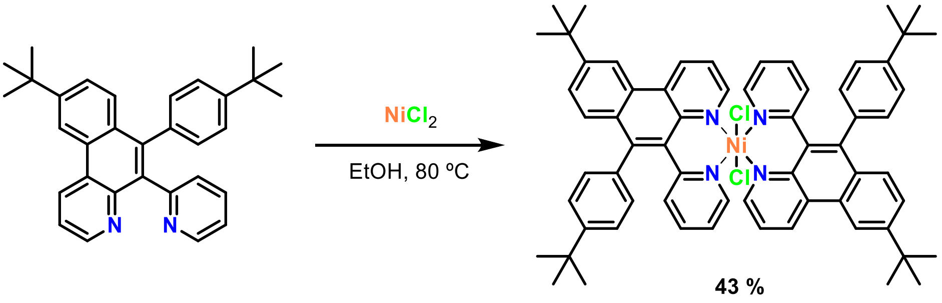

A suspension of **^{tBu}PPBQ** (72.6 mg, 0.16 mmol, 2 eq.) in ethanol (2 mL) was heated to reflux. Once a clear solution was obtained, a yellowish suspension of $NiCl_2$ (10.6 mg, 0.08 mmol, 1 eq.) in ethanol (2 mL) was added dropwise. After stirring at reflux for 21.5 h, the solution was left to cool down to room temperature to give a green-yellow solution. The solution was concentrated under reduced pressure and the product was recrystallized by layering a concentrated solution in ethanol with petroleum ether and a drop of acetone to obtain **[Ni(^{tBu}PPBQ)$_2$Cl$_2$]** as a green powder (35.9 mg, 43 %).

^{1}H NMR (400 MHz, $CDCl_3$, 298 K): δ = 52.22 (bs), 50.46 (bs), 46.89 (bs), 41.89 (bs), 39.28 (bs), 37.97 (bs), 35.02 (bs), 15.81 (s), 15.25 (s), 14.16 (s), 13.04 (s), 12.01 (s), 11.27 (s) ppm.

IR–ATR (solid): ν = 2958 (m), 1597 (m), 1475 (m), 1396 (m), 1363 (m), 1263 (m), 1236 (m), 1109 (m), 1021 (m), 913 (m), 854 (m), 836 (m), 823 (m), 788 (s), 770 (m), 752 (m), 672 (m), 632 (m), 585 (m), 562 (m) cm^{-1}.

ESI-MS: m/z = 981.4 {[M-Cl]+ , calc. 981.4}**.**

S3. NMR spectra

2,2'-(2,2-dibromoethene-1,1-diyl)dipyridine (BEP)

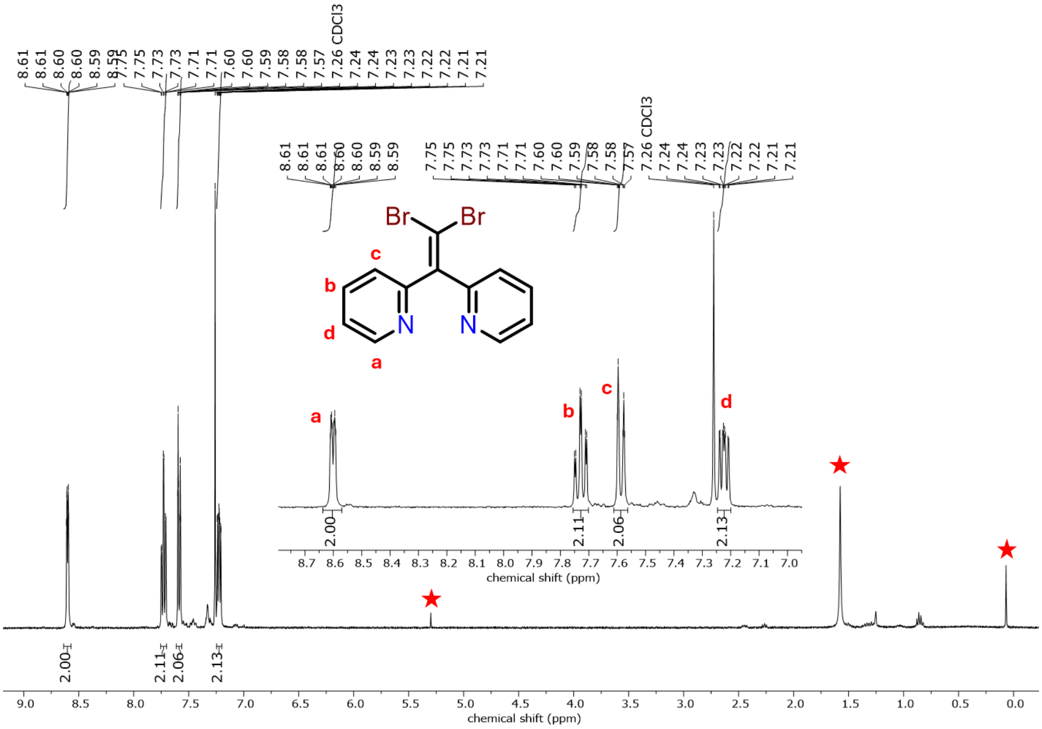

Figure S1: ^{1}H NMR spectrum of 2,2'-(2,2-dibromoethene-1,1-diyl)dipyridine **(BEP)** in $CDCl_3$. Resonances marked with a star are assigned to residual DCM (5.30 ppm), water (1.58 ppm) and grease (0.07 ppm).

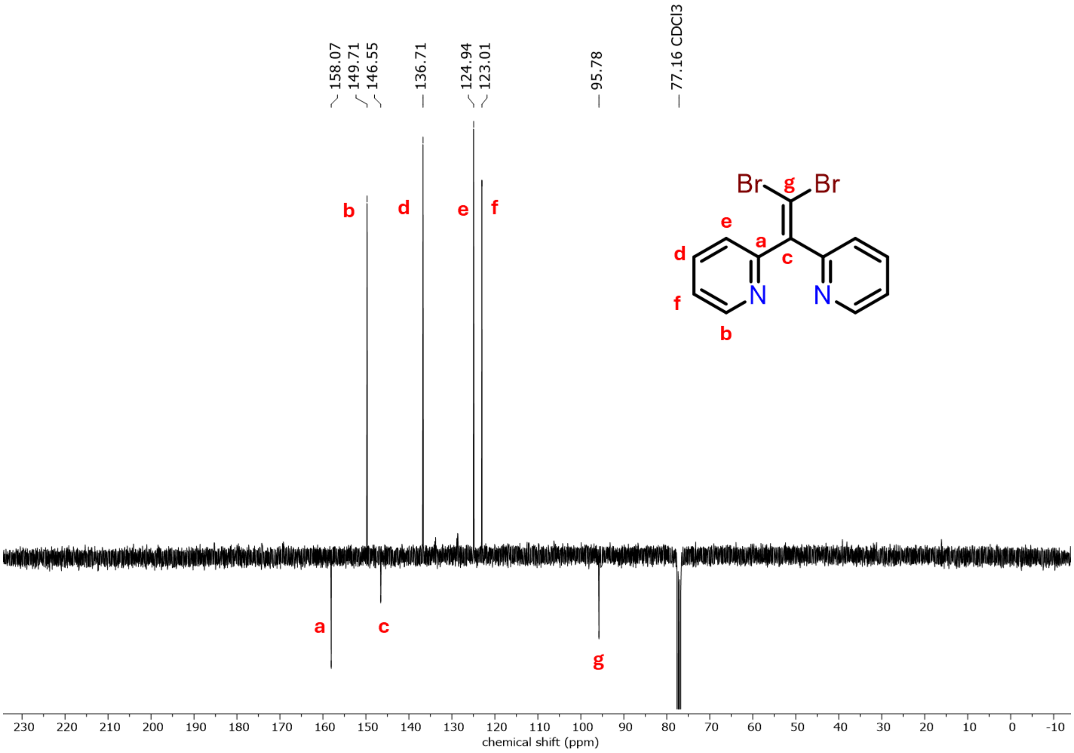

Figure S2: ^{13}C-APT NMR spectrum of 2,2'-(2,2-dibromoethene-1,1-diyl)dipyridine **(BEP)** in $CDCl_3$, at 25 °C.

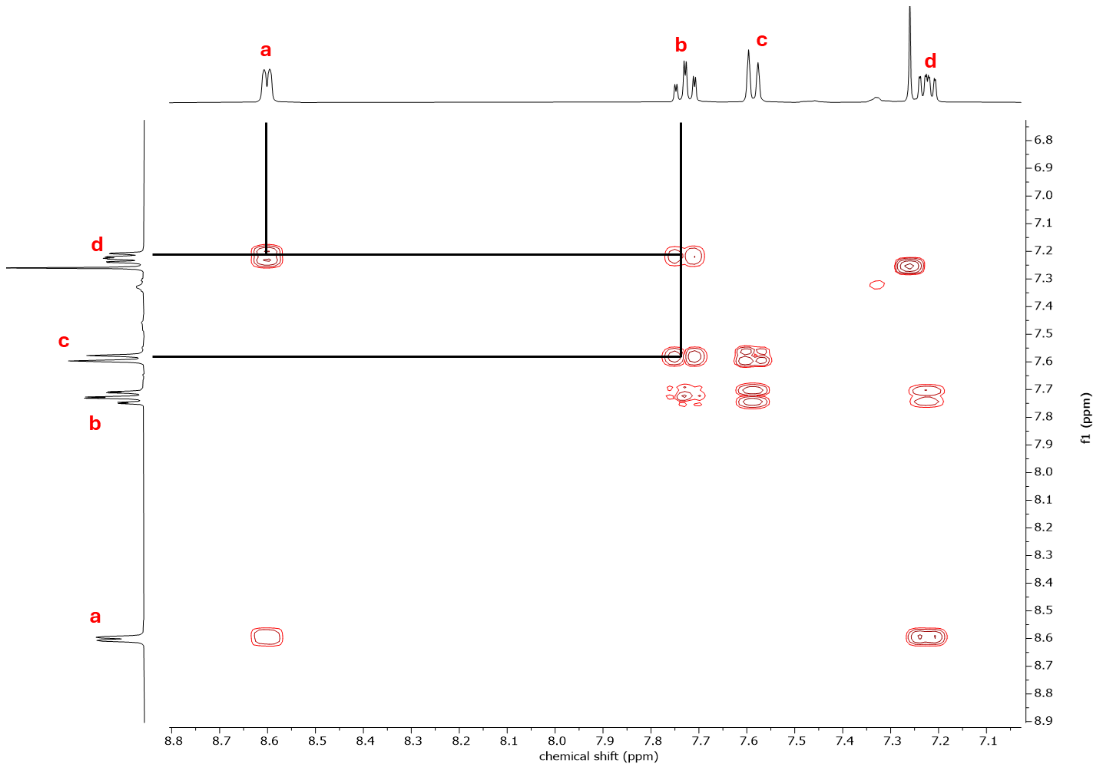

Figure S3: gCOSY NMR spectrum of 2,2'-(2,2-dibromoethene-1,1-diyl)dipyridine **(BEP)** in $CDCl_3$, at 25 °C.

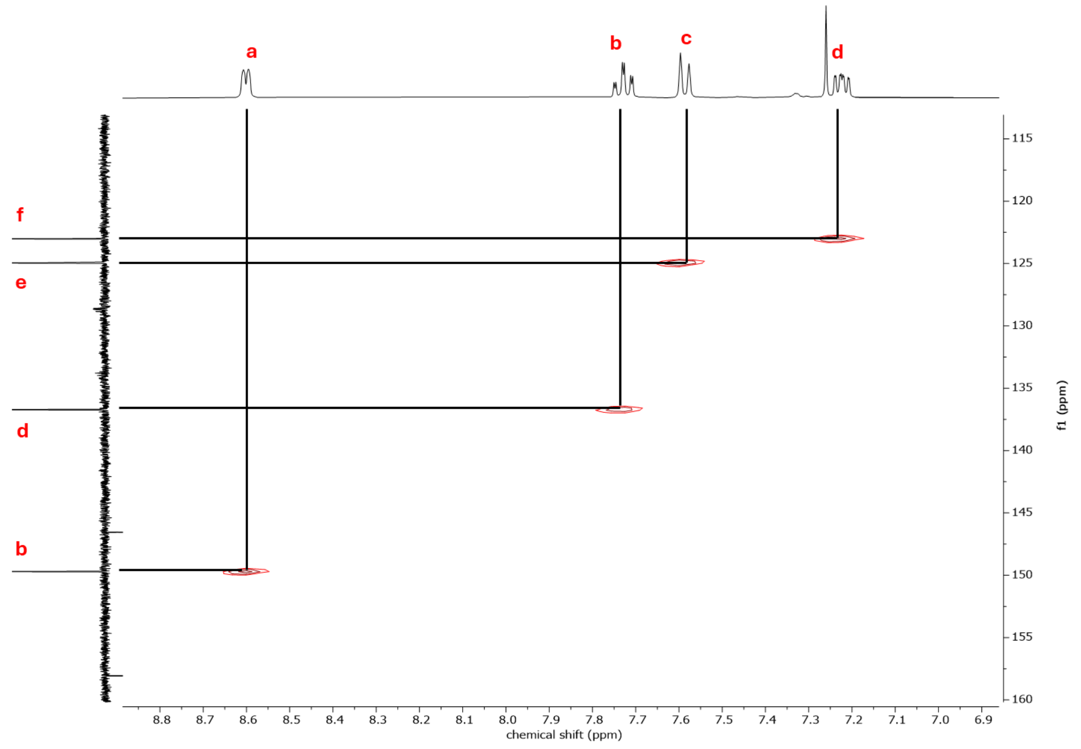

Figure S4: ^{1}H-^{13}C ASAP-HMQC NMR spectrum of 2,2'-(2,2-dibromoethene-1,1-diyl)dipyridine **(BEP)** in $CDCl_3$, at 25 °C.

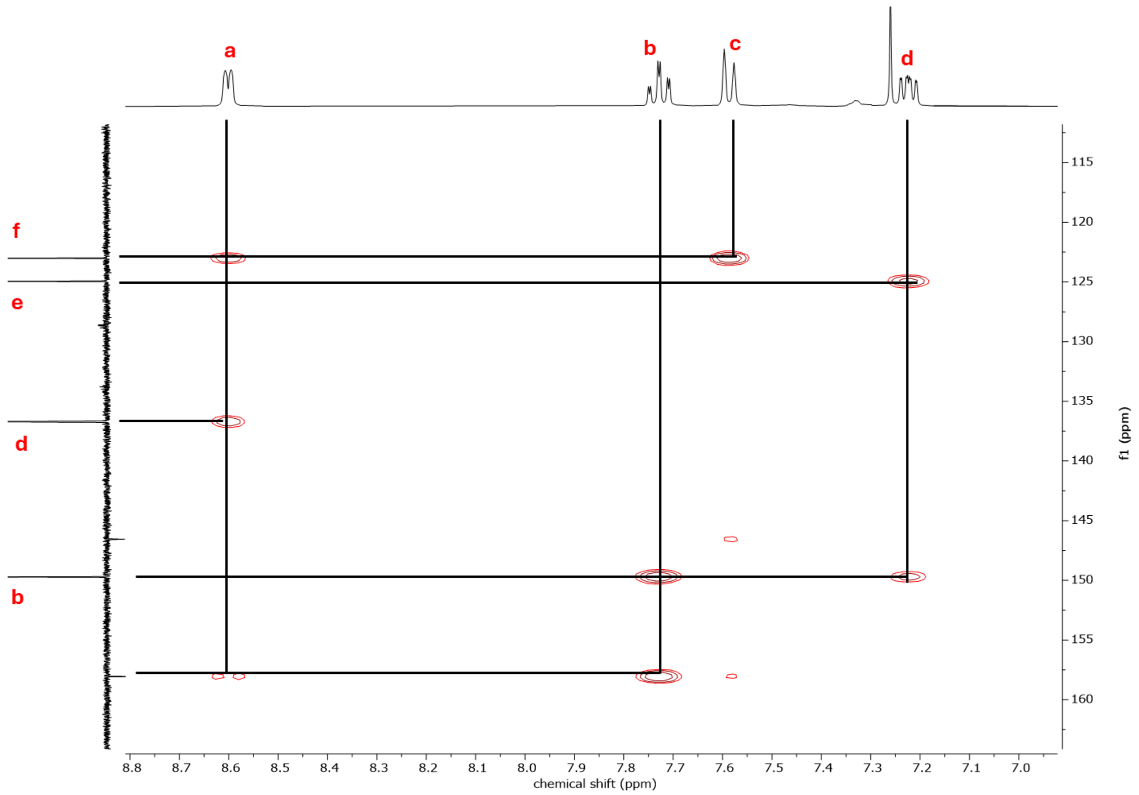

Figure S5: ^{1}H-^{13}C HMBC NMR spectrum of 2,2'-(2,2-dibromoethene-1,1-diyl)dipyridine **(BEP)** in $CDCl_3$, at 25 °C.

2,2'-(2,2-bis(4-(tert-butyl)phenyl)ethene-1,1-diyl)dipyridine (^{tBu}PEP)

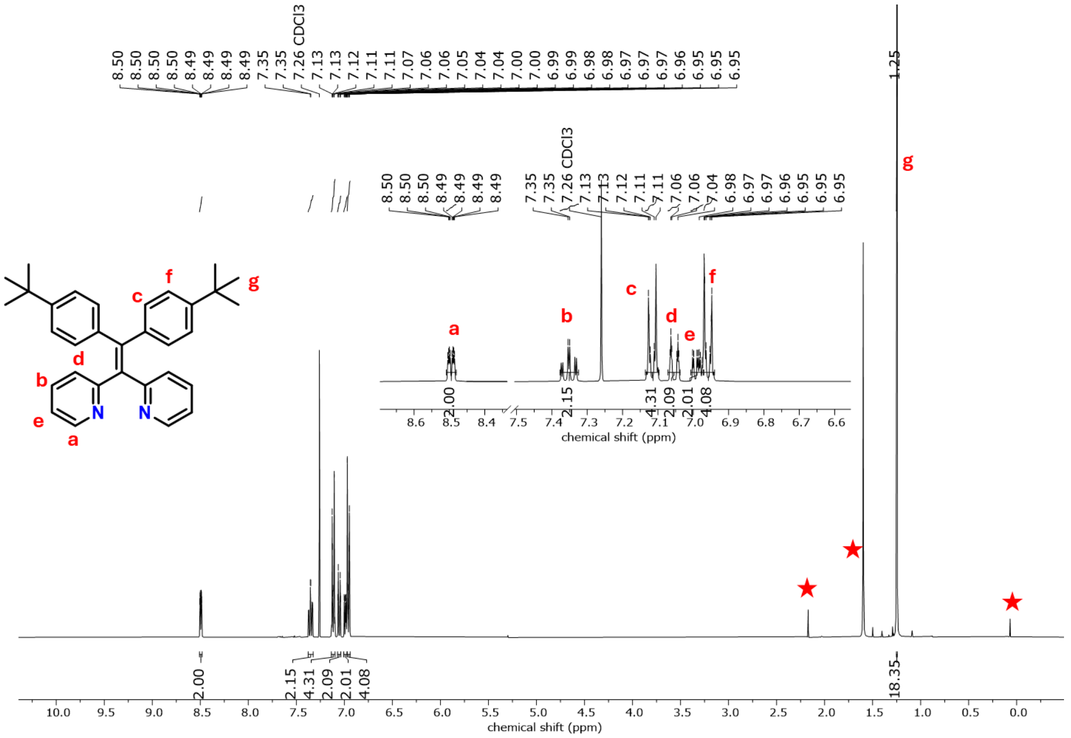

Figure S6: ^{1}H NMR spectrum of 2,2'-(2,2-bis(4-(*tert*-butyl)phenyl)ethene-1,1-diyl)dipyridine **(^{tBu}PEP)** in $CDCl_3$, at 25 °C. Resonances marked with a star are assigned to residual acetone (2.17 ppm), water (1.58 ppm) and grease (0.07).

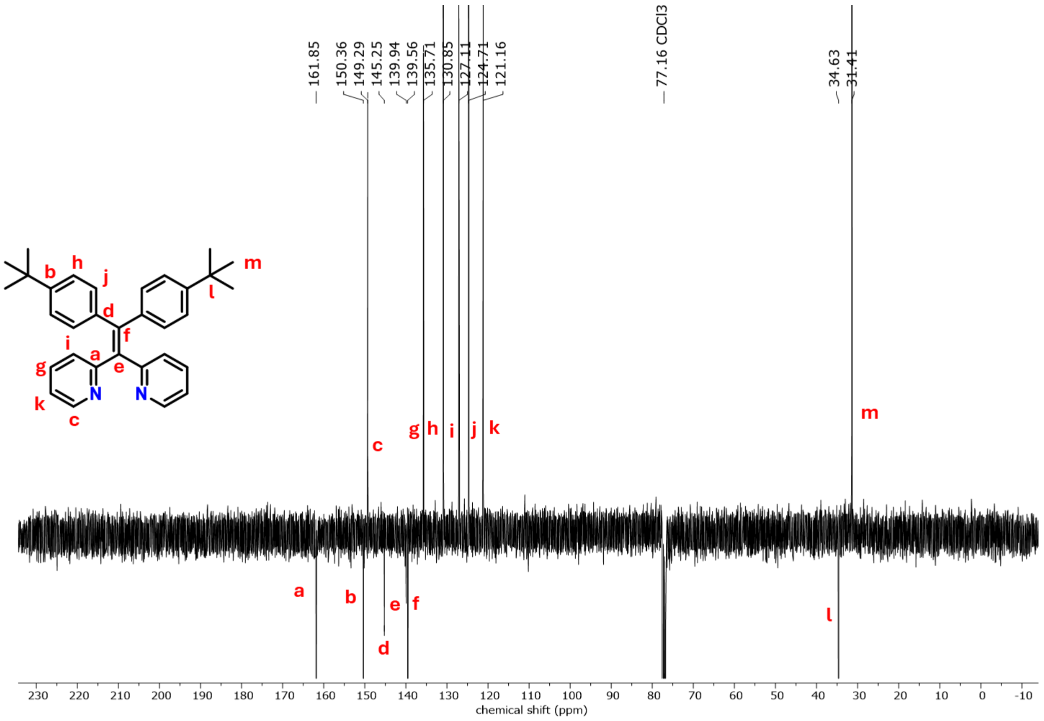

Figure S7: ^{13}C-APT NMR spectrum of 2,2'-(2,2-bis(4-(*tert*-butyl)phenyl)ethene-1,1-diyl)dipyridine (**^{tBu}PEP)** in $CDCl_3$, at 25 °C.

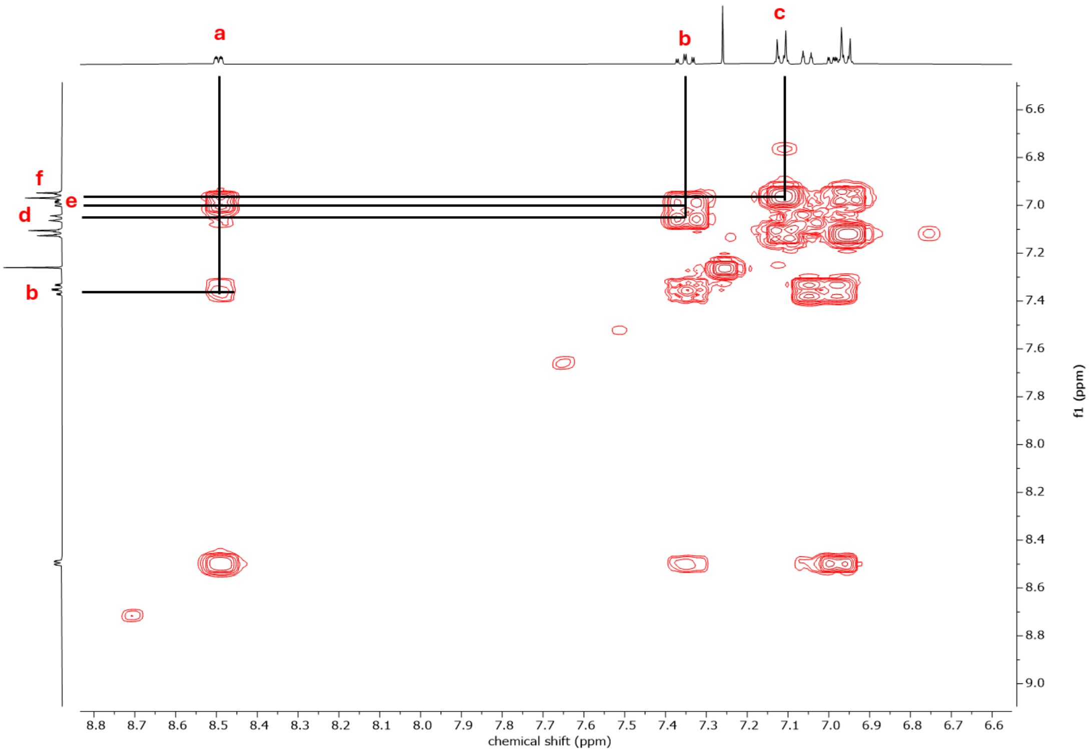

Figure S8: COSY NMR spectrum of 2,2'-(2,2-bis(4-(*tert*-butyl)phenyl)ethene-1,1-diyl)dipyridine **(^{tBu}PEP)** in $CDCl_3$, at 25 °C.

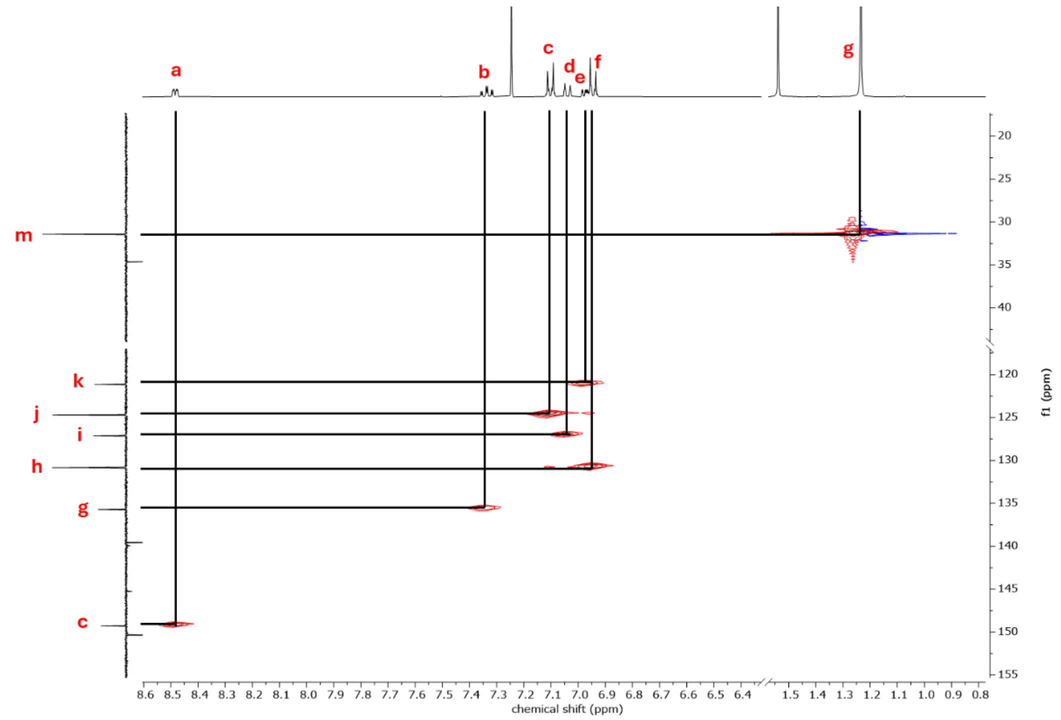

Figure S9: ^{1}H-^{13}C ASAP-HSQC NMR spectrum of 2,2'-(2,2-bis(4-(*tert*-butyl)phenyl)ethene-1,1-diyl)dipyridine **(^{tBu}PEP)** in $CDCl_3$, at 25 °C.

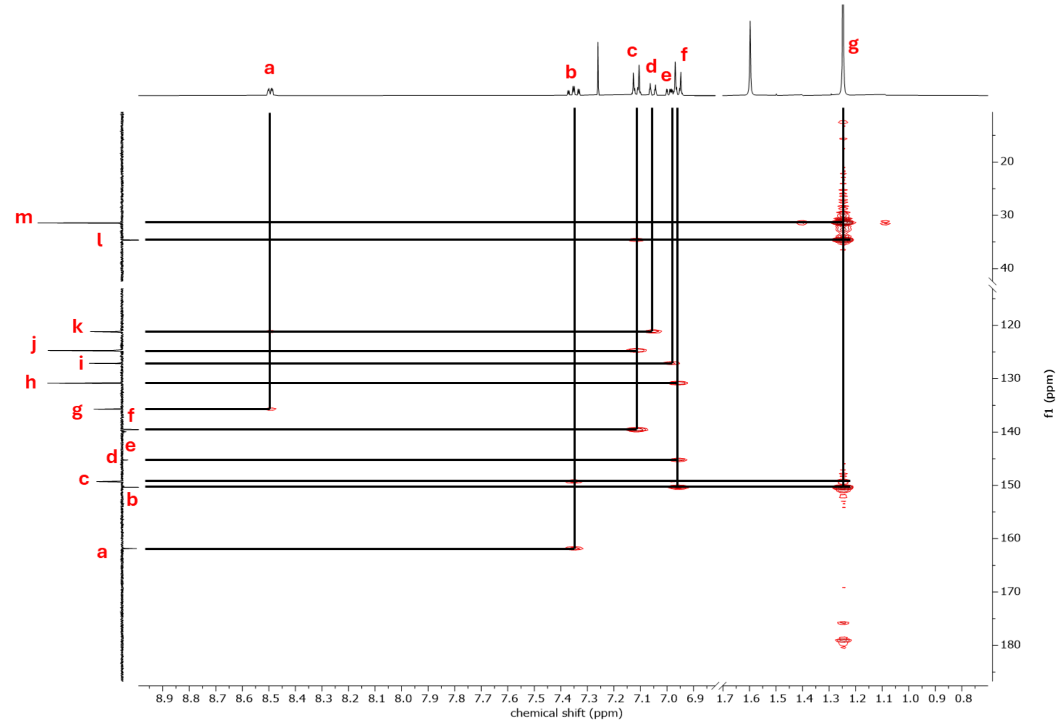

Figure S10: ^{1}H-^{13}C HMBC NMR spectrum of **2,2'-(2,2-bis(4-(*tert*-butyl)phenyl)ethene-1,1-diyl)dipyridine (^{tBu}PEP)** in $CDCl_3$, at 25 °C.

9-(*tert*-butyl)-6-(4-(*tert*-butyl)phenyl)-5-(pyridin-2-yl)benzo[*f*]quinoline (^{tBu}PPBQ)

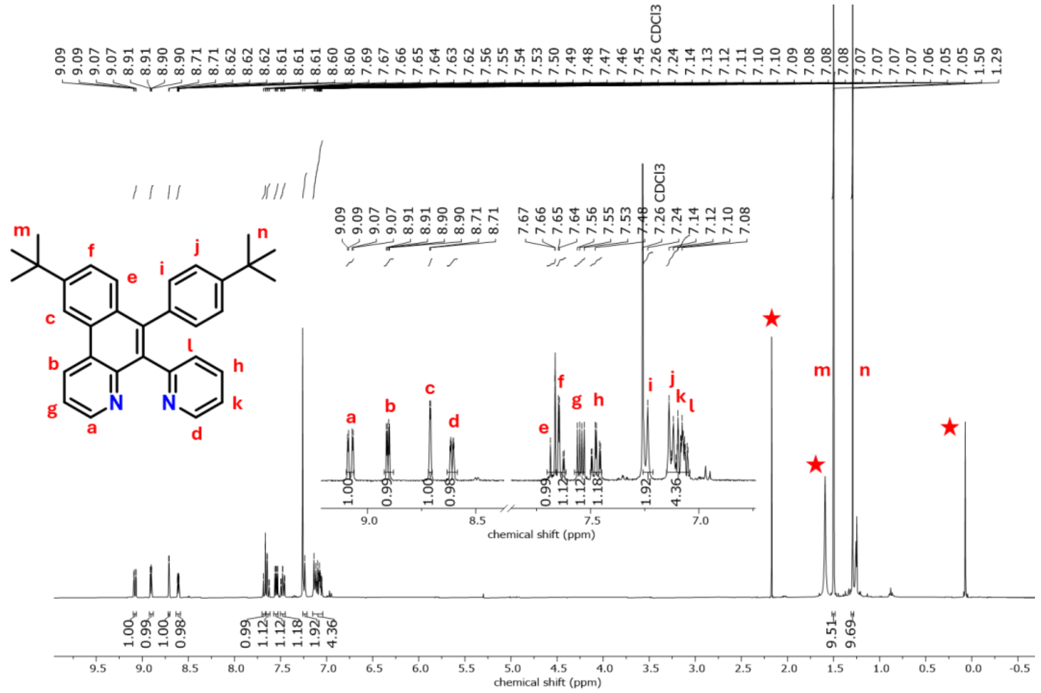

Figure S11: ^{1}H NMR spectrum of **9**-(*tert*-butyl)-6-(4-(*tert*-butyl)phenyl)-5-(pyridin-2-yl)benzo[*f*]quinoline **(^{tBu}PPBQ)** in $CDCl_3$, at 25 °C. Resonances marked with a star are assigned to residual acetone (2.17 ppm), water (1.58 ppm) and grease (0.07).

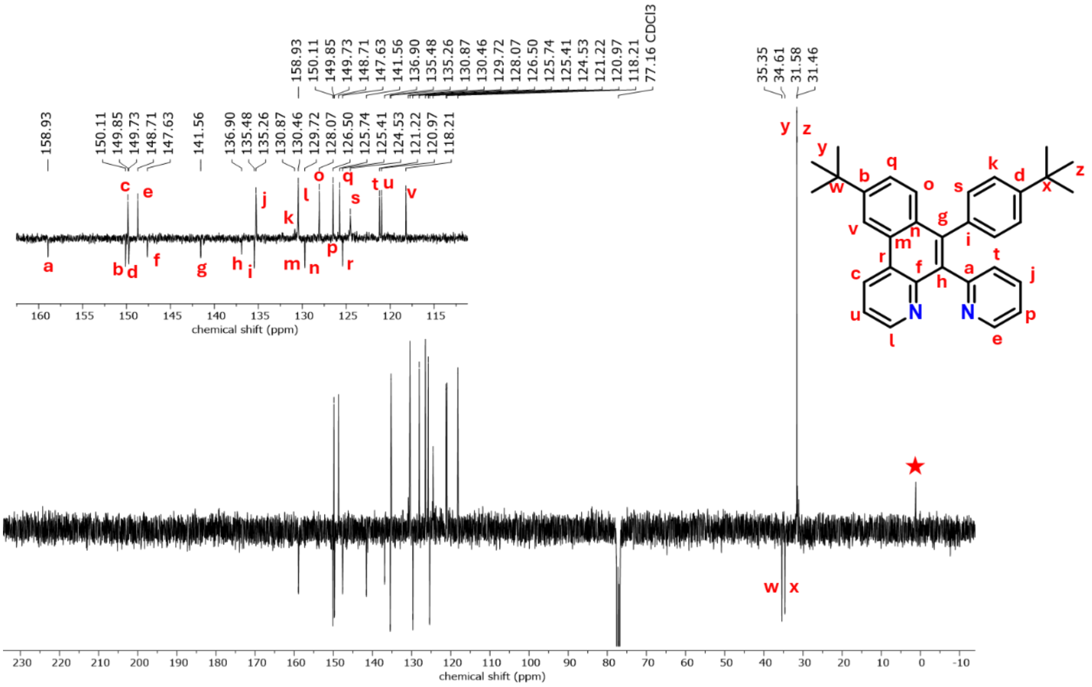

Figure S12: ^{13}C-APT NMR spectrum of 9-(*tert*-butyl)-6-(4-(*tert*-butyl)phenyl)-5-(pyridin-2-yl)benzo[*f*]quinoline **(^{tBu}PPBQ)** in $CDCl_3$, at 25 °C. Resonance marked with a star is assigned to grease (1.2 ppm).

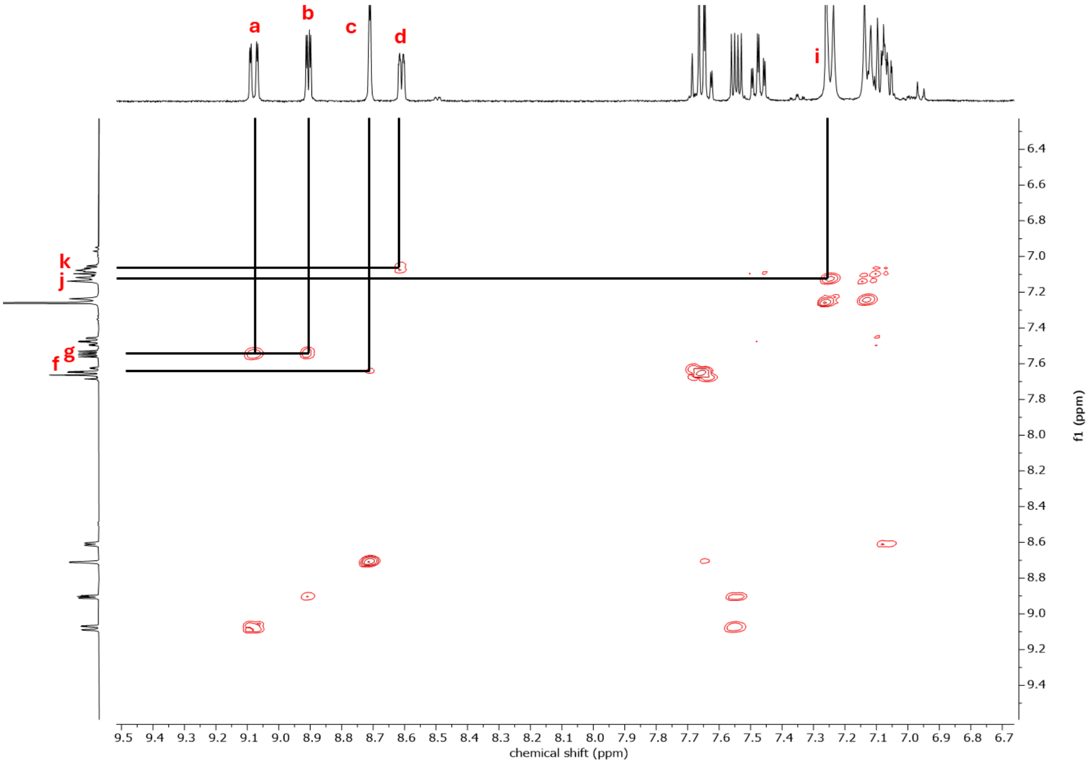

Figure S13: gCOSY NMR spectrum of 9-(*tert*-butyl)-6-(4-(*tert*-butyl)phenyl)-5-(pyridin-2-yl)benzo[*f*]quinoline (**^{tBu}PPBQ)** in $CDCl_3$, at 25 °C.

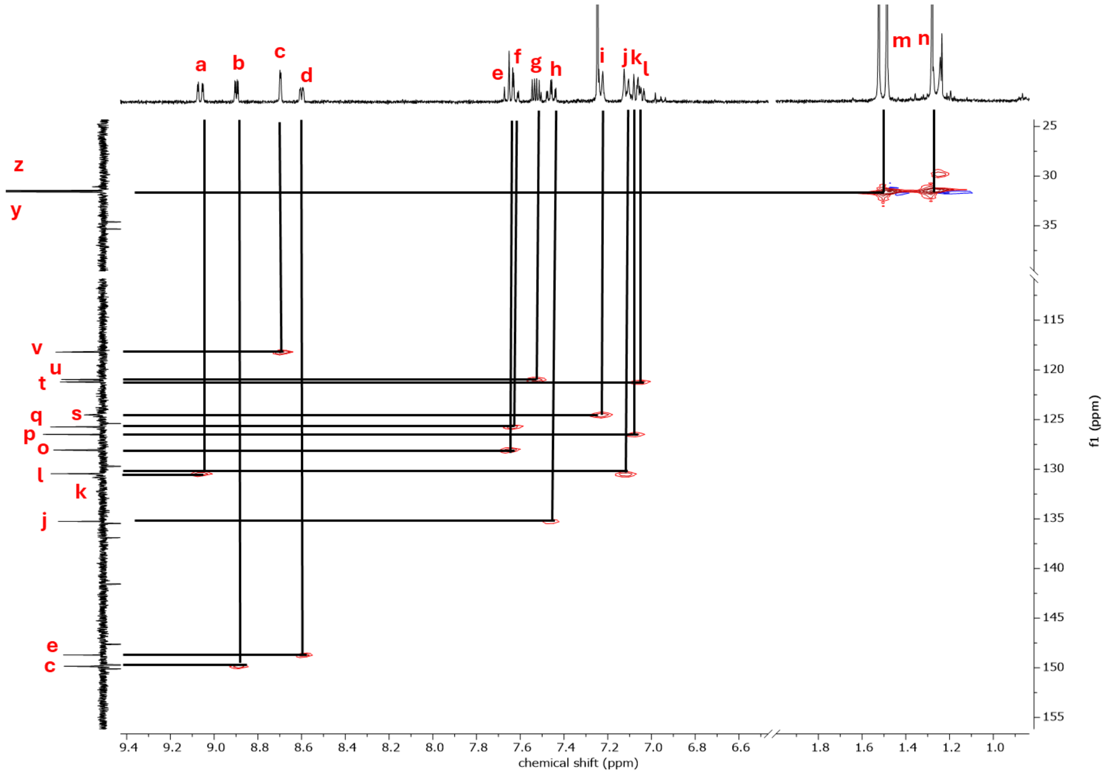

Figure S14: ASAP-HMQC NMR spectrum of 9-(*tert*-butyl)-6-(4-(*tert*-butyl)phenyl)-5-(pyridin-2-yl)benzo[*f*]quinoline (**^{tBu}PPBQ)** in $CDCl_3$, at 25 °C.

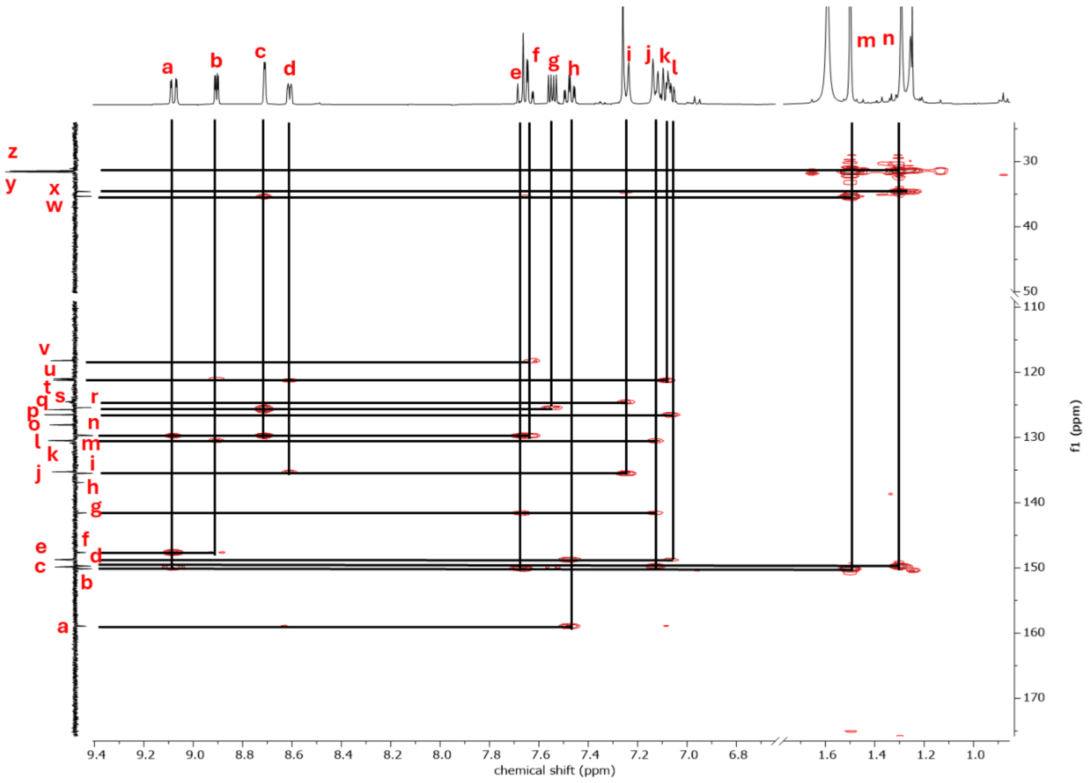

Figure S15: gHMBC NMR spectrum of 9-(*tert*-butyl)-6-(4-(*tert*-butyl)phenyl)-5-(pyridin-2-yl)benzo[*f*]quinoline **(^{tBu}PPBQ)** in $CDCl_3$, at 25 °C.

[Zn(^{tBu}PEP)Cl_2]

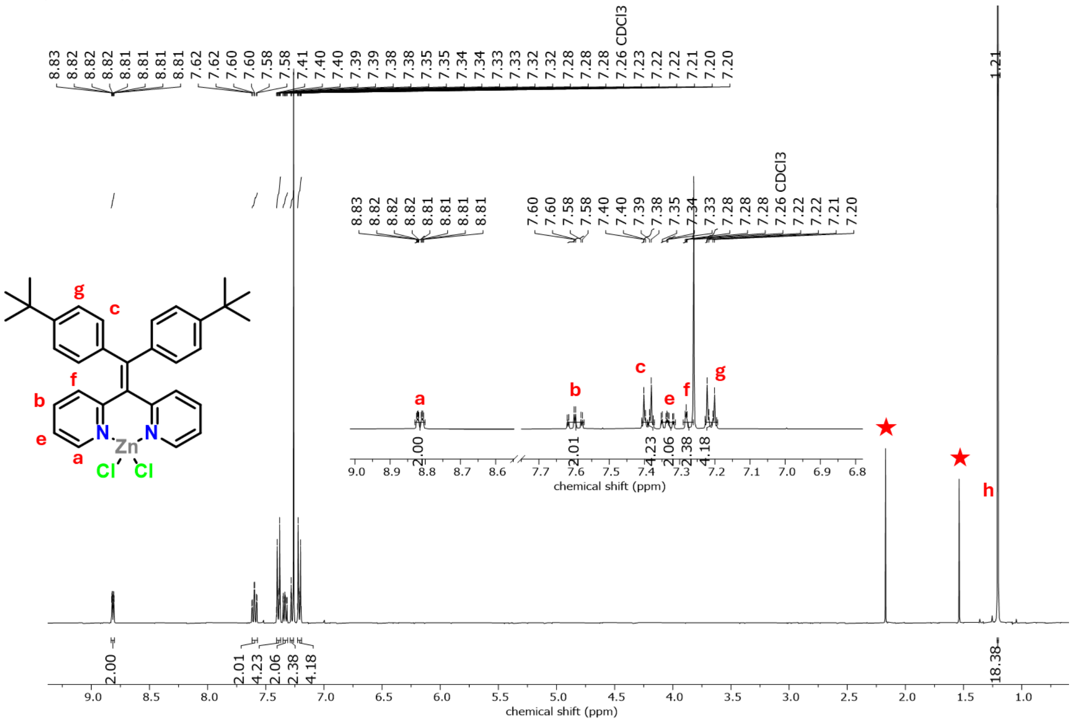

Figure S16: ^{1}H NMR spectrum of **[Zn(^{tBu}PEP)Cl_2]** in $CDCl_3$, at 25 °C. The resonances marked with a star are attributed to residual acetone (2.17 ppm) and water (1.54 ppm).

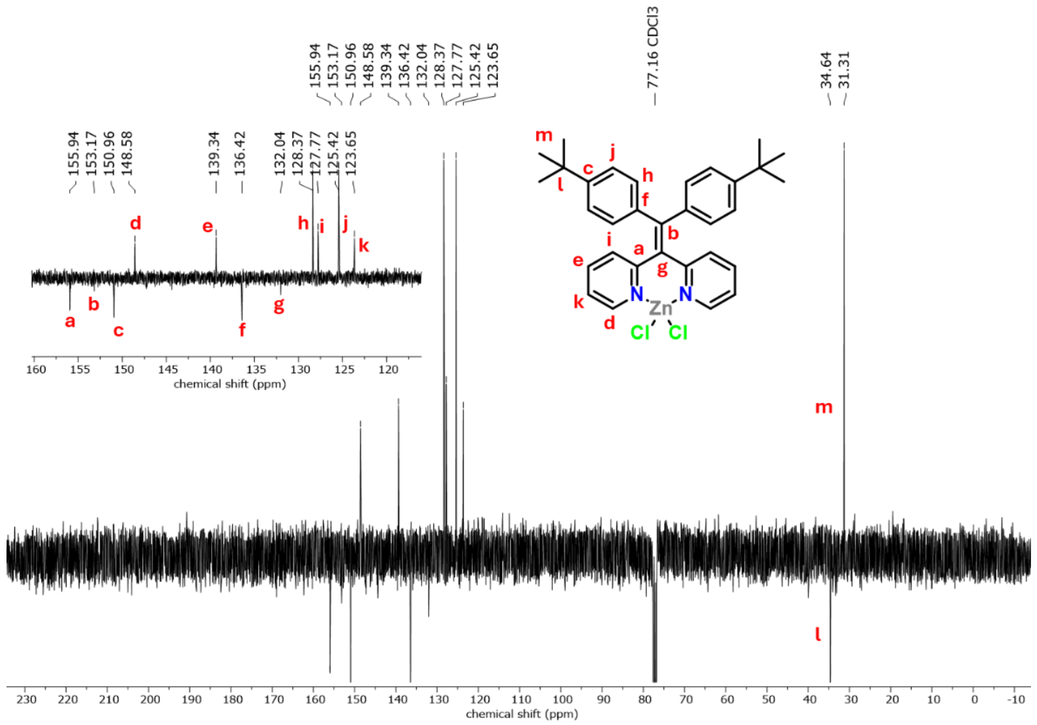

Figure S17: ^{13}C-APT NMR spectrum of **[Zn(^{tBu}PEP)Cl$_2$]** in $CDCl_3$, at 25 °C.

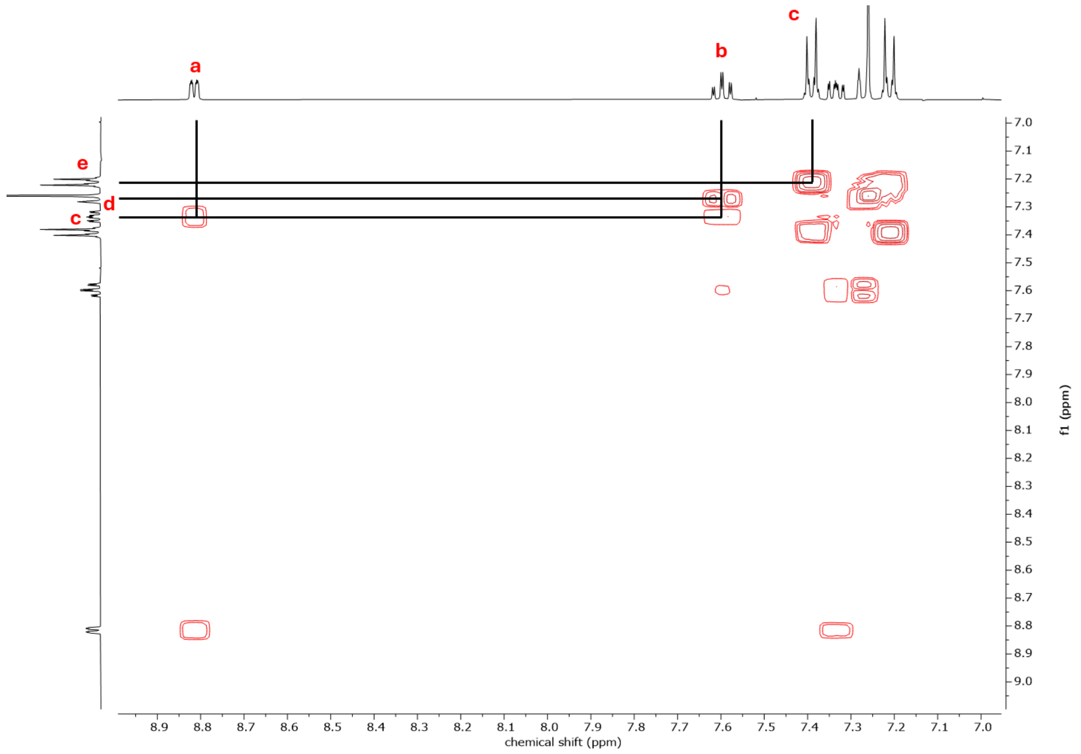

Figure S18: COSY NMR spectrum of **[Zn(^{tBu}PEP)Cl$_2$]** in $CDCl_3$, at 25 °C.

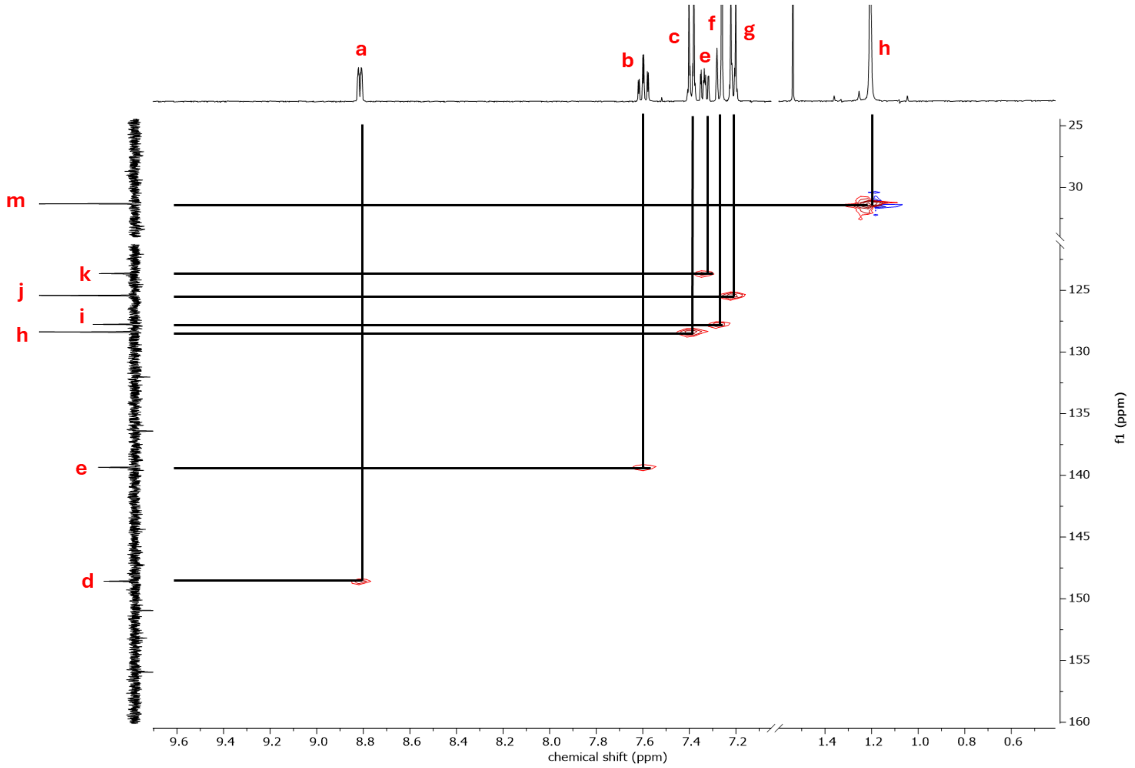

Figure S19: ^{1}H-^{13}C ASAP-HMQC NMR spectrum of **[Zn(^{tBu}PEP)Cl$_2$]** in $CDCl_3$, at 25 °C.

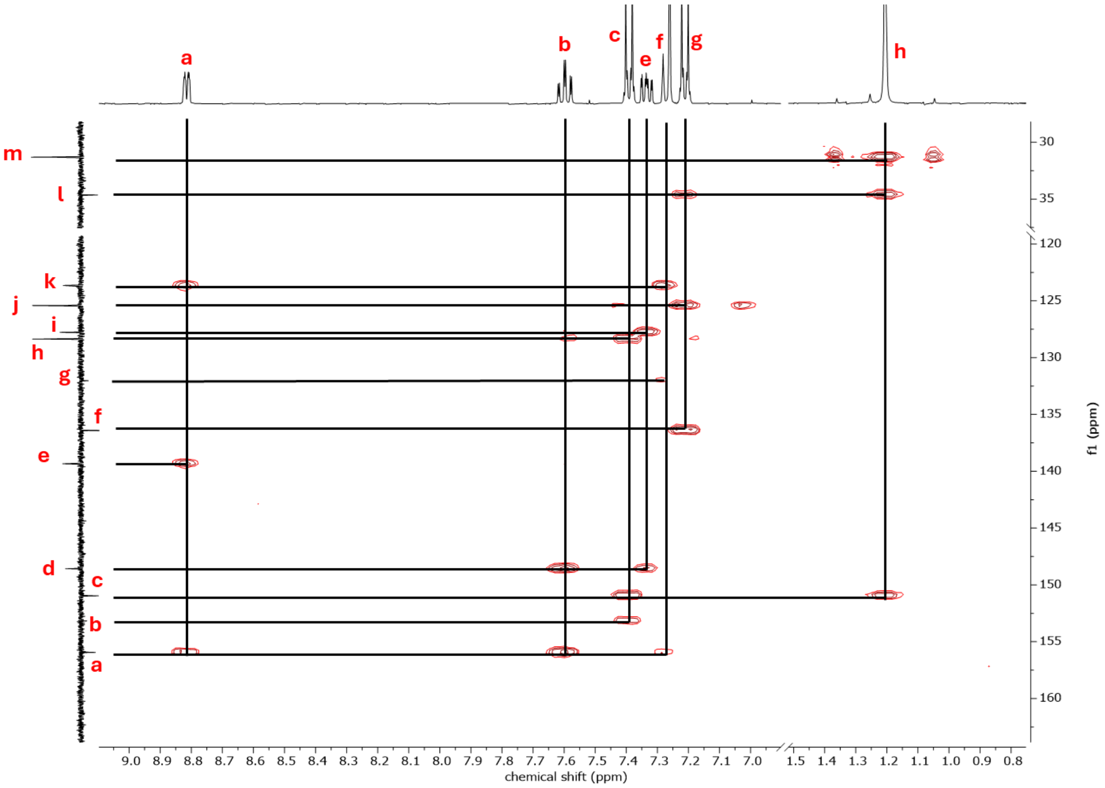

Figure S20: ^{1}H-^{13}C HMBC NMR spectrum of **[Zn(^{tBu}PEP)Cl$_2$]** in $CDCl_3$, at 25 °C.

[Zn(^{tBu}PPBQ)Cl$_2$]

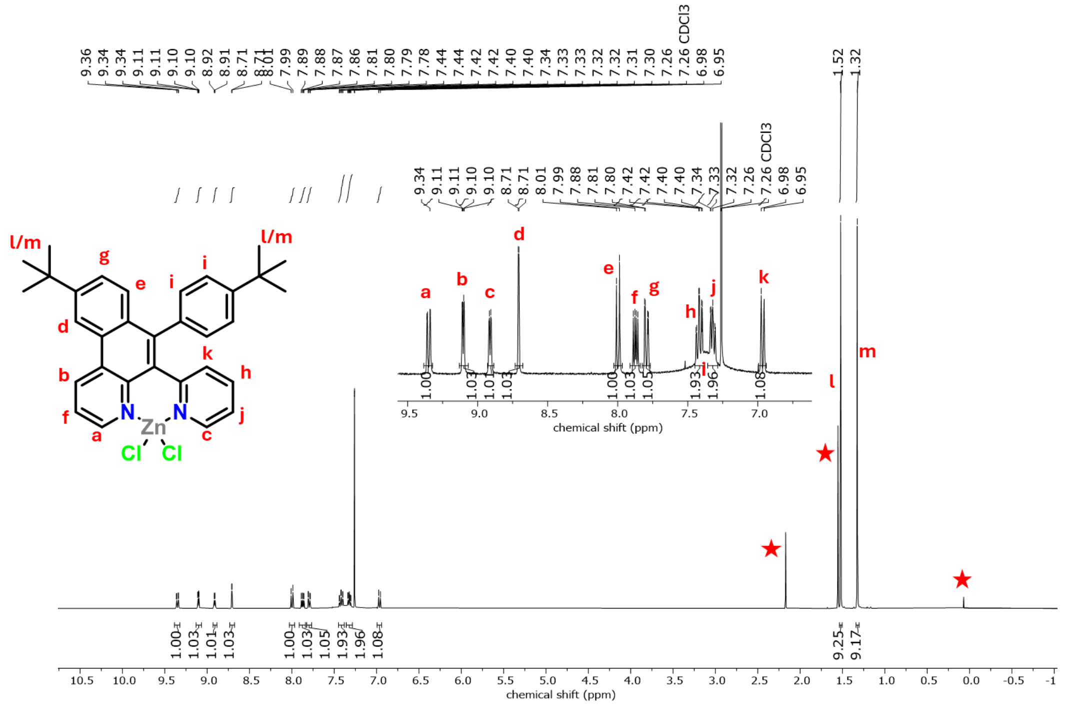

Figure S21: ^{1}H NMR spectrum of **[Zn(^{tBu}PPBQ)Cl$_2$]** in CDCl$_3$, at 25 °C. The resonances marked with a star are assigned to residual acetone (2.17 ppm), water (1.55 ppm) and laboratory grease (0.07 ppm).

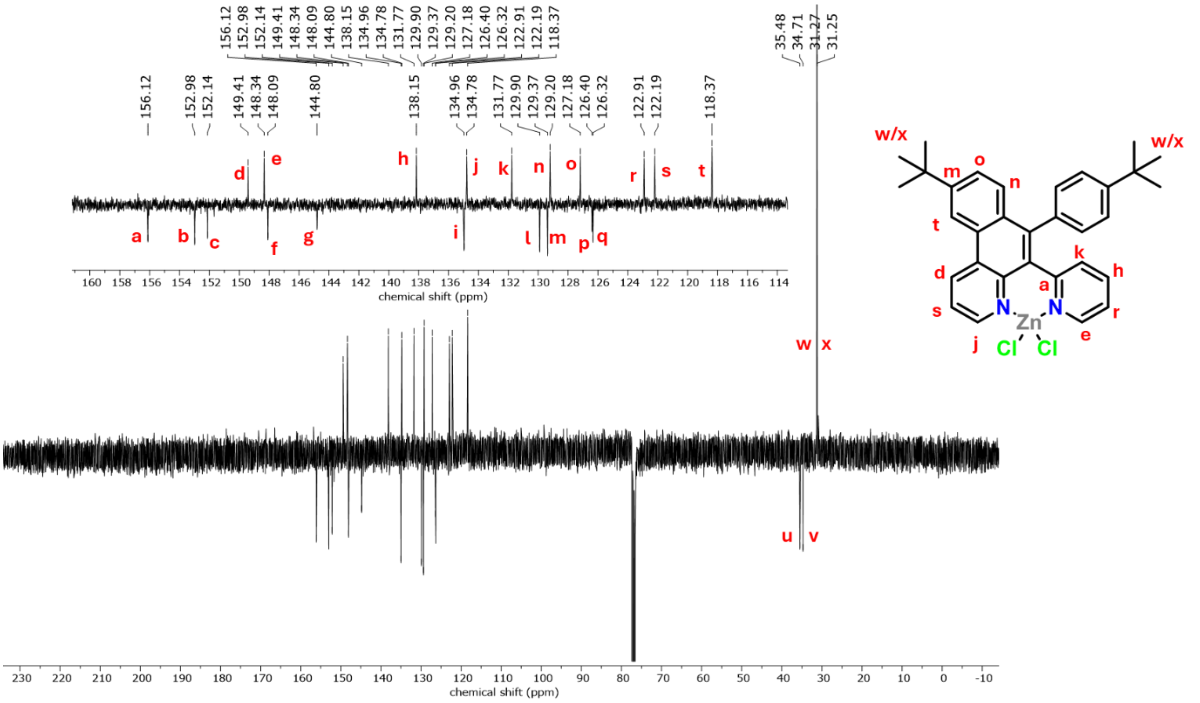

Figure S22: ^{13}C-APT NMR spectrum of **[Zn(^{tBu}PPBQ)Cl$_2$]** in CDCl$_3$, at 25 °C.

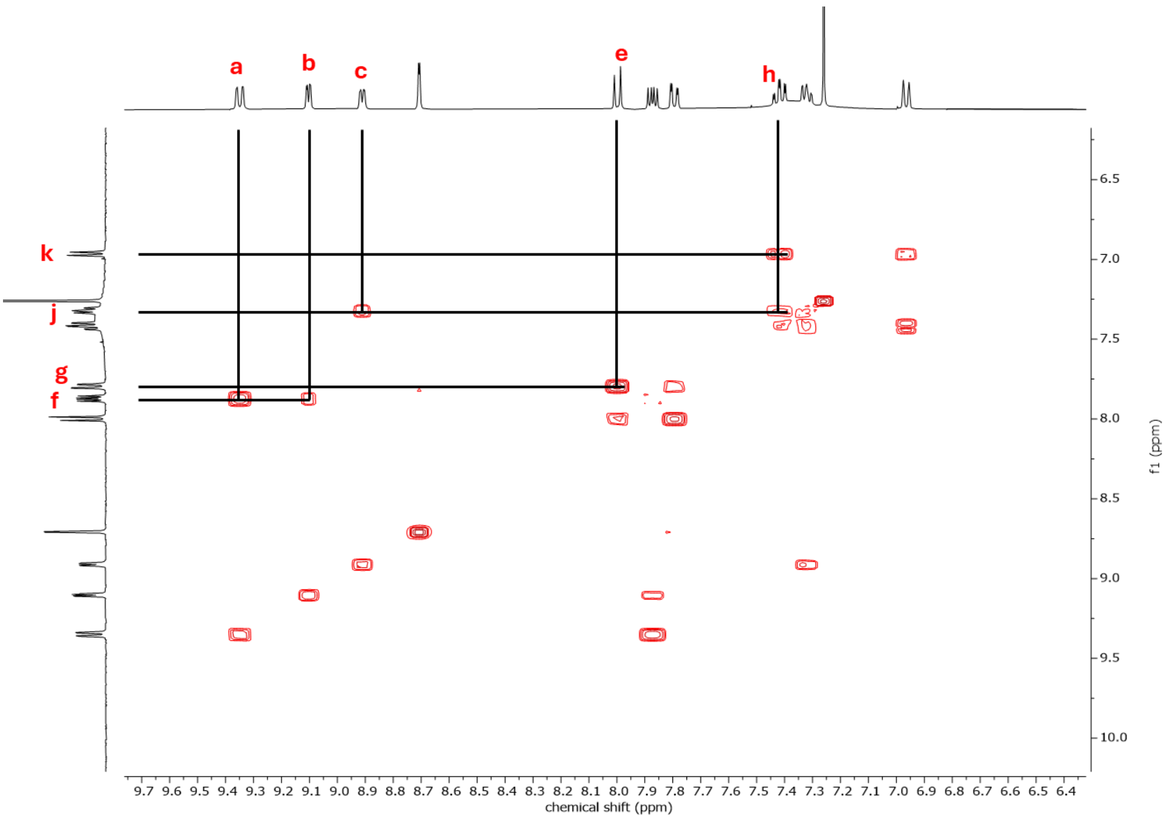

Figure S23: gCOSY NMR spectrum of **[Zn(^{tBu}PPBQ)Cl$_2$]** in $CDCl_3$, at 25 °C.

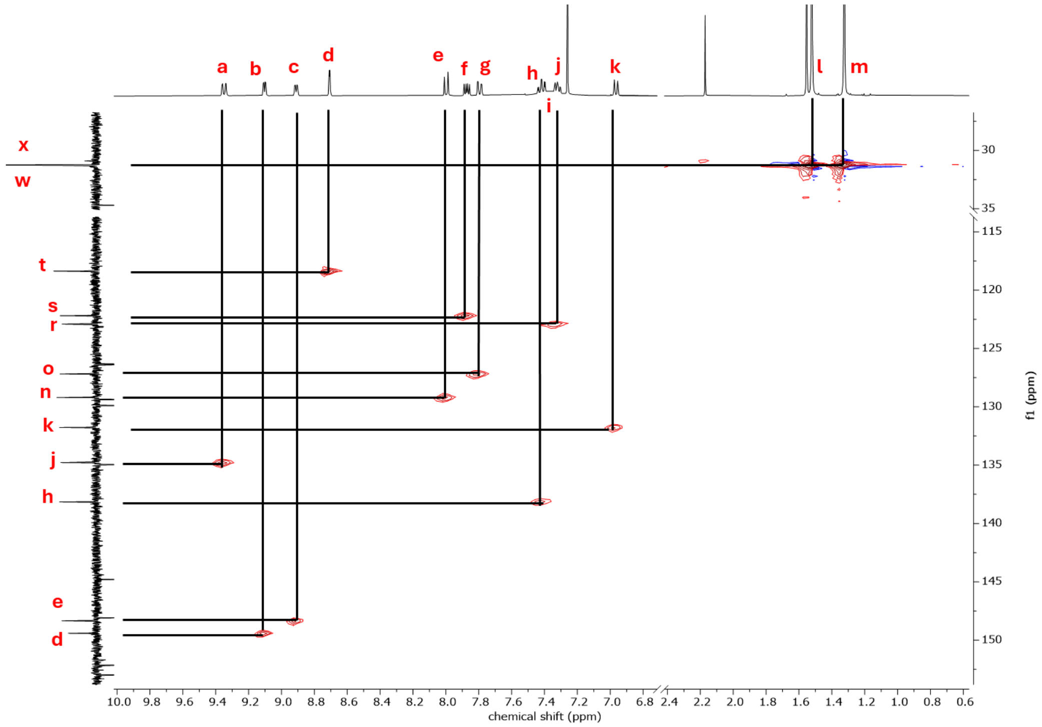

Figure S24: ^{1}H-^{13}C ASAP-HMQC NMR spectrum of **[Zn(^{tBu}PPBQ)Cl$_2$]** in $CDCl_3$, at 25 °C.

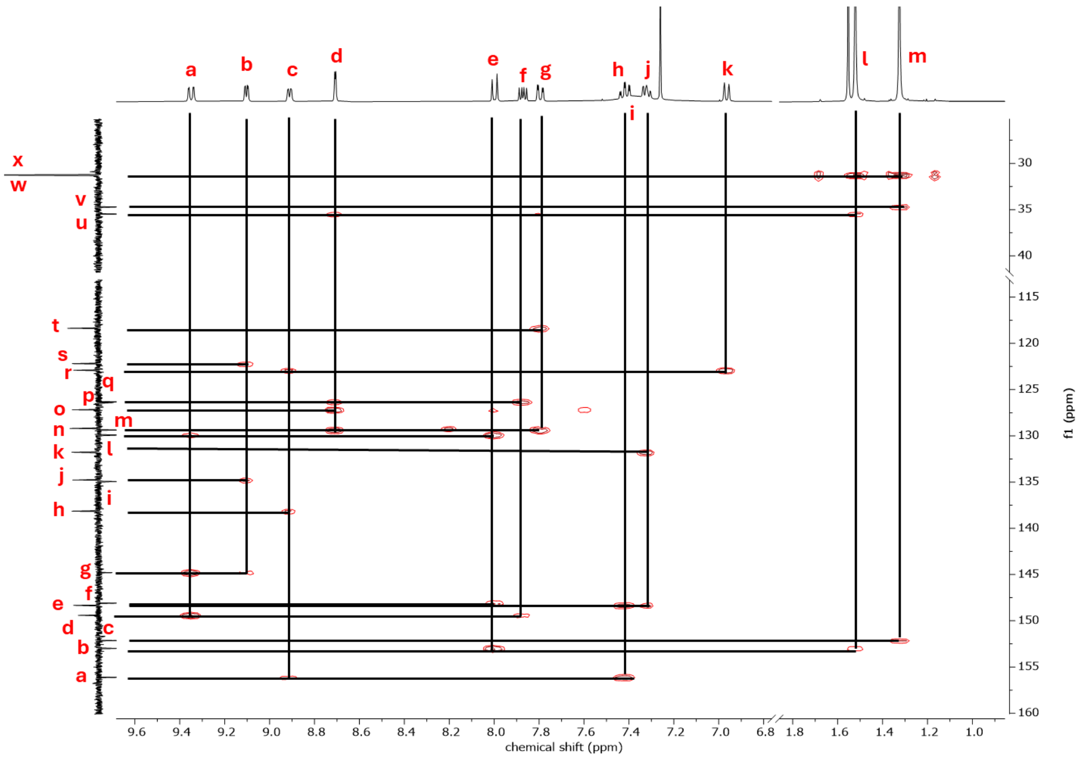

Figure S25: ^{1}H-^{13}C HMBC NMR spectrum of **[Zn(^{tBu}PPBQ)Cl$_2$]** in CDCl$_3$, at 25 °C.

[Ni(^{tBu}PEP)$_2$Cl$_2$]

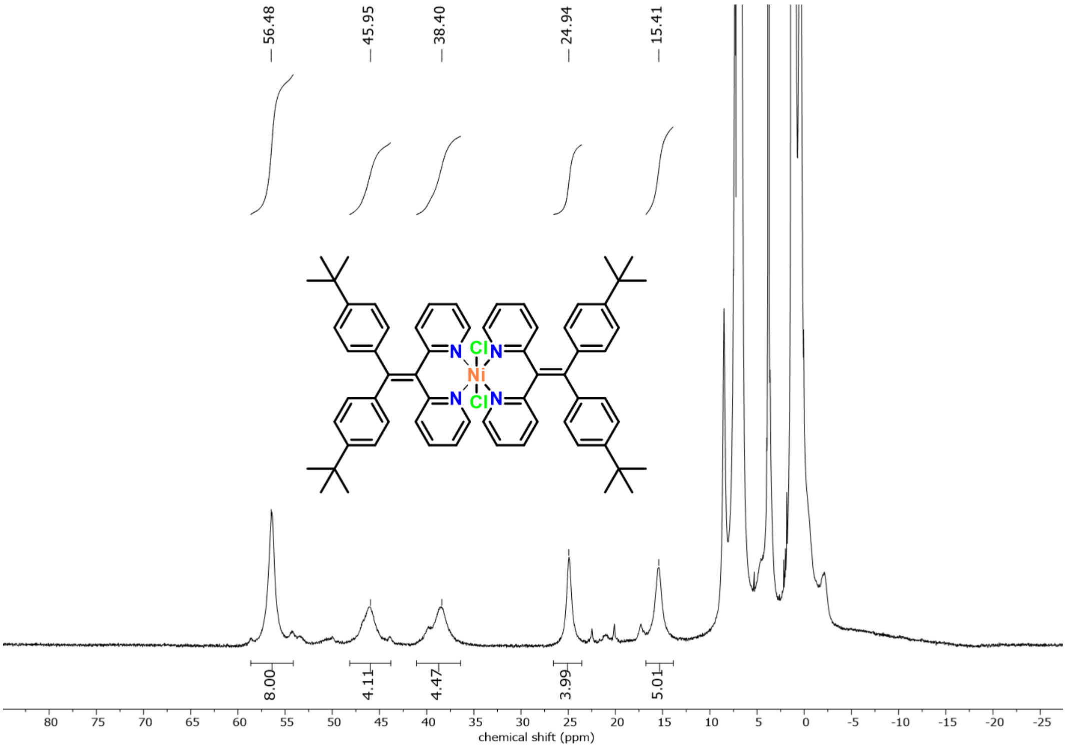

Figure S26: ^{1}H NMR spectrum of **[Ni(^{tBu}PEP)$_2$Cl$_2$]** in CDCl$_3$, at 25 °C.

[Ni(^{tBu}PPBQ)$_2$Cl$_2$]

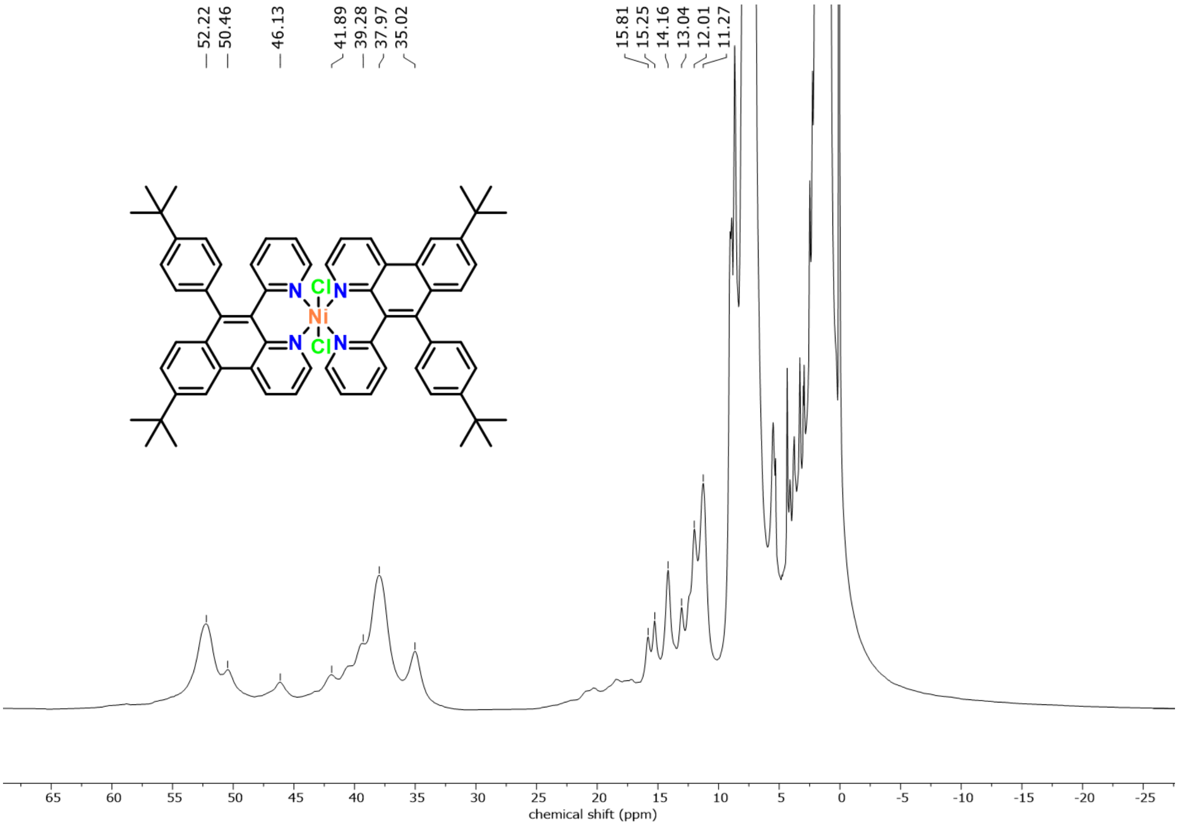

Figure S27: ^{1}H NMR spectrum of **[Ni(^{tBu}PPBQ)$_2$Cl$_2$]** in $CDCl_3$, at 25 °C.

Catalyst decomposition experiment

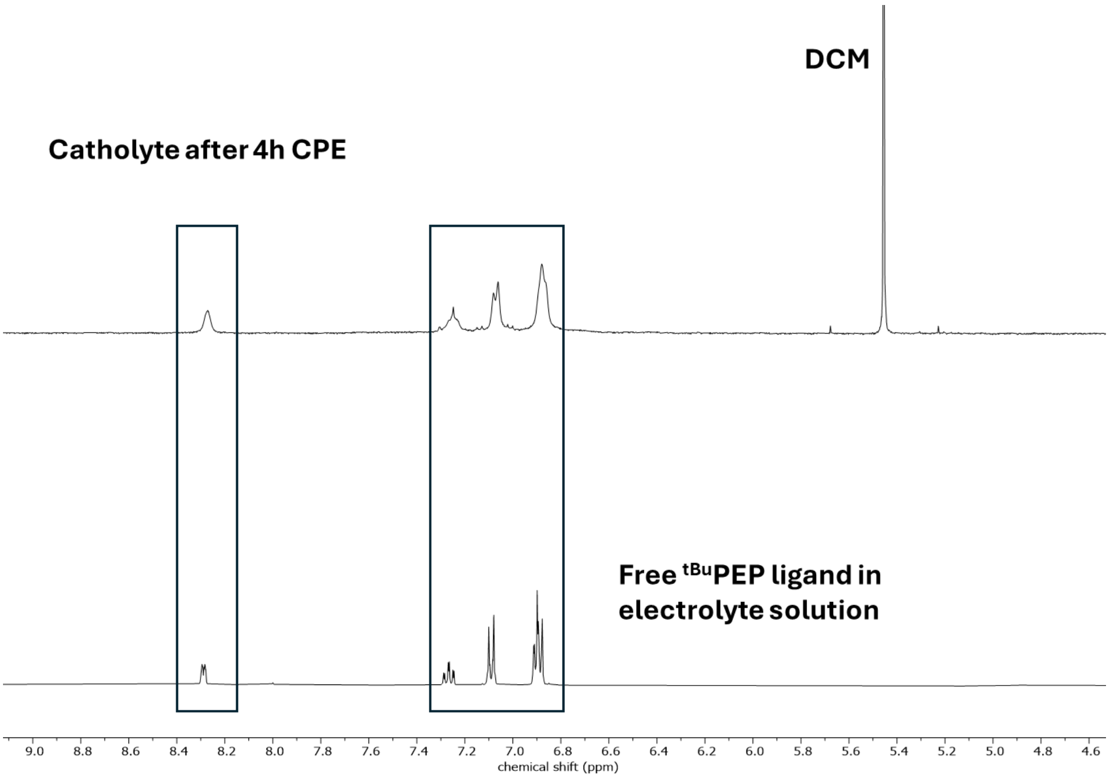

Figure S28: Comparison of the ^{1}H NMR spectrum of the catholyte solution after approximately 4 hours of CPE (see Figure S38) and the free **^{tBu}PEP** ligand in electrolyte solution. For the CPE experiment, 30

mM DCM was used as substrate, and 3 mM **[Ni(^{tBu}PEP)$_2$Cl$_2$]**. A potential of -2.83 V vs. Fc/Fc$^+$ was maintained throughout the experiment. Measurements were performed in an H-cell with ~3 mL volume for both the catholyte and anolyte compartments. During CPE, both catholyte and anolyte were continuously stirred using a Teflon-coated stir bar. Measurements were performed in THF, 0.2 M $N^nBu_4PF_6$ using a Pt wire CE and a $Ag/AgNO_3$ (saturated solution in the electrolyte) RE.

S4. UV-VIS

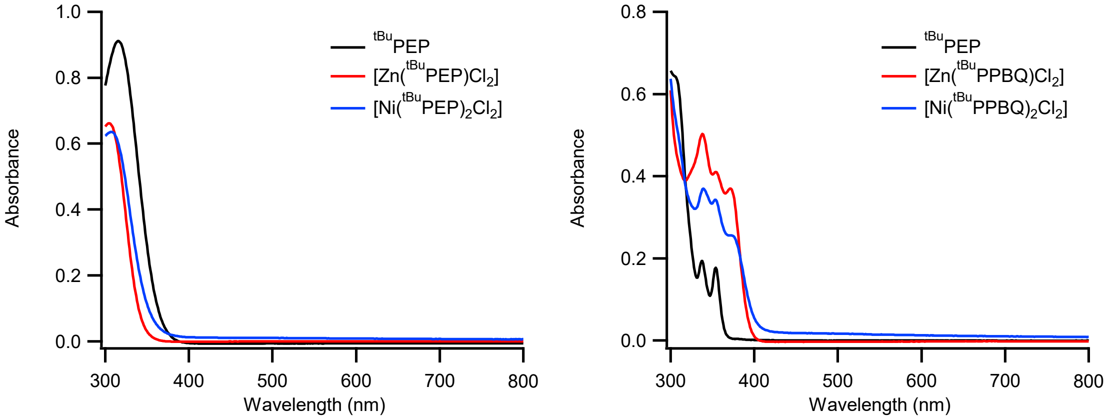

Figure S29: UV-Vis spectra of **tBuPEP**, **[Zn(tBuPEP)Cl$_2$]** and **[Ni(tBuPEP)$_2$Cl$_2$]** (left) and the UV-Vis spectra of **tBuPPBQ**, **[Zn(tBuPPBQ)Cl$_2$]** and **[Ni(tBuPPBQ)$_2$Cl$_2$]** (right). All spectra were recorded of a 20 µM solution in DCM.

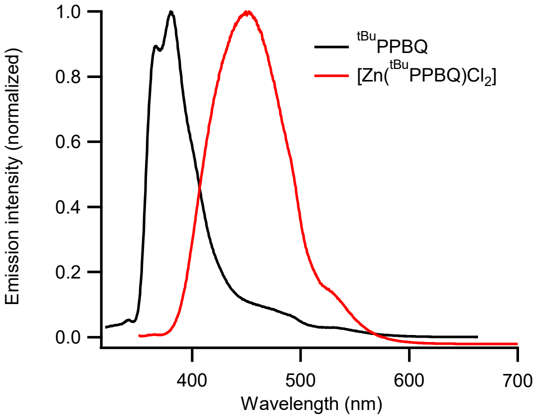

Figure S30: Emission spectra of **tBuPPBQ** (10 µM; excitation wavelength: 310 nm) and **[Zn(tBuPPBQ)$_2$Cl$_2$]** (10 µM; excitation wavelength: 260 nm). All spectra were recorded of a 20 µM solution in DCM.

S5. Electrochemistry

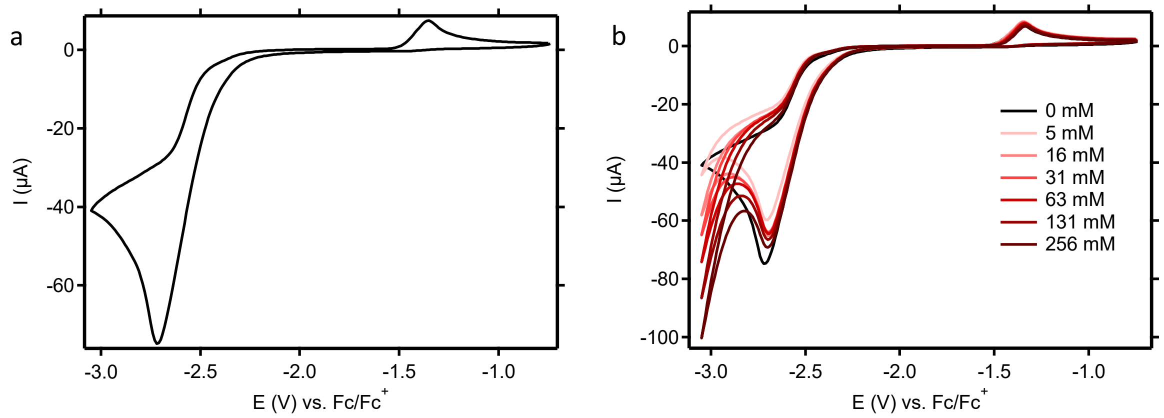

Figure S31: a) CV trace of **^{tBu}PEP** in the absence of DCM and b) with increasing DCM concentration. Measurement was performed in THF, 0.2 M $N^{n}Bu_4PF_6$ using a glassy carbon WE, a Pt wire CE and a $Ag/AgNO_3$ (saturated solution in the electrolyte) RE.

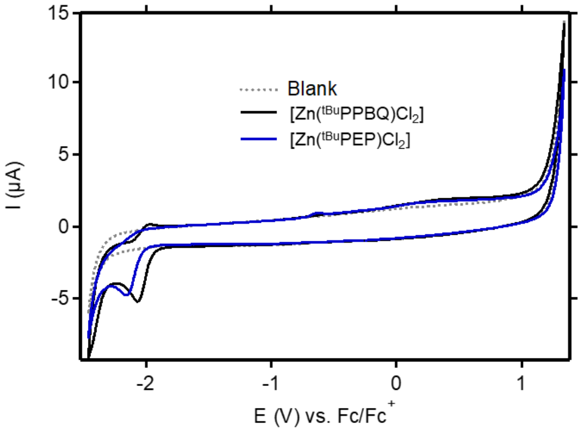

Figure S32: CV traces of **[Zn(^{tBu}PEP)Cl_2]** and **[Zn(^{tBu}PPBQ)Cl_2]**. Measurement was performed in DCM, 0.1 M $N^{n}Bu_4PF_6$ using a glassy carbon WE, a Pt wire CE and a $Ag/AgNO_3$ (saturated solution in the electrolyte) RE.

Catalytic activity and kinetic analysis

For both **[Ni(^{tBu}PEP)$_2$$Cl_2$]** and **[Ni(^{tBu}PPBQ)$_2$$Cl_2$]** a catalytic current increase is observed when DCM is added to the THF electrolyte (Figure S33a,b). For both, the onset potential of the main catalytic wave seems to correspond mostly to the third (most cathodic) reduction event (onset potentials of approximately -2.55 and -2.40 V vs. Fc/Fc^+ for **[Ni(^{tBu}PEP)$_2$$Cl_2$]** and **[Ni(^{tBu}PPBQ)$_2$$Cl_2$]**, respectively), although the currents slowly increase at lower overpotentials, giving a shoulder to the main catalytic wave (onset potentials of approximately -2.1 V vs. Fc/Fc^+ for both **[Ni(^{tBu}PEP)$_2$$Cl_2$]** and **[Ni(^{tBu}PPBQ)$_2$$Cl_2$]**). The shapes of the catalytic waves between both complexes show notable differences. Whereas for **[Ni(^{tBu}PEP)$_2$$Cl_2$]**, the catalytic waves are mostly peak shaped at low substrate concentrations, for **[Ni(^{tBu}PPBQ)$_2$$Cl_2$]** the catalytic waves are more S-shaped at low substrate concentrations and generally reach higher currents, implying a higher reaction rate for the reaction catalysed by **[Ni(^{tBu}PPBQ)$_2$$Cl_2$]** than **[Ni(^{tBu}PEP)$_2$$Cl_2$]**. No catalytic current was observed using the **^{tBu}PEP** ligand without a metal centre under the same conditions (Figure S31b).

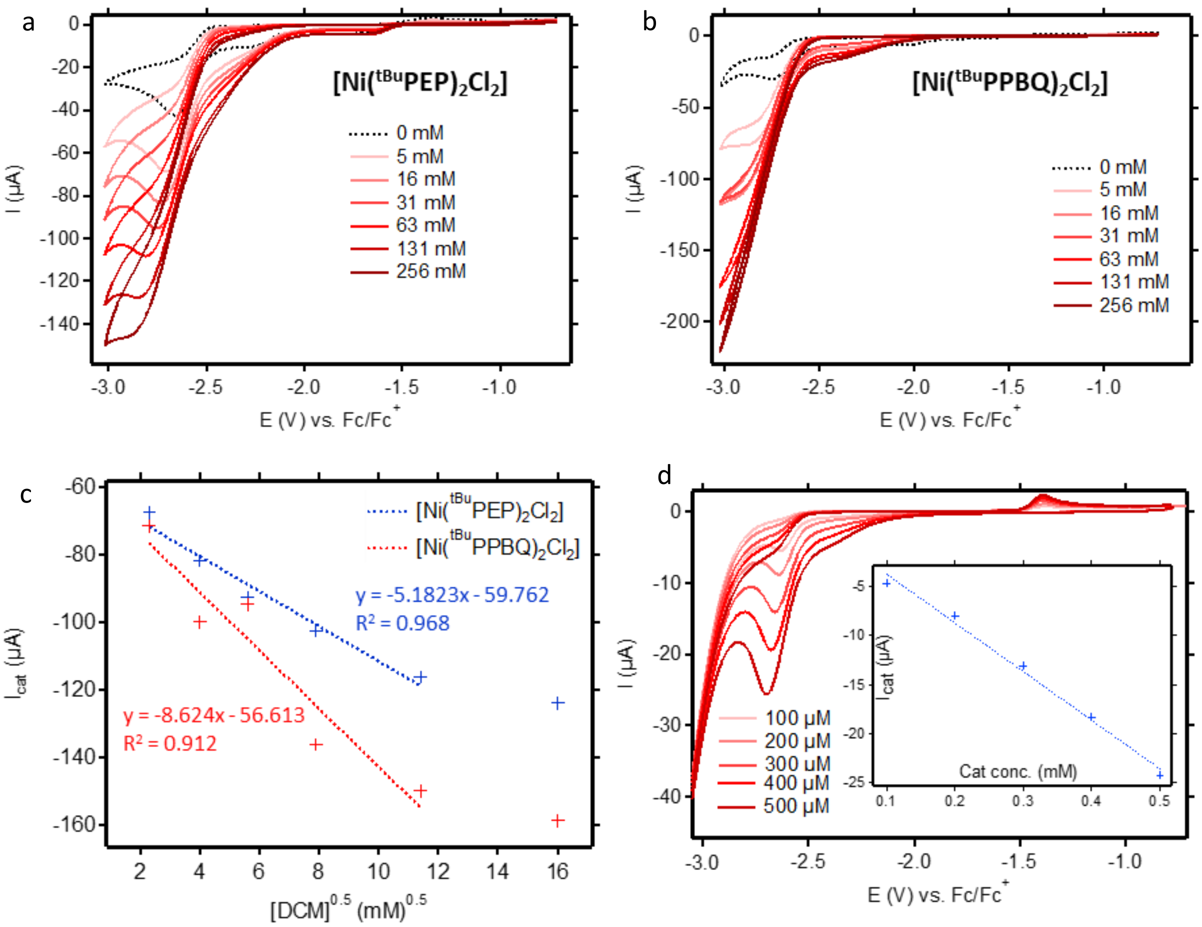

Figure S33: CV traces of a) **[Ni(^{tBu}PEP)$_2$Cl$_2$]** and b) **[Ni(^{tBu}PPBQ)$_2$Cl$_2$]** with varying concentrations of DCM. c) Linear fits of the blank-corrected catalytic currents of the reduction of DCM. d) CV traces of varying **[Ni(^{tBu}PEP)$_2$Cl$_2$]** concentration, with 128 mM of DCM; the inset shows the linear fit of the blank-corrected catalytic peak current versus the catalyst concentration.

Since DCM reduces on the glassy carbon electrode itself at high overpotentials, the blank measurements with DCM but without catalyst were subtracted from the catalytic currents in order to obtain kinetic information (Figure S34). Using these blank-corrected measurements, the catalytic currents were plotted against the square root of the DCM concentration (Figure S33c). Both **[Ni(^{tBu}PEP)$_2$Cl$_2$]** and **[Ni(^{tBu}PPBQ)$_2$Cl$_2$]** show first order in DCM. This behaviour is evidenced by the linear relationship between the catalytic current (I_{cat}) and the square root of the observed rate constant, as described by eq. 1.

$$\frac{I_{cat}}{I_p} = \frac{n}{0.4463n'}\sqrt{\frac{RT}{n'Fv}k_{obs}} \qquad (1)$$

Where I_{cat} is the catalytic current, I_p is the peak current in absence of substrate, n is the number of electrons transferred in the reaction, n' is the number of electrons transferred to the catalyst in the absence of substrate, k_{obs} is the observed rate constant (*i.e.* TOF), v is the scan rate and R, T and F are the gas constant, temperature and Faraday's constant, respectively.[3,4] Because of uncertainty in the mechanism, and therefore the number of electrons transferred in each step, eq. 1 could not be used to calculate the observed rate constant k_{obs}.

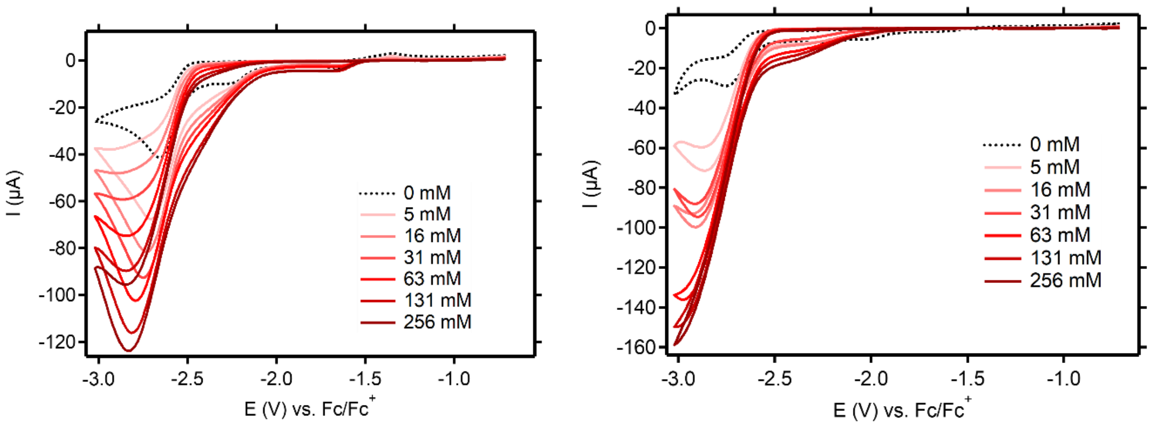

Figure S34: CV traces of the reduction of DCM catalysed by **[Ni(^{tBu}PEP)$_2$Cl$_2$]** (left) and **[Ni(^{tBu}PPBQ)$_2$Cl$_2$]** (right). For these plots, the currents of the blank measurements without added catalysts were subtracted from the currents obtained with catalyst present. Measurements were performed in THF, 0.2 M $N^nBu_4PF_6$ using a glassy carbon WE, a Pt wire CE and a $Ag/AgNO_3$ (saturated solution in the electrolyte) RE.

Measurements were also performed at low catalyst concentrations of 0.1-0.5 mM. Using these low concentrations allowed us to measure in the substrate concentration independent regime. At a substrate concentration of 128 mM, a linear dependence of the catalytic current on the catalyst concentration was determined for **[Ni(^{tBu}PEP)$_2$Cl$_2$]**, implying that the reaction is first order in catalyst (Figure S33d). For **[Ni(^{tBu}PPBQ)$_2$Cl$_2$]**, the current enhancement was still dependent on the substrate concentration, even at low catalyst concentrations, so no catalyst concentration dependence could accurately be measured.

Substrate scope and selectivity

To obtain more insight into the scope and mechanism of the catalytic activity of our nickel complexes, we investigated the reaction using a number of different substrates. These were investigated in THF solvent, using 1 mM of **[Ni(^{tBu}PEP)$_2$Cl$_2$]** and a range of substrate concentrations between 5 and 300 mM. Firstly, we investigated the activity towards a variety of alkyl halides. No or negligible catalytic activity above the background current was observed using 1-chloropropane, 2-chloropropane, cyclohexyl chloride or 1-chlorodecane. Similarly, benzyl chloride, dichlorodiphenylmethane and chlorodiphenylmethane all did not give any catalytic current, even though the benzylic carbon-chloride bonds are significantly weaker than aliphatic carbon-chloride bonds.[5] The aromatic chlorides chlorobenzene and *m*-dichlorobenzene also did not show any reactivity. Compounds with carbon-bromide bonds – dibromomethane and benzyl bromide – also showed no turnover.

Next, we considered the involvement of nickel carbenes[6] in the reaction pathway. To this end, we investigated the reduction of 1,2-dichloroethane (DCE). While electronically and sterically similar to DCM, there is no obvious pathway towards nickel carbene species with DCE as there is with DCM. Interestingly however, activity was observed for both complexes, albeit lower than what was observed for DCM. Continuing the series, 1,3-dichloropropane (DCP) also shows catalytic currents with both complexes, again lower than observed for DCE, and 1,4-dichlorobutane (DCB) shows essentially no current enhancement with complex **[Ni(^{tBu}PEP)$_2$Cl$_2$]** but a slight increase for **[Ni(^{tBu}PPBQ)$_2$Cl$_2$]** (S35, S36, S37). While the nickel centre is relatively shielded with a buried volume[7,8] of 71.9% (compared to Ni(II)-salen[9] with 63.6%), it seems unlikely that the small changes in steric bulk between DCM, dibromomethane, and the dichloroalkanes result in such dramatic changes in activity. It is worth noting

that the decrease in catalytic current with increasing carbon chain length is also observed in the blank measurements without catalyst present (on the carbon electrode, Figure S38).

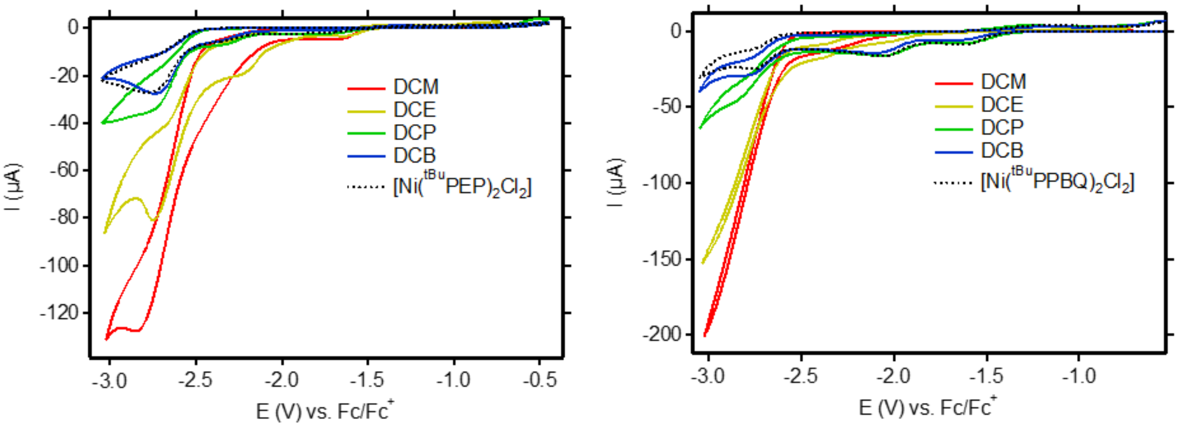

Figure S35: CV traces of **[Ni(^{tBu}PEP)$_2$Cl$_2$]** (left) and **[Ni(^{tBu}PPBQ)$_2$Cl$_2$]** (right) with 120-140 mM of different chloroalkyl substrates.

We then turned to constant potential electrolysis (CPE) to hopefully allow for identification of products. However, under catalytically relevant conditions, currents dropped significantly within several minutes (Figure S39). This was attributed to complete passivation of the electrode, as a CV of the solution after CPE did not reveal any redox couples or catalysis. Additionally, the activity was mostly regained after polishing the electrode. Repeated polishing/CPE cycles (Figure S40) consistently led to discolouration of the catholyte solution, and post-catalysis ^{1}H NMR confirmed the formation of free ligand in the catholyte (Figure S28), with no evidence for reactivity at the ligand itself (*e.g.* chlorination). However, catalytic currents remained too low under these conditions to measure potential gaseous products using conventional GC methods. Changing the working electrode to platinum also led to electrode passivation during CPE (Figure S39). Given the low currents, quick passivation and catalyst decomposition, no products of catalytic dehalogenation could be identified in the catholyte.

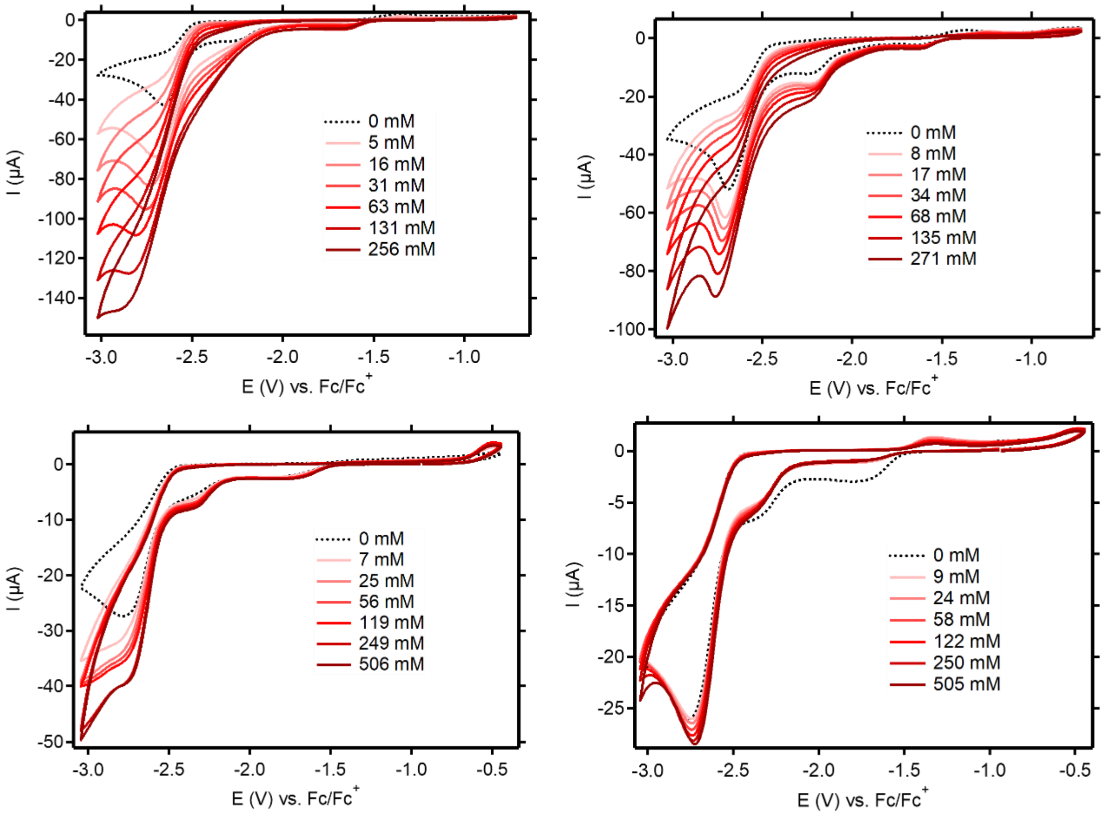

Figure S36: CV traces of the reduction of DCM (top, left), DCE (top, right), DCP (bottom, left) and DCB (bottom, right) catalysed by **[Ni(^{tBu}PEP)$_2$Cl$_2$]**. Measurements were performed in THF, 0.2 M $N^nBu_4PF_6$ using a glassy carbon WE, a Pt wire CE and a Ag/$AgNO_3$ (saturated solution in the electrolyte) RE.

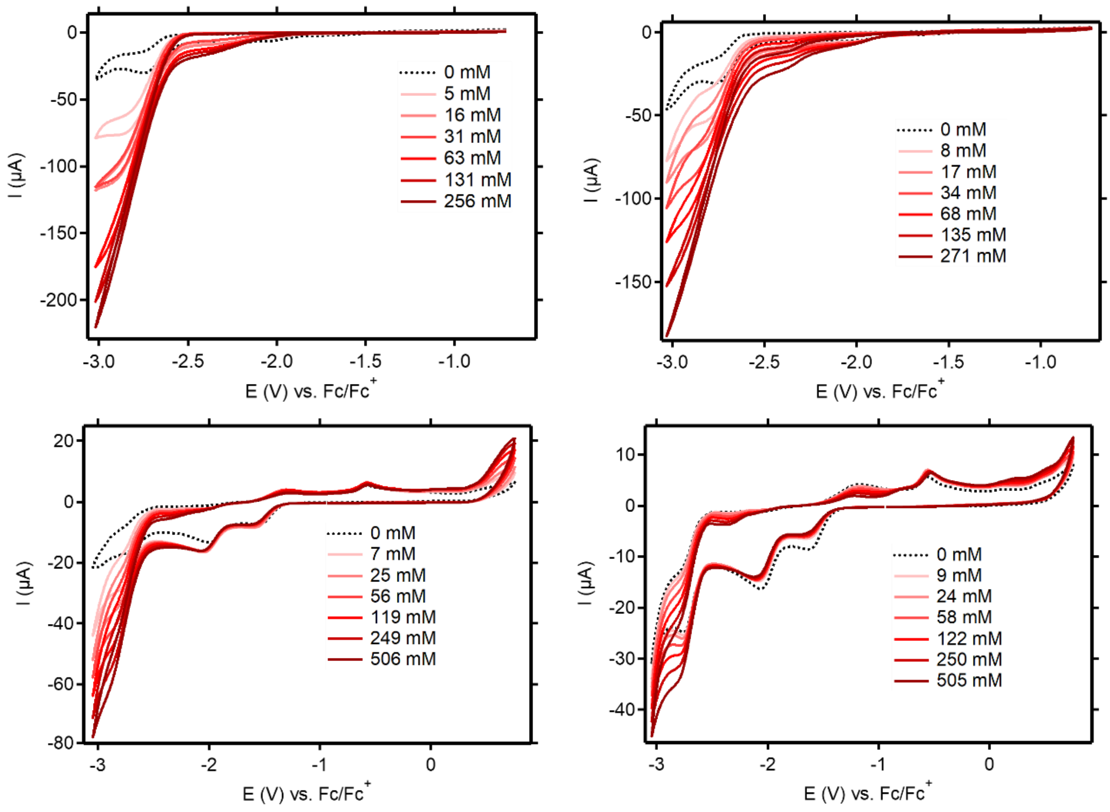

Figure S37: CV traces of the reduction of DCM (top, left), DCE (top, right), DCP (bottom, left) and DCB (bottom, right) catalysed by **[Ni(^{tBu}PPBQ)$_2$Cl$_2$]**. Measurements were performed in THF, 0.2 M $N^nBu_4PF_6$ using a glassy carbon WE, a Pt wire CE and a $Ag/AgNO_3$ (saturated solution in the electrolyte) RE.

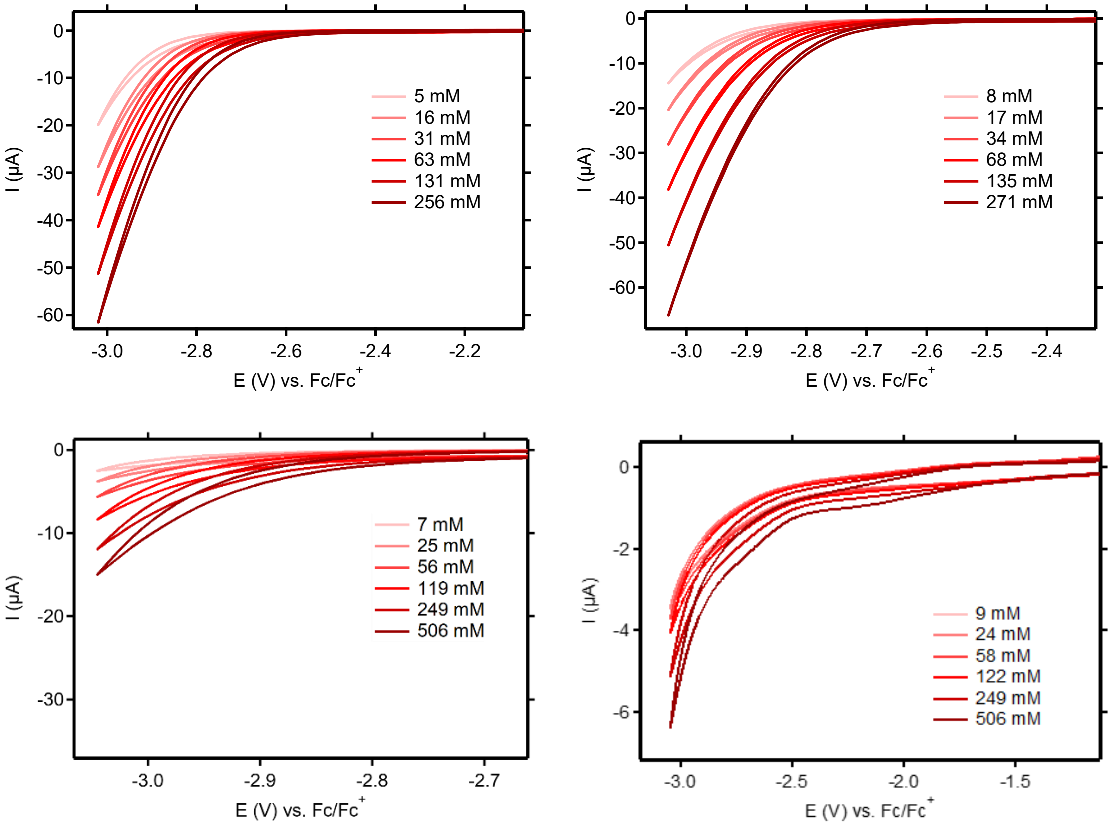

Figure S38: CV traces of the reduction of DCM (top, left), DCE (top, right), DCP (bottom, left) and DCB (bottom, right) on a glassy carbon electrode (blanks). Measurements were performed in THF, 0.2 M $N^nBu_4PF_6$ using a glassy carbon WE, a Pt wire CE and a $Ag/AgNO_3$ (saturated solution in the electrolyte) RE.

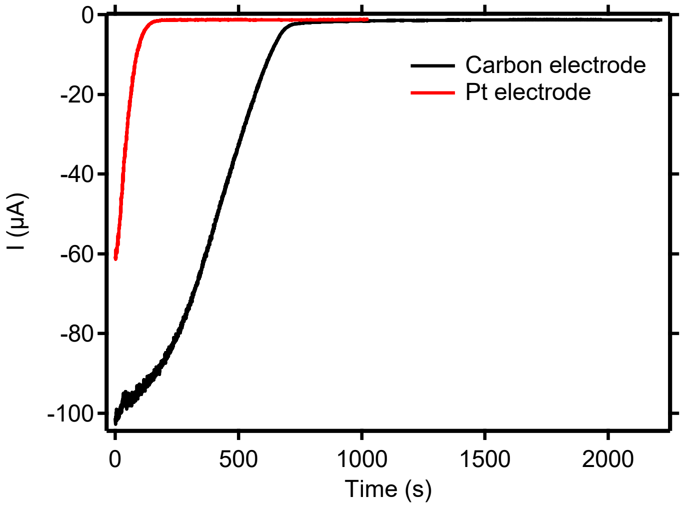

Figure S39: Plots of the CPE on either a carbon or a platinum electrode (3 mm Ø). Both experiments use 261 mM DCM as substrate, and ~0.5 mM or 1 mM **[Ni(^{tBu}PEP)$_2$Cl$_2$]** for the measurements on carbon or Pt, respectively. A potential of -2.76 or -2.43 V vs. Fc/Fc^+ was maintained throughout the experiment for the experiment with a carbon or Pt electrode, respectively. Measurements were performed in an H-cell with ~3 mL volume for both the catholyte and anolyte compartments. During CPE, both catholyte and anolyte were continuously stirred using a Teflon-coated stir bar. Measurements were performed in THF, 0.2 M $N^nBu_4PF_6$ using a Pt wire CE and a $Ag/AgNO_3$ (saturated solution in the electrolyte) RE.

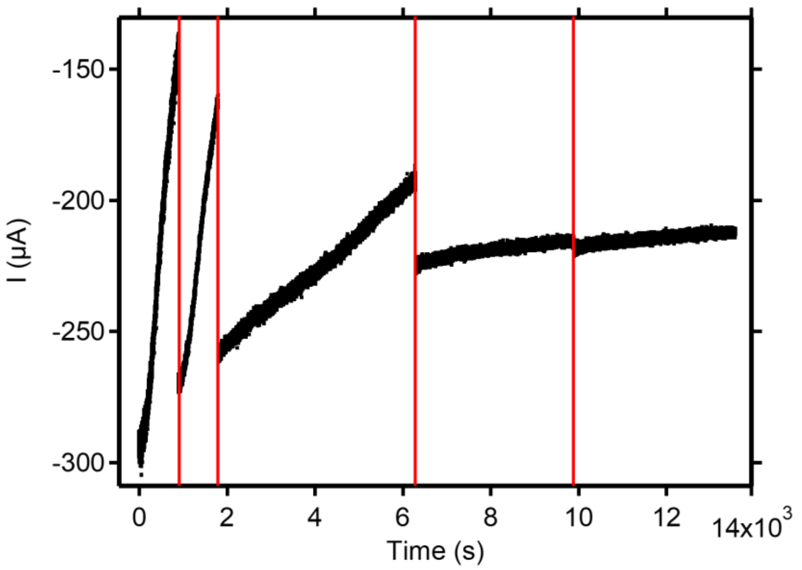

Figure S40: Approximately 4 hours of CPE with 30 mM DCM as substrate and 3 mM **[Ni(^{tBu}PEP)$_2$Cl$_2$]** as catalyst. A potential of -2.83 V vs. Fc/Fc$^+$ was maintained throughout the experiment. The red lines indicate where the working electrodes were switched, and the used electrode was polished briefly for the next CPE measurement. Measurements were performed in an H-cell with ~3 mL volume for both the catholyte and anolyte compartments. During CPE, both catholyte and anolyte were continuously stirred using a Teflon-coated stir bar. Measurements were performed in THF, 0.2 M N^{n}Bu$_4$PF$_6$ using a Pt wire CE and a Ag/AgNO$_3$ (saturated solution in the electrolyte) RE.

S6. X-ray crystal structure determinations

[Zn(^{tBu}PEP)Cl_2]

$C_{32}H_{34}Cl_2N_2Zn$, Fw = 582.88, colourless needle, 0.56 × 0.11 × 0.07 mm^3, triclinic, $P\bar{1}$ (no. 2), a = 12.8450(5), b = 15.5635(6), c = 17.1300(6) Å, α = 94.992(2), β = 105.904(2), γ = 109.654(2) °, V = 3040.2(2) $Å^3$, Z = 4, D_x = 1.273 g/cm^3, μ = 1.01 mm^{-1}. The diffraction experiment was performed on a Bruker Kappa ApexII diffractometer with sealed tube and Triumph monochromator (λ = 0.71073 Å) at a temperature of 150(2) K up to a resolution of $(\sin \theta/\lambda)_{max}$ = 0.61 $Å^{-1}$. The Eval15 software[10] was used for the intensity integration. The prediction of the reflection profiles involved a split-mosaic model[11]. A numerical absorption correction and scaling was performed with SADABS[12] (correction range 0.61-1.00). A total of 60479 reflections was measured, 11335 reflections were unique (R_{int} = 0.049), 8215 reflections were observed [$I>2\sigma(I)$]. The structure was solved with Patterson superposition methods using SHELXT.[13] Because of *pseudo*-translational symmetry, reflections *hkl* with *k=odd* are weak. Structure refinement was performed with SHELXL-2018[14] on F^2 of all reflections. Non-hydrogen atoms were refined freely with anisotropic displacement parameters. All hydrogen atoms were located in difference Fourier maps and refined with a riding model. 679 Parameters were refined with no restraints. R1/wR2 [$I > 2\sigma(I)$]: 0.0348 / 0.0828. R1/wR2 [all refl.]: 0.0595 / 0.0928. S = 1.041. Residual electron density between -0.40 and 0.37 e/$Å^3$. Geometry calculations and checking for higher symmetry was performed with the PLATON program.[15]

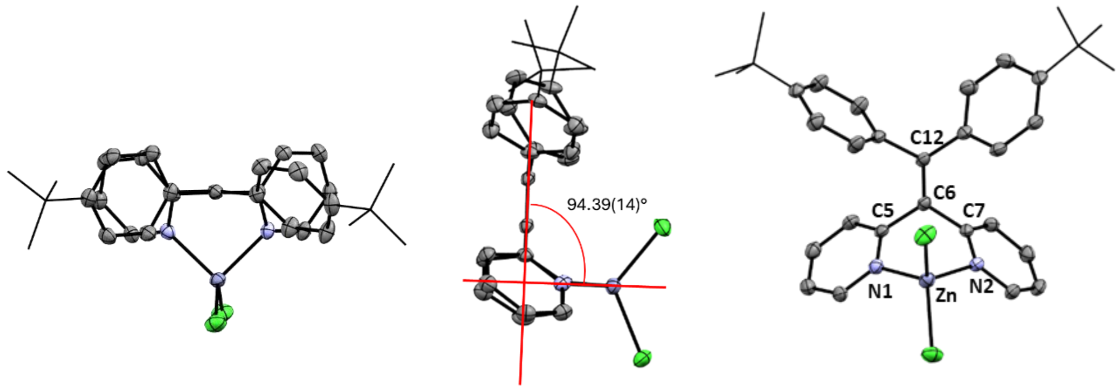

Figure S41: Displacement ellipsoid plot (50 % probability) of the asymmetric unit of **[Zn(^{tBu}PEP)Cl_2]**. The hydrogen atoms are omitted and the *tert*-butyl groups are depicted as wireframe for clarity. The asymmetric unit contains two independent molecules, of which only one is shown. The angle shown is calculated as the angle between the central double bond (defined by the average plane through C5, C6, C7 and C12) and the plane defined by N1, Zn1 and N2.

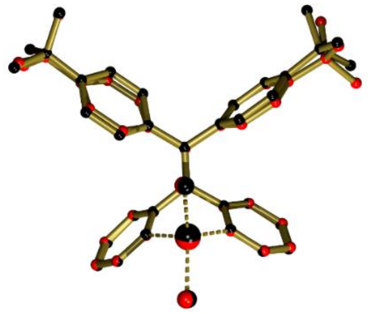

Figure S42**:** Overlay-plot of the two independent molecules in crystal structure **[Zn(^{tBu}PEP)Cl_2]** showing the small difference.

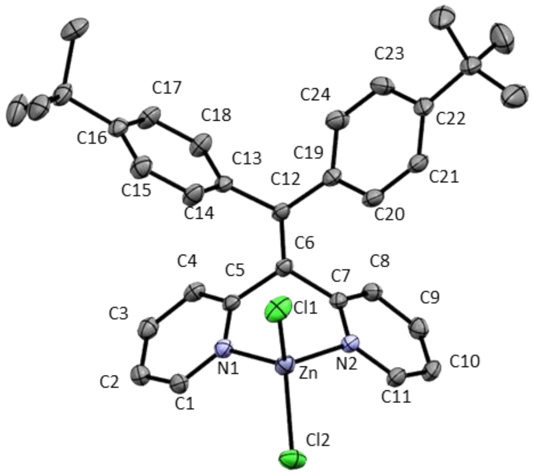

Figure S43: Labelled displacement ellipsoid plot (50 % probability) of the asymmetric unit of **[Zn(^{tBu}PEP)Cl_2]**. The hydrogen atoms are omitted for clarity. The asymmetric unit shows two independent molecules, of which only one is shown.

Table S1**:** Selected (bond) distances [Å] and angles [°] in the crystal structure **[Zn(^{tBu}PEP)Cl_2]**.

**Distance**	**x=1**	**x=2**	**Angle**	**x=1**	**x=2**
Zn(x)-Cl1(x)	2.2045(7)	2.1995(7)	Cl1(x)-Zn(x)-N1(x)	113.22(6)	113.27(6)
Zn(x)-Cl2(x)	2.2164(7)	2.2164(7)	Cl1(x)-Zn(x)-Cl2(x)	118.45(3)	118.60(3)
Zn(x)-N1(x)	2.051(2)	2.046(2)	N1(x)-Zn(x)-N2(x)	91.16(8)	91.54(8)
Zn(x)-N2(x)	2.064(2)	2.061(2)	Zn(x)-N1(x)-C5(x)	118.27(15)	118.15(16)
N1(x)-N2(x)	2.939(3)	2.943(3)	Zn(x)-N2(x)-C7(x)	117.61(15)	116.43(15)
N1(x)-C5(x)	1.350(3)	1.352(3)	N1(x)-C5(x)-C6(x)	116.6(2)	115.5(2)
N2(x)-C7(x)	1.350(3)	1.354(3)	N2(x)-C7(x)-C6(x)	116.6(2)	116.6(2)
C5(x)-C6(x)	1.493(3)	1.500(3)	C5(x)-C6(x)-C12(x)	121.8(2)	122.0(2)
C6(x)-C7(x)	1.503(3)	1.497(3)	C7(x)-C6(x)-C12(x)	124.3(2)	125.5(2)
C6(x)-C12(x)	1.333(3)	1.342(3)	C5(x)-C6(x)-C7(x)	113.9(2)	112.51(19)
C12(x)-C13(x)	1.509(3)	1.502(3)	C6(x)-C12(x)-C13(x)	120.2(2)	120.1(2)
C12(x)-C19(x)	1.502(3)	1.491(3)	C6(x)-C12(x)-C19(x)	123.2(2)	123.6(2)
			C13(x)-C12(x)-C19(x)	116.2(2)	115.8(2)

[Zn(^{tBu}PPBQ)Cl$_2$]

$C_{32}H_{32}Cl_2N_2Zn \cdot C_2H_6O$, Fw = 626.93, colourless needle, 0.27 × 0.07 × 0.04 mm^3, triclinic, P $\overline{1}$ (no. 2), a = 8.9655(7), b = 13.7579(11), c = 14.1054(8) Å, α = 107.060(3), β = 97.229(2), γ = 102.061(2) °, V = 1593.7(2) Å^3, Z = 2, D_x = 1.306 g/cm^3, μ = 0.97 mm^{-1}. The diffraction experiment was performed on a Bruker Kappa ApexII diffractometer with sealed tube and Triumph monochromator (λ = 0.71073 Å) at a temperature of 150(2) K up to a resolution of $(\sin \theta/\lambda)_{max}$ = 0.65 Å^{-1}. The crystal appeared to be cracked into three fragments with rotation angles of 6.6 and 4.6 ° between them. Consequently, three orientation matrices were used for the intensity integration with the Eval15 software[10]. A multi-scan absorption correction and scaling was performed with TWINABS[16] (correction range 0.51-0.75). A total of 92410 reflections was measured, 7405 reflections were unique (R_{int} = 0.091), 4616 reflections were observed [I>2σ(I)]. The structure was solved with Patterson superposition methods using SHELXT.[13] Structure refinement was performed with SHELXL-2018[14] on F^2 of all reflections. Non-hydrogen atoms were refined freely with anisotropic displacement parameters. The t-butyl groups and the ethanol solvent molecule were refined with disorder models. O-H hydrogen atoms were located in difference Fourier maps and kept fixed on these positions. C-H hydrogen atoms were introduced in calculated positions and refined with a riding model. 447 Parameters were refined with 348 restraints (geometry and displacement parameters of the disordered moieties). R1/wR2 [I > 2σ(I)]: 0.0540 / 0.1263. R1/wR2 [all refl.]: 0.1016 / 0.1467. S = 1.041. Residual electron density between -0.59 and 0.41 e/Å^3. Scale factors for the second and third crystal fragments BASF = 0.383(4) and 0.075(4). Geometry calculations and checking for higher symmetry was performed with the PLATON program.[15]

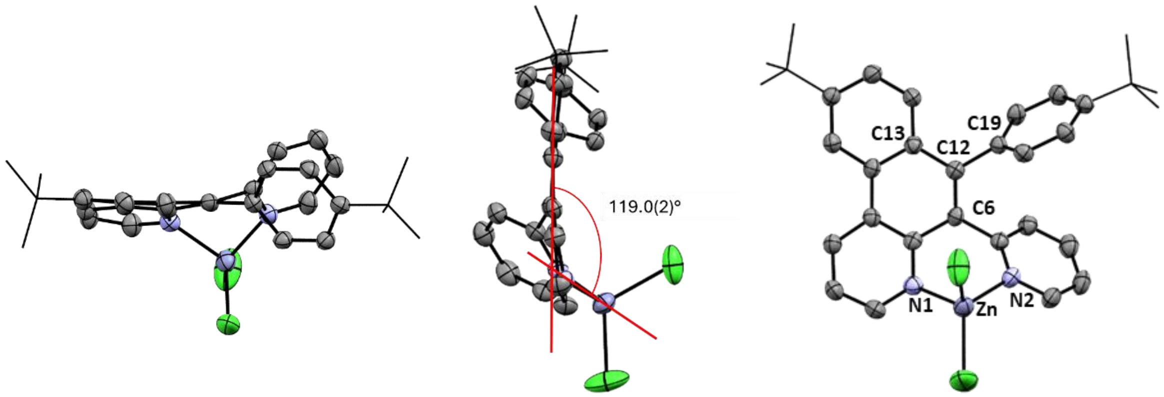

Figure S44: Displacement ellipsoid plot (50 % probability) of the asymmetric unit of **[Zn(^{tBu}PPBQ)Cl$_2$]**. The hydrogen atoms and a co-crystallized EtOH molecule are omitted and the *tert*-butyl groups are depicted as wireframe for clarity. Only the major disorder component is shown. The angle shown is calculated as the angle between the central carbon-carbon bond (defined by the average plane through C6, C12, C13 and C19) and the plane defined by N1, Zn and N2 to 119.0(2)°

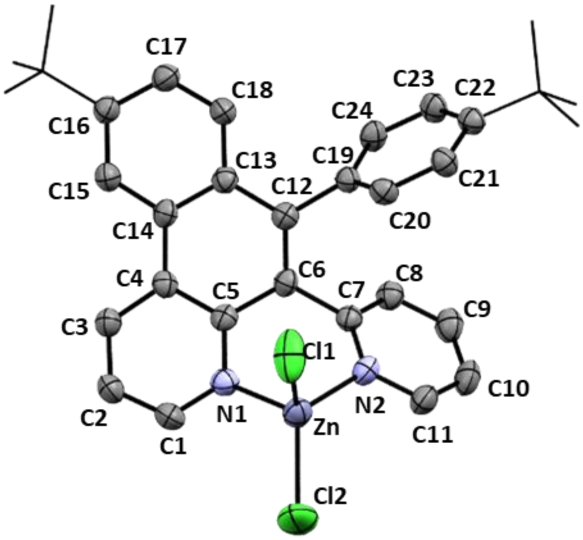

Figure S45: Labelled displacement ellipsoid plot (50 % probability) of the asymmetric unit of **[Zn(^{tBu}PPBQ)Cl$_2$]**. The hydrogen atoms and a co-crystallized EtOH molecule are omitted for clarity.

Table S2: Selected (bond) distances [Å] and angles [°] in the crystal structure **[Zn(^{tBu}PPBQ)Cl$_2$]**.

Bond	Distance (Å)	Atoms	Angle (°)
Zn1-Cl1	2.2243(12)	Cl1-Zn1-N1	109.36(8)
Zn1-Cl2	2.1918(12)	Cl1-Zn1-Cl2	117.02(6)
Zn1-N1	2.076(3)	N1-Zn1-N2	90.16(11)
Zn1-N2	2.021(3)	Zn1-N1-C5	116.1(2)
N1-N2	2.901(4)	Zn1-N2-C7	118.6(2)
N1-C5	1.361(4)	N1-C5-C6	118.5(3)
N2-C7	1.340(4)	N2-C7-C6	121.0(3)
C5-C6	1.452(4)	C5-C6-C12	118.9(3)
C6-C7	1.504(4)	C7-C6-C12	119.7(3)
C6-C12	1.367(5)	C5-C6-C7	121.0(3)
C12-C13	1.446(4)	C6-C12-C13	121.3(3)
C12-C19	1.498(4)	C6-C12-C19	119.7(3)
C4-C14	1.455(5)	C13-C12-C19	119.0(3)

[Ni(^{tBu}PEP)$_2$Cl$_2$]

$C_{64}H_{68}Cl_2N_4Ni \cdot 6CH_2Cl_2$, Fw = 1532.39, light green block, 0.51 × 0.19 × 0.13 mm^3, triclinic, P $\overline{1}$ (no. 2), a = 9.0227(3), b = 12.0364(4), c = 18.7791(5) Å, α = 102.479(1), β = 93.579(2), γ = 108.074(1) °, V = 1874.50(10) Å^3, Z = 1, D_x = 1.357 g/cm^3, μ = 0.80 mm^{-1}. The diffraction experiment was performed on a Bruker Kappa ApexII diffractometer with sealed tube and Triumph monochromator (λ = 0.71073 Å) at a temperature of 150(2) K up to a resolution of $(\sin \theta/\lambda)_{max}$ = 0.65 Å^{-1}. The Eval15 software[10] was used for the intensity integration. A numerical absorption correction and scaling was performed with SADABS[12] (correction range 0.76-0.96). A total of 50304 reflections was measured, 8627 reflections were unique (R_{int} = 0.034), 7288 reflections were observed [I>2σ(I)]. The structure was solved with Patterson superposition methods using SHELXT.[13] Structure refinement was performed with SHELXL-2018[14] on F^2 of all reflections. Non-hydrogen atoms were refined freely with anisotropic displacement parameters. Two of the CH_2Cl_2 solvent molecules were refined with a disorder model. The disorder in the solvent molecules could not be fully resolved and minor disorder in the t-butyl groups has been ignored. Hydrogen atoms were introduced in calculated positions and refined with a riding model. 447 Parameters were refined with 157 restraints (geometry and displacement parameters of the CH_2Cl_2 molecules). R1/wR2 [I > 2σ(I)]: 0.0461 / 0.1249. R1/wR2 [all refl.]: 0.0557 / 0.1297. S = 1.037. Residual electron density between -0.64 and 1.17 e/Å^3 (in proximity of the CH_2Cl_2 molecules). Geometry calculations and checking for higher symmetry was performed with the PLATON program.[15]

CCDC 2495948-2495950 contain the supplementary crystallographic data for this paper. These data

can be obtained free of charge from The Cambridge Crystallographic Data Centre via

www.ccdc.cam.ac.uk/data_request/cif.

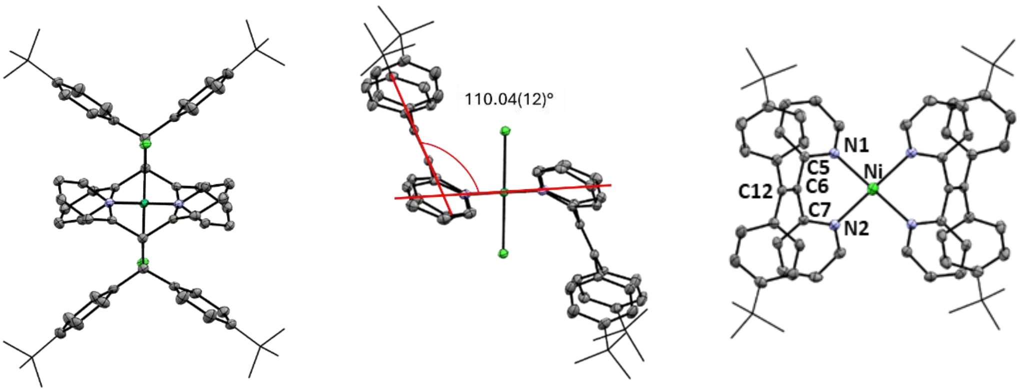

Figure S46: Displacement ellipsoid plot (50 % probability) of **[Ni(^{tBu}PEP)$_2$Cl$_2$]**. The hydrogen atoms and co-crystallized DCM molecules are omitted and the *tert*-butyl groups are depicted as wireframe for clarity. The nickel is on an inversion center. The angle shown was calculated as the angle between the central double bond (defined by the average plane through C5, C6, C7 and C12) and the plane defined by N1, Ni1 and N2.

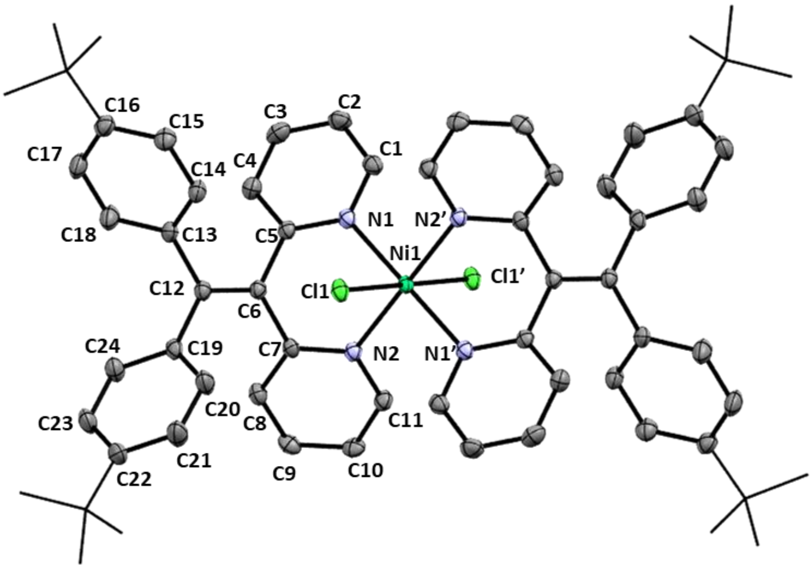

Figure S47: Labelled displacement ellipsoid plot (50 % probability) of the asymmetric unit of **[Ni(^{tBu}PEP)$_2$Cl$_2$]**. The hydrogen atoms and a co-crystallized DCM molecules are omitted and the *tert*-butyl groups are depicted as wireframe for clarity. The asymmetric unit cell shows half of the molecule pictured here, with an inversion center on nickel.

Table S3: Selected (bond) distances [Å] and angles [°] in the crystal structure **[Ni(^{tBu}PEP)$_2$Cl$_2$].** Symmetry code ‘: 1-x, 1-y, 1-z.

Bond	Distance (Å)	Atoms	Angle (°)
Ni1-Cl1	2.4373(5)	N1-Ni1-N2	85.45(7)
Ni1-N1	2.1140(18)	N1-Ni1-N2’	94.55(7)
Ni1-N2	2.1032(17)	Ni1-N1-C5	123.18(14)
N1-N1’	4.228(3)	Ni1-N2-C7	123.66(14)
N1-N2	2.862(3)	N1-C5-C6	118.89(18)
N1-N2’	3.098(3)	N2-C7-C6	118.64(17)
N2-N2’	4.206(3)	C5-C6-C12	121.32(18)
N1-C5	1.350(3)	C7-C6-C12	122.76(19)
N2-C7	1.349(3)	C5-C6-C7	115.54(17)
C5-C6	1.496(3)	C6-C12-C13	122.27(19)
C6-C7	1.497(3)	C6-C12-C19	122.88(19)
C6-C12	1.347(3)	C13-C12-C19	114.19(17)
C12-C13	1.500(4)		
C12-C19	1.501(3)		

S7. IR spectra

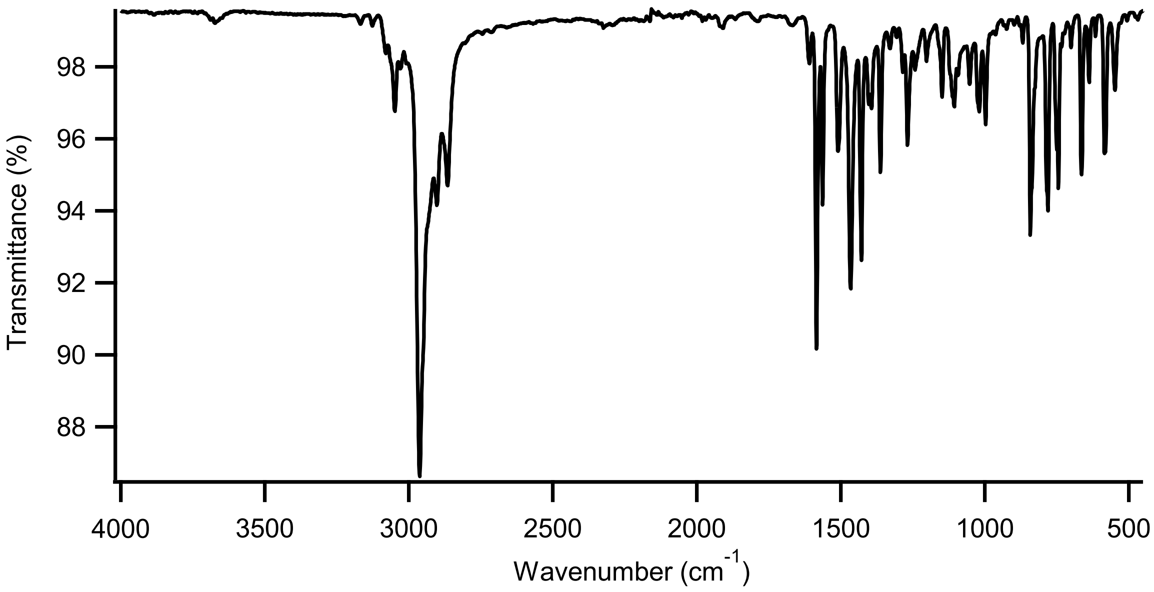

Figure S48: ATR-IR spectrum of 2,2'-(2,2-bis(4-(*tert*-butyl)phenyl)ethene-1,1-diyl)dipyridine (**^{tBu}PEP)**.

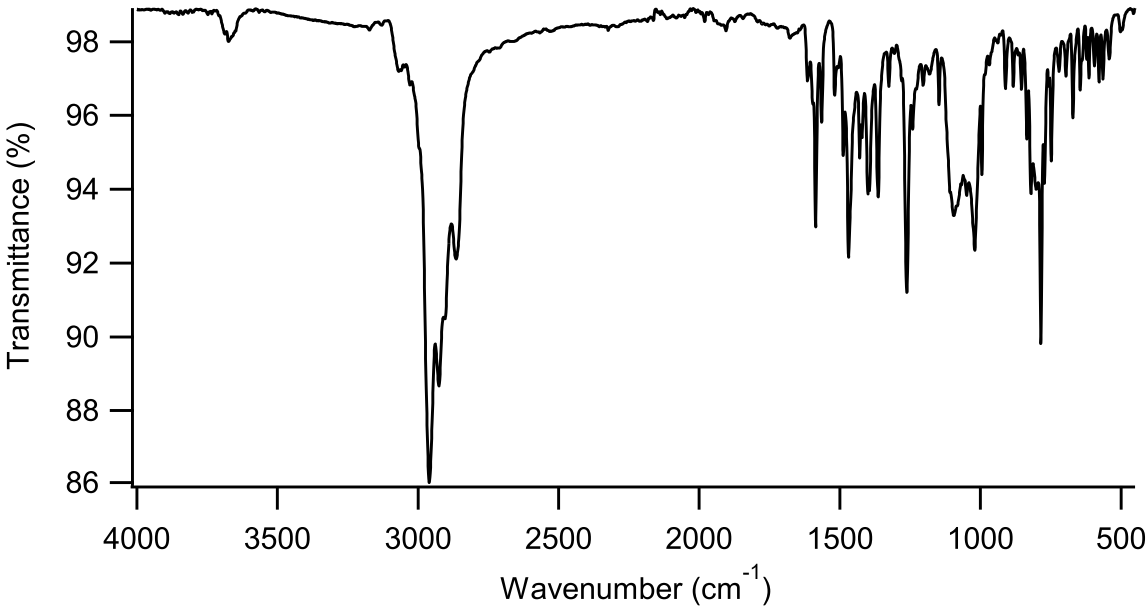

Figure S49: ATR-IR spectrum of 9-(*tert*-butyl)-6-(4-(*tert*-butyl)phenyl)-5-(pyridin-2-yl)benzo[*f*]quinoline (**^{tBu}PPBQ)**.

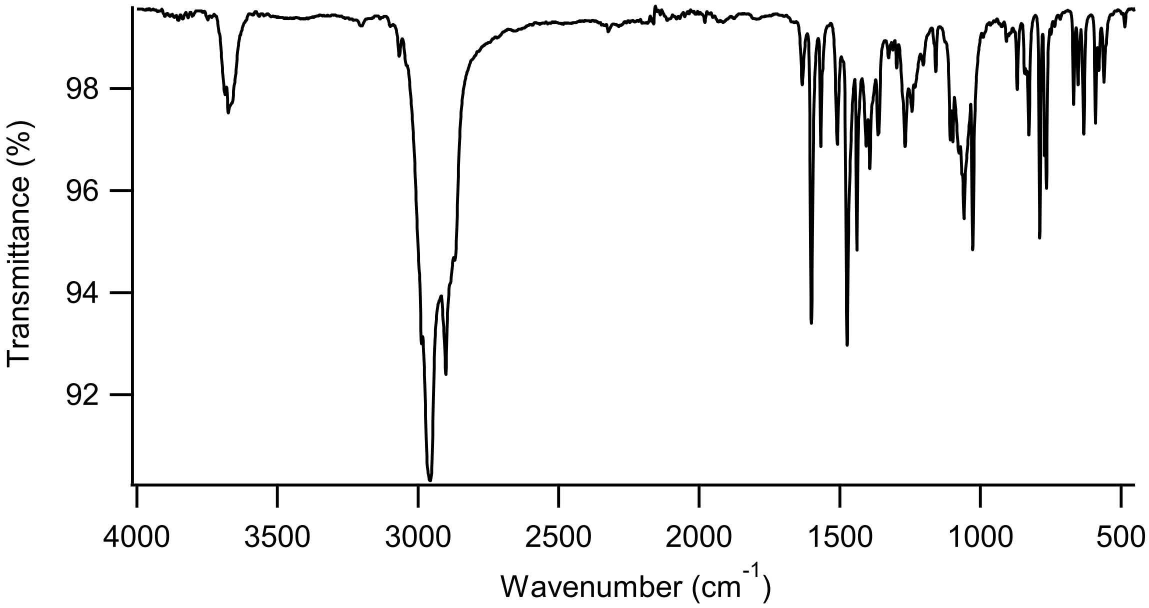

Figure S50: ATR-IR spectrum of **[Zn(^{tBu}PEP)Cl$_2$]**

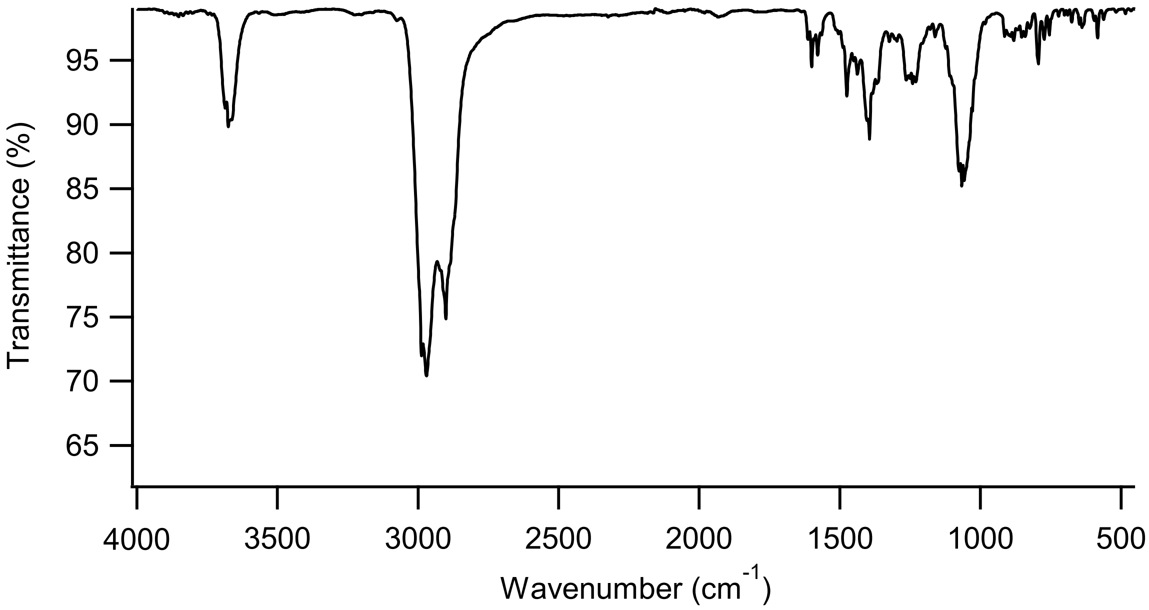

Figure S51: ATR-IR spectrum of **[Zn(^{tBu}PPBQ)Cl$_2$]**

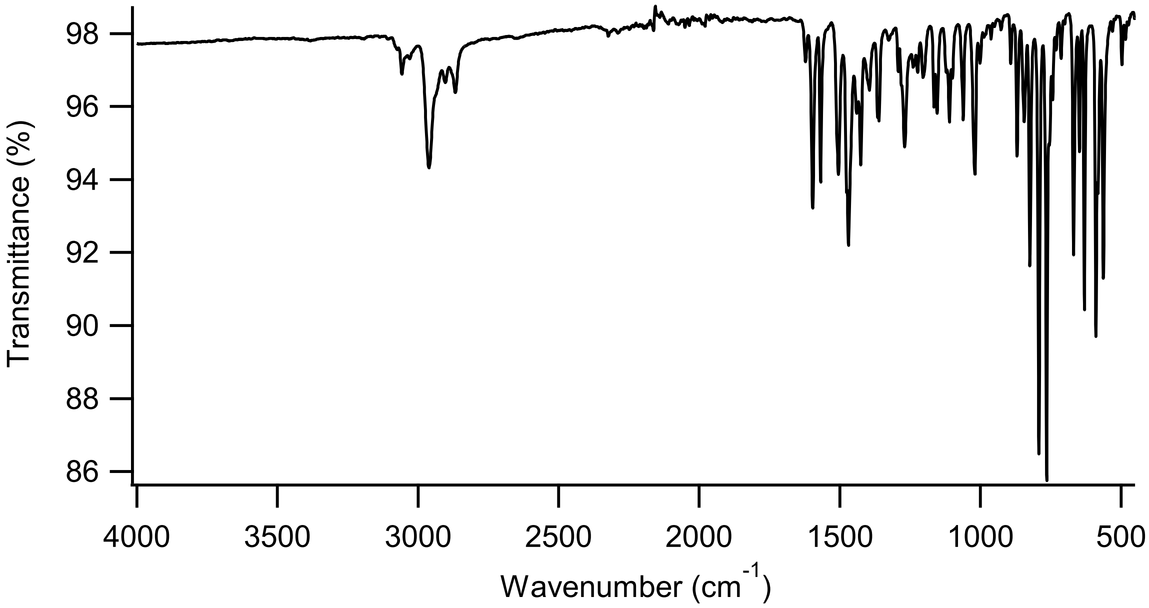

Figure S52: ATR-IR spectrum of **[Ni(^{tBu}PEP)$_2$Cl$_2$]**.

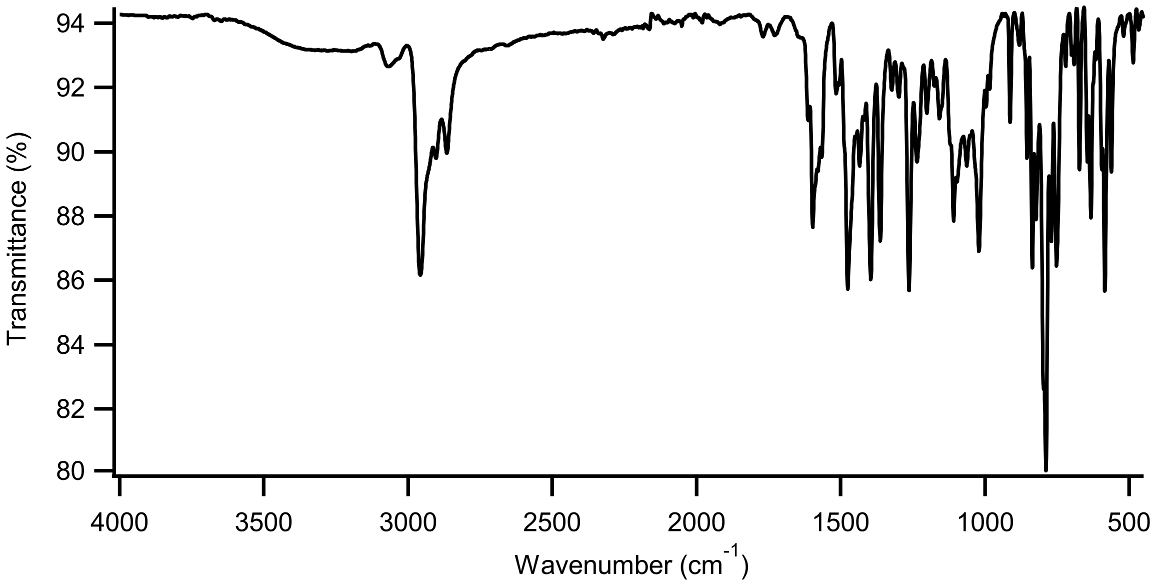

Figure S53: ATR-IR spectrum of **[Ni(^{tBu}PPBQ)$_2$Cl$_2$]**.

S8. HRMS spectra

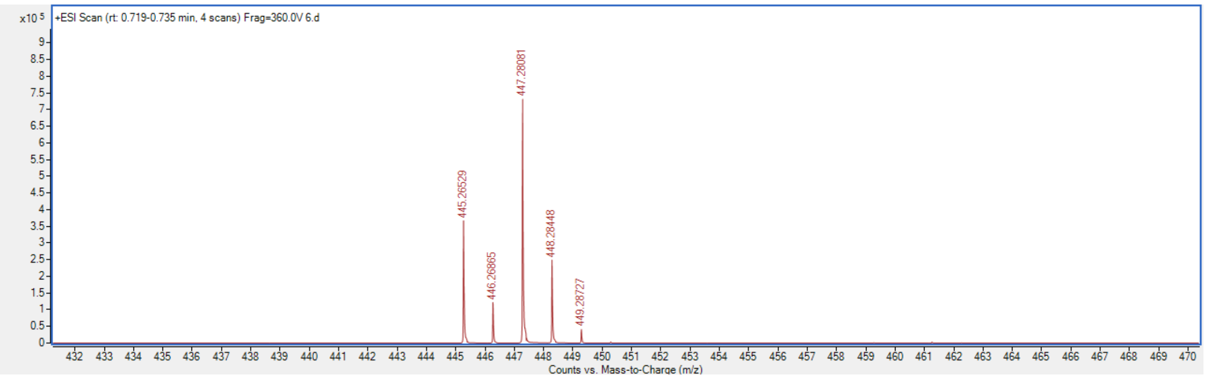

Figure S54: HRMS spectrum of ^{tBu}**PEP** {[M+H]$^+$, calc. 447.28002}. The peak m/z = 445.26529 corresponds to ^{tBu}**PPBQ** {[M+H]$^+$, calc. 445.26437}, possibly formed during the ionization.

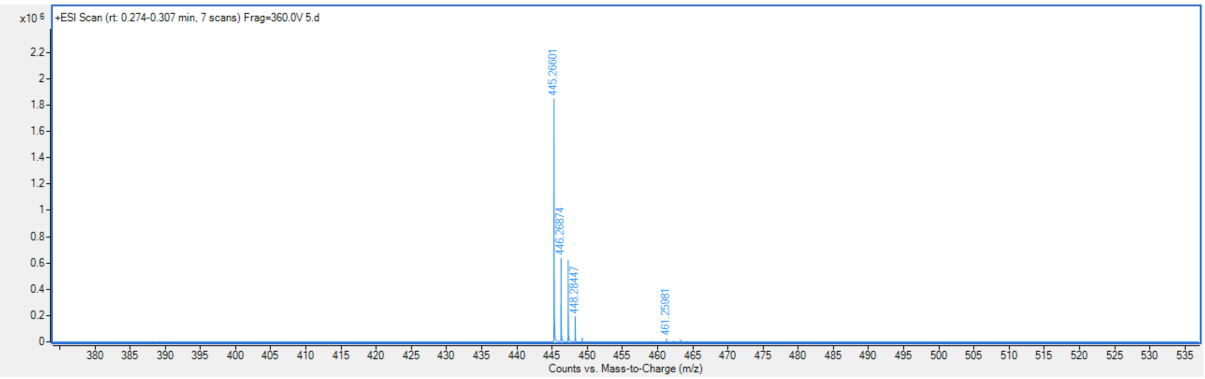

Figure S55: HRMS spectrum of ^{tBu}**PPBQ** {[M+H]$^+$, calc. 445.26437}.

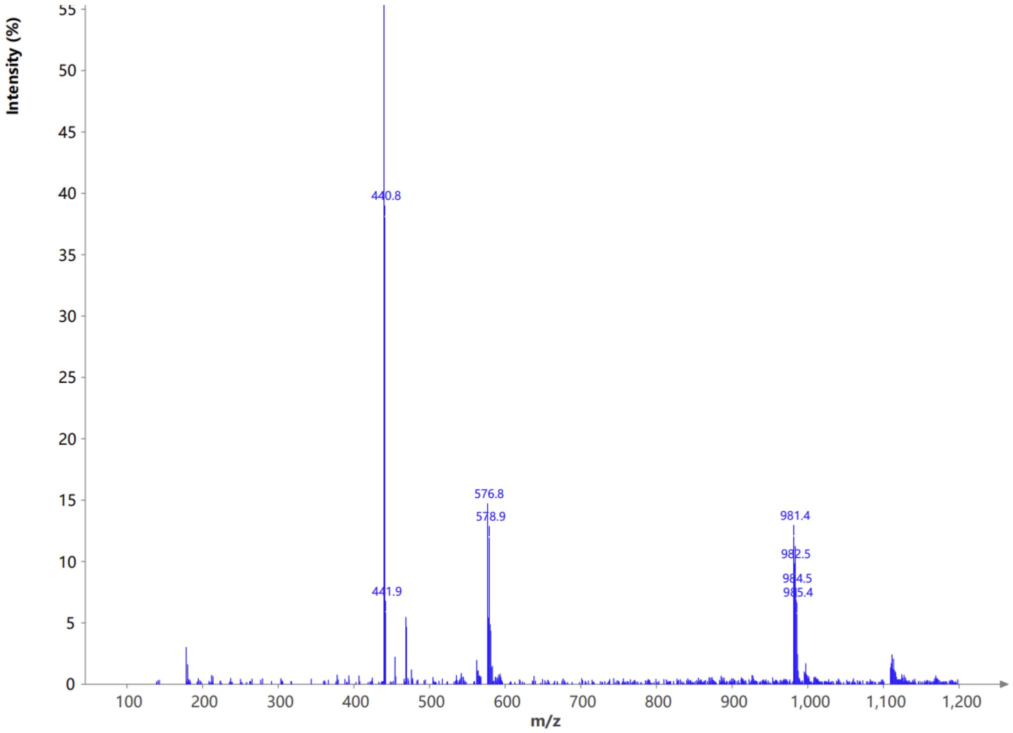

Figure S56: ESI-MS spectrum of **[Ni(^{tBu}PPBQ)$_2$Cl$_2$]** {[M-Cl]$^+$, calc. 981.4}. Note that this spectrum was measured on a low resolution machine (see S1. General Remarks), as on the high resolution machine only the free ligand was observed at 445.26910 {[M+H]$^+$, calc. 445.26437}.

References

1 G. R. Fulmer, A. J. M. Miller, N. H. Sherden, H. E. Gottlieb, A. Nudelman, B. M. Stoltz, J. E. Bercaw and K. I. Goldberg, *Organometallics*, 2010, **29**, 2176–2179.

2 M. T. Gabr and F. C. Pigge, *Dalton Trans.*, 2016, **45**, 14039–14043.

3 K. J. Lee, N. Elgrishi, B. Kandemir and J. L. Dempsey, *Nat. Rev. Chem.*, 2017, **1**, 0039.

4 E. S. Rountree, B. D. McCarthy, T. T. Eisenhart and J. L. Dempsey, *Inorg. Chem.*, 2014, **53**, 9983–10002.

5 C. Y. Lin, J. Peh and M. L. Coote, *J. Org. Chem.*, 2011, **76**, 1715–1726.

6 M. Liu, N. Le and C. Uyeda, *Angew. Chem. Int. Ed.*, 2023, **135**, e202308913.

7 L. Falivene, R. Credendino, A. Poater, A. Petta, L. Serra, R. Oliva, V. Scarano and L. Cavallo, *Organometallics*, 2016, **35**, 2286–2293.

8 L. Falivene, Z. Cao, A. Petta, L. Serra, A. Poater, R. Oliva, V. Scarano and L. Cavallo, *Nat. Chem.*, 2019, **11**, 872–879.

9 M. A. Siegler and M. Lutz, *Cryst. Growth Des.*, 2009, **9**, 1194–1200.

10 A. M. M. Schreurs, X. Xian and L. M. J. Kroon-Batenburg, *J Appl Crystallogr*, 2010, **43**, 70–82.

11 A. J. M. Duisenberg, *Acta Crystallogr A Found Crystallogr*, 1983, **39**, 211–216.

12 L. Krause, R. Herbst-Irmer, G. M. Sheldrick and D. Stalke, *J Appl Crystallogr*, 2015, **48**, 3–10.

13 G. M. Sheldrick, *Acta Crystallogr A Found Adv*, 2015, **71**, 3–8.

14 G. M. Sheldrick, *Acta Crystallogr C Struct Chem*, 2015, **71**, 3–8.

15 A. L. Spek, *Acta Crystallogr D Biol Crystallogr*, 2009, **65**, 148–155.

16 M. Sevvana, M. Ruf, I. Usón, G. M. Sheldrick and R. Herbst-Irmer, *Acta Crystallogr D Struct Biol*, 2019, **75**, 1040–1050.
